# High-resolution isotopic data link settlement complexification to infant diets within the Roman Empire

**DOI:** 10.1093/pnasnexus/pgae566

**Published:** 2025-01-14

**Authors:** Carlo Cocozza, Alison J T Harris, Giulia Formichella, Giulia Pedrucci, Paola F Rossi, Alessandro D’Alessio, Valeria Amoretti, Gabriel Zuchtriegel, Michelle O’Reilly, Noemi Mantile, Sofia Panella, Mary A Tafuri, Simona Altieri, Maria R di Cicco, Ricardo Fernandes, Carmine Lubritto

**Affiliations:** Department of Archaeology, Max Planck Institute of Geoanthropology, Kahlaische Str. 10, Jena 07745, Germany; Dipartimento di Scienze e Tecnologie Ambientali Biologiche e Farmaceutiche (DiSTABiF) and Mediterranean bioArchaeological Research Advances (MAReA) centre, Università degli studi della Campania “Luigi Vanvitelli”, Via Vivaldi 43, Caserta 81100, Italy; ArchaeoBioCenter (ABC), Ludwig-Maximilians-Universität München, Geschwister-Scholl-Platz 1, München 80539, Germany; Department of Archaeology, Max Planck Institute of Geoanthropology, Kahlaische Str. 10, Jena 07745, Germany; Department of Archaeology, Memorial University of Newfoundland and Labrador, St. John's, NL, Canada A1B 3R6; Dipartimento di Biologia Ambientale and Mediterranean bioArchaeological Research Advances (MAReA) centre, Sapienza University of Rome, Piazzale Aldo Moro 5, Roma 00185, Italy; Dipartimento Di Culture e Civiltà, University of Verona, Via dell’Università, 4, Verona 37129, Italy; Parco archeologico di Ostia antica, MIC Viale dei Romagnoli 717, Roma 00119, Italy; Parco archeologico di Ostia antica, MIC Viale dei Romagnoli 717, Roma 00119, Italy; Parco Archeologico di Pompei, P.zza Porta Marina Inferiore 7, Pompei 80045, Italy; Parco Archeologico di Pompei, P.zza Porta Marina Inferiore 7, Pompei 80045, Italy; Department of Archaeology, Max Planck Institute of Geoanthropology, Kahlaische Str. 10, Jena 07745, Germany; Dipartimento di Scienze e Tecnologie Ambientali Biologiche e Farmaceutiche (DiSTABiF) and Mediterranean bioArchaeological Research Advances (MAReA) centre, Università degli studi della Campania “Luigi Vanvitelli”, Via Vivaldi 43, Caserta 81100, Italy; Dipartimento di Biologia Ambientale and Mediterranean bioArchaeological Research Advances (MAReA) centre, Sapienza University of Rome, Piazzale Aldo Moro 5, Roma 00185, Italy; Dipartimento di Biologia Ambientale and Mediterranean bioArchaeological Research Advances (MAReA) centre, Sapienza University of Rome, Piazzale Aldo Moro 5, Roma 00185, Italy; Dipartimento di Scienze e Tecnologie Ambientali Biologiche e Farmaceutiche (DiSTABiF) and Mediterranean bioArchaeological Research Advances (MAReA) centre, Università degli studi della Campania “Luigi Vanvitelli”, Via Vivaldi 43, Caserta 81100, Italy; Dipartimento di Scienze e Tecnologie Ambientali Biologiche e Farmaceutiche (DiSTABiF) and Mediterranean bioArchaeological Research Advances (MAReA) centre, Università degli studi della Campania “Luigi Vanvitelli”, Via Vivaldi 43, Caserta 81100, Italy; Department of Archaeology, Max Planck Institute of Geoanthropology, Kahlaische Str. 10, Jena 07745, Germany; Faculty of Archaeology, University of Warsaw, Krakowskie Przedmieście 26/28, Warsaw 00-927, Poland; Arne Faculty of Arts, Masaryk University, Nováka 1, Brno-střed 602 00, Czech Republic; Climate Change and History Research Initiative, Princeton University, Princeton, NJ 08544, USA; Dipartimento di Scienze e Tecnologie Ambientali Biologiche e Farmaceutiche (DiSTABiF) and Mediterranean bioArchaeological Research Advances (MAReA) centre, Università degli studi della Campania “Luigi Vanvitelli”, Via Vivaldi 43, Caserta 81100, Italy

**Keywords:** infant feeding practices, Roman Empire, incremental dentine stable carbon and nitrogen isotope analysis, Bayesian modeling, settlement complexification

## Abstract

Our study explores the potential relationship between infant feeding practices and settlement complexity in the Roman Empire through high-resolution Bayesian-modeled stable isotope measurements from incremental dentine. We compiled isotopic data from permanent first molars of individuals from various Roman sites: five from Bainesse (UK), 30 from Thessaloniki (Greece), along with new carbon and nitrogen isotope analyses from four individuals from Pompeii and six from Ostia Via del Mare (AVM). Our results reveal significant inter-site variability in breastfeeding durations, ranging from 1.5 years to approximately 5 years. Notably, individuals from the highly complex urban centers of Pompeii and Thessaloniki ceased breastfeeding around or below the 2-year weaning threshold recommended by Roman physicians. In contrast, individuals from the rural site of Ostia AVM and the site of Bainesse, near the northern frontier of the Roman Empire, generally ceased breastfeeding after 2 years of age. The link between settlement complexity and duration of breastfeeding observed in our study may have resulted from adherence to medical guidelines, support infrastructures, and/or strategies to mitigate financial constraints within households.

**Significance Statement**

We investigated how infant feeding practices varied with settlement complexity during the Roman Empire, using high-resolution stable isotope analysis. We compared infant feeding practices using published and new isotopic data from the urban sites of Pompeii (Italy) and Thessaloniki (Greece), and from the low-complexity sites of Ostia AVM (Italy) and Bainesse (UK). We found that infants in urban centers usually ceased breastfeeding by the age of 2 years or earlier, in accordance with guidelines from Roman physicians. However, in lower complexity sites cessation of breastfeeding occurred later than 2 years postpartum. This suggests a link between settlement complexity and infant feeding practices, a phenomenon mirrored in modern-day countries.

## Introduction

Cities have been described as complex systems showing well-defined properties across social, economic, and technological aspects at different scales and periods (1–3). The impact of urban complexification extends to healthcare practices well documented in contemporary societies (4, 5). Among these are infant feeding practices, with important consequences for child survival and healthy development (6–11). Recent studies have shown clear dichotomies between urban vs. rural infant feeding practices in different countries (12–16) despite established pediatric feeding guidelines (17–21) and the impact of socioeconomic or cultural aspects (22–24).

Bioarchaeological research has shown that the health and nutrition of people residing in ancient cities in the United Kingdom and Europe was overall better comparatively to rural inhabitants (25–35), a parallel also mirrored in modern societies (4). Yet, the impact of settlement complexification on infant feeding practices in historical communities remains largely unexplored. In this respect, the Roman world was home to settlements with marked differences in complexity and has a legacy that persists in Mediterranean and Western cultures until today (36, 37). Building on contemporary research that highlights connections between infant feeding practices and settlement complexity, this study explores the hypothesis that a similar connection was present in the Roman Empire.

Evidence of Roman infant feeding practices is mostly based on the study of archaeological remains and written sources. This includes the study of artifacts such as ceramic feeding bottles discovered in burial contexts or artistic portrayals of breastfeeding (38), surviving documents, such as contracts that report the hiring of wet nurses (39–41), and ancient medical treatises which gave detailed guidelines on infant feeding practices (40–44) (Supplementary Material S1). Notable examples of the latter include Soranus’ Gynaecology (early 2^nd^ century CE), Galen's Hygiene (late 2^nd^ century CE), and Oribasius’ *Collectiones Medicae* (4^th^ century CE) which extensively discuss and prescribe infant feeding practices. In these treatises, lost works from Greek and Roman physicians such as Damastes, Mnesitheus, Aristanax, and Antyllus are often cited, and occasionally criticized, offering additional insights into the subject. Overall, these treatises propose a gradual transition from exclusive breastfeeding to a wider range of foods (i.e. complementary feeding) starting at around six months and the cessation of breastfeeding by 2 years of age. However, these medical treatises primarily targeted Roman aristocracy and were prescriptive, while infant feeding practices for the broader Roman population remain largely unknown.

Direct reconstruction of past infant feeding practices at an individual temporal resolution of c. 6 months can be achieved using measurements of stable carbon (*δ*^13^C) and nitrogen (*δ*^15^N) isotope ratios on archaeological dentine increments (45–53). Permanent teeth tissues, once formed, do not undergo renewal, encapsulating isotopic signatures of the infancy and adolescent periods (51, 54, 55). First human permanent molars (M1s) record isotopic signals from c. three months postpartum to around 9.5 years of age (54), allowing for the study of early diet, including breastfeeding and complementary feeding, in individuals that have survived the most critical stages of childhood. This makes them a reliable proxy to explore infant feeding practices and circumvent the “osteological paradox’, i.e. the challenge of studying past infant health using the remains of those who did not survive infancy (56, 57). A typical pattern for the temporal sequence of isotopic values from M1 tooth increment measurements is an initial decrease, particularly visible for *δ*^15^N, which identifies the transition from exclusive human milk consumption to the introduction of complementary foods. The milk from mothers or wet nurses is ^15^N-enriched relative to their diets, and during weaning, infants are fed with food sources (e.g. cereal food preparations or fruit), which are comparatively ^15^N-depleted. The isotopic point at which the decrease in *δ*^15^N ceases, and there is a subsequent isotopic plateau, is used to mark cessation of breastfeeding.

**Fig. 1.**
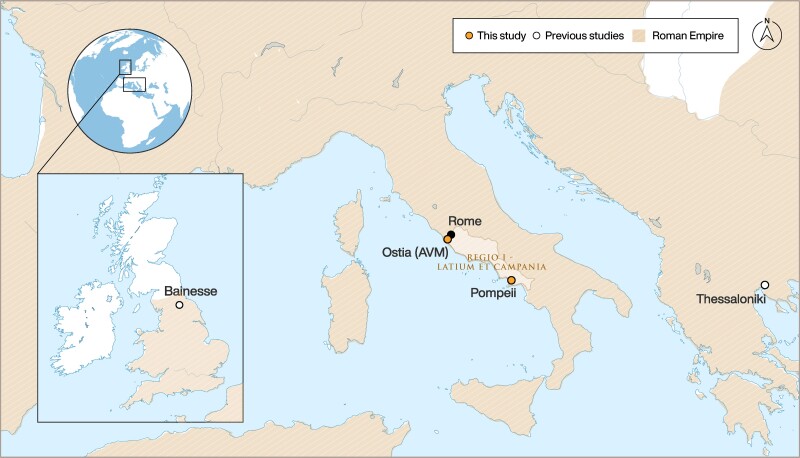
Map showing the distribution of sites analyzed in our study and from previous studies. Design by Michelle O’Reilly for MPI GEA 2024.

Incremental isotopic analysis was previously employed to investigate Roman infant feeding practices at Bainesse, UK (58, 59), a small civilian settlement located at the Empire's northern frontier (60), and in Thessaloniki (61, 62), Greece, a thriving commercial city (63, 64). To investigate potential differences in Roman infant feeding practices and test the hypothesis that these can be also linked to settlement complexification, we combined published data with new tooth dentine increment isotopic measurements for Roman individuals from the city of Pompeii (*n* = 4) and the rural area of Ostia (i.e. cemetery of Via del Mare “scavo ANAS”, from now Ostia AVM) (*n* = 6) (Fig. 1) (Supplementary Material S2). Pompeii was buried by pyroclastic material during the eruption of Mount Vesuvius in 79 CE, preserving a cross-section of everyday life in a Roman city (65–67). The archaeological site of Ostia AVM is a well-preserved repository of rural activities pertinent to the agricultural milieu of the cities of Rome and Ostia (65, 68–70). Both locations, bearing similarities in settlement complexity to Thessaloniki (61–64) and Bainesse (58, 60), respectively, allow for broader comparative analyses between the core and provincial settings of the Roman Empire for a total dataset of 45 individuals corresponding to 553 isotopic measurements.

## Results and discussion

Measurement results for carbon and nitrogen isotopic ratios and elemental concentrations (71, 72) for each dentine increment are given in Supplementary Material S3.3. Carbon and nitrogen elemental concentrations indicated that samples were well preserved, except for sample OS1 (from Ostia AVM), which was excluded from further discussion. Age intervals were assigned to each increment by accounting for variations in growth rates along the length of a tooth (see Supplementary Material S3.2). However, the same increment in different individuals may not correspond to the same age interval, preventing direct comparison of isotopic values. Hence, the Bayesian model OsteoBioR was used to convert tooth isotopic measurements across section length into isotopic ranges across time (58) (further details in Methods and results in Supplementary Material S3.4). Other previous studies of Roman infant feeding practices (73–79) relied on bone or full tooth measurements, or incremental dentine analysis of teeth reflecting different childhood years (80, 81). These were not considered here, given their lower temporal resolution (multiple years) in comparison to tooth increment studies (c. six months) (53, 57) or, in the second case, due to data gaps during the breastfeeding period.

**Fig. 2.**
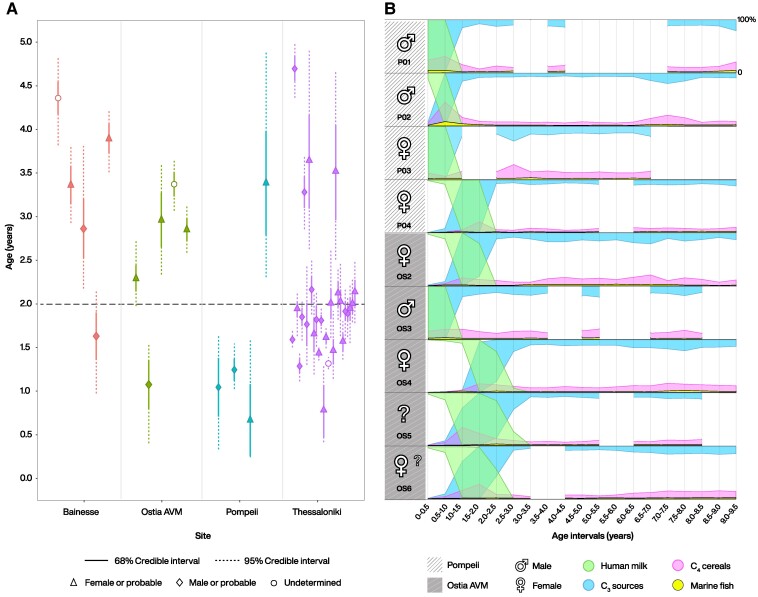
(A) Bayesian estimated 68 and 95% credibility intervals for weaning completion at Bainesse, Ostia AVM, Pompeii, and Thessaloniki. Individual OS1 from Ostia AVM is not included in the plot due to poor sample preservation. Individuals METi_197, METi_125 and METi_67 from Thessaloniki are also excluded because their credibility intervals could not be estimated. (B) Bayesian dietary estimates of primary food source protein intake for individuals from Pompeii and Ostia AVM, spanning from birth to approximately 9.5 years old. Gaps in the estimates (e.g. PO1 [3.0–3.5]) are due to excluded isotopic measurements that did not meet reliability criteria. See Supplementary Material S3 for detailed modeling methods and estimates. A larger version of Fig. 2A, complete with individual IDs, can be found in Supplementary Material S3.5. Figure design by Michelle O’Reilly for MPI GEA 2024.

Bayesian estimates of breastfeeding cessation ages were done using the ChangeR algorithm based on the MCP package for point change detection (see Material and methods) (Fig. 2A). We considered as a reference for comparison a recommended cessation of breastfeeding age of 2-years as suggested by modern-day and Roman physicians (17–21, 40–44). For individuals METi_197, METi_125, and METi_67 from Thessaloniki, the ChangeR algorithm was unable to estimate breastfeeding cessation. This is due to the absence of a significant descending *δ*^15^N trend in these individuals. According to Ganiatsou and colleagues (61), this lack of trend may imply the absence of breastfeeding. While this is a possible explanation, we argue that the likelihood of survival without breastfeeding at that time would have been exceedingly low (82). Instead, we argue that such a *δ*^15^N pattern may indicate significant dietary changes in the mother or wet-nurse, which subsequently shifted *δ*^15^N values in human milk. For the remaining dataset, estimates of cessation of breastfeeding vary from approximately 18 months to nearly 5 years. At both the sites of Bainesse and Ostia AVM, 80% of the individuals (four out of five in Bainesse and Ostia AVM) have 68 and 95% credible intervals for estimates of breastfeeding cessation above the 2-year threshold. Conversely, at the urban site of Pompeii, 75% (three out of four) of the individuals fall with their 68 and 95% credible intervals below the 2-year threshold. In the city of Thessaloniki, the 68 and 95% credible intervals for 78% (21 out of 27) of individuals are concentrated around or below the 2-year threshold. There are no clear differences in breastfeeding cessation ages according to biological sex.

For Pompeii and Ostia AVM, it was possible to establish an isotopic baseline for likely consumed food groups (83, 84). Using the Bayesian mixing model ReSources (85–89) (results and modelling description given in Supplementary Material S3.6), we generated individual temporal estimates of the caloric contributions from different protein sources (Fig. 2B). Bayesian dietary estimates suggest that human milk was gradually replaced by terrestrial C_3_ sources, including cereals (such as wheat, barley, and rye), legumes, vegetables, and secondary products were the primary protein sources for children in these communities. The consumption of C_4_ cereals, likely millet and sorghum, is also moderately estimated. While there is evidence of the usage of these cereals in Roman Italy, including direct evidence from Pompeii, they were not the most favored cereals among Romans (90, 91). The estimates also indicate minimal consumption of marine protein, which is particularly surprising for Pompeii given its coastal location and significance in the trade routes of the Gulf of Naples (67, 92, 93). However, this finding aligns with earlier Roman isotopic studies that have identified a predominant consumption of marine food by adults, in contrast to infants (79, 94). In Roman society, infants were often considered lower status individuals (58, 95) and due to the high economic value of marine fish (96) these foods may have been less accessible to them.

Studies of modern-day urbanization processes reveal the benefits and risks associated with the rapid expansion of cities (4, 97–100). The greater organizational complexity of urban settlements and their higher availability of resources, healthcare facilities, and social services positively impact public health overall (4). Cities also serve as information hubs, promoting better adherence to medical guidelines compared with rural settlements, where knowledge is typically shared within household networks and traditional practices may prevail (101, 102). This pattern extends to infant feeding practices. Substandard levels of medical information and healthcare facilities in rural areas have been linked to a lower adherence to medical recommendation of breastfeeding practices (14–16). Infant feeding practices are also impacted by wealth inequality. In impoverished families, breastfeeding is often utilized as a means to alleviate resource scarcity and reduce the financial burden of purchasing additional food for children (23). Whereas wealthier families could afford human milk substitutes and present shorter breastfeeding trends (23).

Contemporary infant feeding practices are significantly impacted by the dissemination of information and medical infrastructures and socioeconomic differences. These factors are often interlinked and associated with settlement complexity. Differences in status or adherence to diverse traditional practices among migrants might explain the limited intra-site variability observed in breastfeeding cessation within our findings (Fig. 2A). While our dataset does not include information on socioeconomic status, it is reasonable to expect higher levels of socioeconomic inequality and human spatial mobility within well connected, complex urban centers like Pompeii and Thessaloniki (63–67). In Pompeii, the 79 CE eruption of Mount Vesuvius preserved the remains of enslaved individuals, freedmen, foreigners, and citizens in the same archaeological strata, often complicating the attribution of social status and cultural affiliations. In Thessaloniki, part of the burials is possibly associated with the local middle class (61). Differences in status and spatial mobility are also present in less complex settlements such as Ostia AVM, where inscriptions reveal Greek names of freedmen and a few sarcophagi alongside wealthier incineration graves (69, 70). However, the individuals in our dataset were discovered in simple earthen pits with no grave goods, indicating low social status. This is the most common burial practice in the Ostia AVM cemetery, suggesting that the sampled individuals were likely part of the poorer agricultural segment of the population. Bainesse is characterized as a low-complexity settlement due to its small size and position at the margins of the Empire. It was a civilian settlement set close to a military fort on the Roman northern border, primarily focusing on local crafting activities and trade (59, 60). Hence, a potential presence of traders and soldiers from other parts of the empire, potentially indicating intra-site socioeconomic differences cannot be ruled out.

The difference in estimates of breastfeeding cessation is, however, clear between low- (Bainesse and Ostia AVM) and high-complexity (Pompeii and Thessaloniki) settlements. Cultural differences may partially account for these variations. For example, at Bainesse in northern Britain, it is possible that local Iron Age customs persisted throughout the Roman period, although the lack of high-resolution isotopic data for comparative analysis poses a challenge. Nevertheless, this hypothesis does not fully explain the differences observed between Pompeii and Ostia AVM, both situated within the Roman region “*Latium et Campania’*. Climatic and environmental factors are also unlikely to be the primary causes of variation since Pompeii, Ostia AVM, and Thessaloniki experience similar conditions. In Bainesse, however, harsher climatic conditions might have prompted different strategies for infant care, thereby resulting in extended breastfeeding periods. Results instead align more consistently with the proposed hypothesis of a link between infant feeding practices and settlement complexity. Contrasting lifestyles are also described in the works of classical authors such as Virgil, Cato the Elder, Varro, Horace, Columella, and Terence (103–107). Although they do not specifically discuss infant feeding practices, these authors contrast idyllic pastoral life with that of frantic urban centers.

Despite the negative features of highly complex Roman settlements, these would have greater access to medical facilities and dissemination of medical knowledge. Contemporary research has indicated that less complex settlements receive lower levels of medical support and information compared to their more developed counterparts (14–16). In these low-complexity settings, the limited availability of medical facilities often results in a reliance on traditional familial practices, leading to the vertical transmission of infant feeding knowledge that frequently diverges from medical recommendations. Available evidence on Roman medicine suggests that wealthier individuals could afford private physicians who made home visits, while the poor had to consult doctors in public baths, temples, or medical offices (*tabernae medicae*) (108, 109). Hospitals (*valetudinaria*) were available exclusively to soldiers in military encampments (110). Within this framework, it is reasonable to assume that public medical infrastructures in Bainesse and Ostia AVM were relatively limited, being primarily concentrated in the nearby military fort of Cataractonium (59) or the urban areas of Ostia and Portus (111), respectively. Conversely, Pompeii and Thessaloniki, as affluent centers of commerce and culture, likely offered more economic opportunities for doctors and a greater access to medical facilities and a higher adherence to medical recommendations to inhabitants. The discovery of numerous surgical instruments in Pompeii's “House of the Surgeon” in 1770 notably highlights the advanced state of medical practice in this urban center (112).

The analysis of our compiled dataset is consistent with the hypothesis linking settlement complexity and infant feeding practices in the Roman world. Settlement complexity is linked to levels of socioeconomic inequality, degrees of human spatial mobility, and accessibility to medical knowledge and infrastructures that likely impacted breastfeeding cessation rates. We note, however, that the dataset should be expanded in the future to test the robustness of our results and explore the impact of cultural or environmental factors. It may not always be possible to employ high temporal resolution isotopic techniques given poor sample preservation or past choices of funerary practices (e.g. cremation) (113, 114) and these are labor and cost intensive. However, they can offer unprecedented insights into the individual biographies of wide strata of past populations.

## Materials and methods

Incremental dentine stable isotope analysis on human permanent first molars is used to reconstruct infant feeding practices from c. three months to around the age of 9.5 years (54) circumventing the “osteological paradox’ (56, 57). In addition to compiling previously published isotopic data from Roman sites, we performed incremental dentine stable carbon and nitrogen isotope analyses on the first permanent molars of human remains from Roman Pompeii (n = 4) and Ostia Via del Mare (AVM) (*n* = 6). The research was conducted with the approval and cooperation of the Parco Archeologico di Pompei and the Parco Archeologico di Ostia Antica. Our tooth selection criteria included a visual evaluation of preservation status and the lack of dental wear. The biological sex and estimated ages at death for the individuals sampled were determined using established osteological techniques, with details provided in Supplementary Material S2.

The individuals from Pompeii, whose remains are curated by the Laboratorio di Ricerche Applicate of the Parco Archeologico di Pompei, are victims of the Mt. Vesuvius eruption in 79 CE. The remains from the Ostia AVM cemetery are curated by the Servizio di Antropologia of the Parco Archeologico di Ostia Antica. These individuals are part of a broader skeletal collection that was discovered in the 1990s during construction works in the periphery of Rome, in proximity of Acilia and Dragona (69, 70). The excavation of this cemetery, which dates back to the 1^st^-2^nd^ centuries CE, revealed over 300 graves. These were mostly individuals of lower social status, who were likely engaged in agricultural activities in villas belonging to Roman landowners.

Tooth enamel from sampled molars was removed using a Dremel drill and saved for potential future research on spatial mobility. Molars were then cut in half using a Buehler Isomet Low-Speed Precision Cutter at the Department of Environmental Biology, Sapienza University of Rome. Tooth halves underwent pretreatment and incremental sectioning in accordance with Beaumont et al.'s method 2 (115) at the “iCONa” lab at the Università degli studi della Campania “Luigi Vanvitelli” in Caserta, Italy, which is also where isotope measurements are produced. For a detailed explanation of sample pretreatment and analysis, refer to Supplementary Material S3.1. Comparative data for the sites of Bainesse (UK) (58) and Thessaloniki (Greece) (61, 62) were standardized and are made available via the Amalthea database (116, 117), a global historical database of isotopic measurement of tooth increments.

Conversion of individual tooth section isotopic data relative to length units into isotopic ranges across time (6-month intervals) was made using a Bayesian software OsteoBioR model instance (for further details, see Supplementary Material S3.2-S3.3-S3.4). This facilitates direct comparison of isotopic profiles amongst multiple individuals. Mean isotopic values for each time interval were then used to determine the cessation of breastfeeding (using ChangeR algorithm described below) and generate dietary estimates for individuals from Pompeii and Ostia AVM (using the Bayesian mixing model ReSources described below and more detailed in Supplementary Material S3.6). The use of point estimates entails that our approach is not a fully integrated Bayesian approach. However, this simplification is not expected to introduce any major bias since tooth sectioning was fairly uniform, and section widths are expected to approach a 6-month time interval. The two-step modelling approach also removes potential issues with model convergence under a more complex approach.

We developed the R-based ChangeR algorithm to detect *δ*^15^N point changes across time for each individual (see Supplementary Material S3.5). This Bayesian algorithm compares alternative models consisting of a different number of isotopic sections with and without additional prior information using the R package mcp (118). In the comparison, we considered up to a maximum of four linear segments, with the first segment having an optional prior (a downward slope for *δ*^15^N). Models were ranked, at a first stage, using the R package leave-one-out cross-validation (LOO (119)) relying on a computation of Estimated Log Predictive Density (ELPD). In our application, we were interested in a clear preference for a simpler model favoring the detection of breastfeeding cessation. LOO's ability to prefer a simpler model has been previously discussed (120, 121). Thus, to achieve our goals, we introduced further selection criteria. The difference between the ELPD of the first-ranked LOO model and the remainder of the models was divided by the corresponding standard error. This ratio is similar to a *z*-score, and any model with a ratio larger than 5 was excluded. Preserved models were then ranked according to the lowest number of model parameters to favor the simplest model. We tested this approach on various real data and simulated examples and found it to yield desirable results. Point changes corresponding to breastfeeding cessation are summarized by their 68 and 95% credible intervals. ChangeR code is available at the Pandora & IsoMemo software repository (https://github.com/Pandora-IsoMemo/ChangeR).

The Bayesian dietary mixing model ReSources (formerly FRUITS) (85–89) was used to reconstruct early childhood diets from individuals from Pompeii and Ostia AVM sites. Human milk intake is considered starting from birth until the end of the estimated breastfeeding period for each individual, following a descendant trend. To build the model, we considered that there is a general agreement among both contemporary and ancient Roman physicians that the introduction of complementary foods should typically start around four to six months (17–21, 42–44). This practice is grounded on the growing nutritional needs of infants. Given high infant mortality rates and poor hygiene during the Roman period (95, 122, 123), delayed introduction of complementary foods beyond six months and up to 1 year is improbable. Descriptions of the modeling details are provided in Supplementary Material S3.6.

## Supplementary Material

pgae566_Supplementary_Data

## Data Availability

Data that supports this research are available in the Supplementary Material file. Codes for OsteoBioR, ChangeR, and ReSources models are freely available at the Pandora&IsoMemo Softwary Repository (https://github.com/Pandora-IsoMemo).
