# Supplementary material for: High-resolution isotopic data link settlement complexification to infant diets within the Roman Empire
Source: PNAS Nexus. 2025 Jan 14;4(1):pgae566. doi: 10.1093/pnasnexus/pgae566 (PMC11729708; doi:10.1093/pnasnexus/pgae566)
Supplement: pgae566_Supplementary_Data [file pgae566_supplementary_data.docx]

**
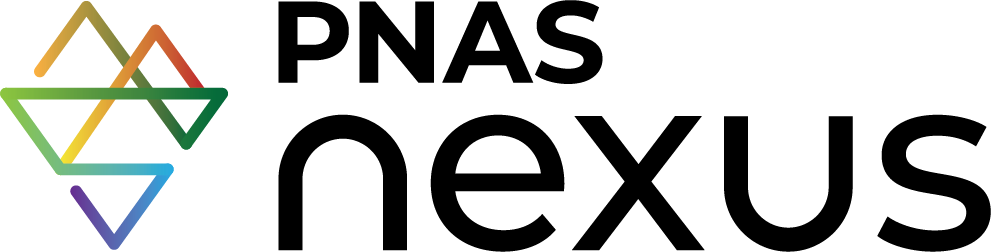
**

**High-resolution isotopic data links settlement complexification to infant diets within the Roman Empire**

Carlo Cocozza^1,2,3*^, Alison J.T. Harris^1,4^, Giulia Formichella^5^, Giulia Pedrucci^6^, Paola Francesca Rossi^7^, Alessandro D’Alessio^7^, Valeria Amoretti^8^, Gabriel Zuchtriegel^8^, Michelle O’Reilly^1^, Noemi Mantile^2^, Sofia Panella^5^, Mary Anne Tafuri^5^, Simona Altieri^2^, Maria Rosa di Cicco^2^, Ricardo Fernandes^1,9,10,11*^, Carmine Lubritto^2^.

1. Department of Archaeology, Max Planck Institute of Geoanthropology, Kahlaische Str. 10, Jena, 07745, Germany.
2. Dipartimento di Scienze e Tecnologie Ambientali Biologiche e Farmaceutiche (DiSTABiF) and Mediterranean bioArchaeological Research Advances (MAReA) centre, Università degli studi della Campania “Luigi Vanvitelli”, Via Vivaldi 43, Caserta, 81100, Italy.
3. ArchaeoBioCenter (ABC), Ludwig-Maximilians-Universität München, Geschwister-Scholl-Platz 1, München, 80539, Germany.
4. Department of Archaeology, Memorial University of Newfoundland and Labrador, St. John’s, NL A1B 3R6, Canada.
5. Dipartimento di Biologia Ambientale and Mediterranean bioArchaeological Research Advances (MAReA) centre, Sapienza University of Rome, Piazzale Aldo Moro 5, Roma, 00185, Italy.
6. Dipartimento Di Culture e Civiltà, University of Verona, Via dell’Università, 4, Verona, 37129, Italy.
7. Parco archeologico di Ostia antica, MIC Viale dei Romagnoli 717, Roma, 00119, Italy.
8. Parco Archeologico di Pompei, P.zza Porta Marina Inferiore 7, Pompei, 80045, Italy.
9. Faculty of Archaeology, University of Warsaw, Krakowskie Przedmieście 26/28, Warsaw, 00-927, Poland.
10. Arne Faculty of Arts, Masaryk University, Nováka 1, Brno-střed, 602 00, Czech Republic.
11. Climate Change and History Research Initiative, Princeton University, Princeton, NJ 08544, USA.

* Carlo Cocozza; Ricardo Fernandes.

**Email:**  [cocozza@gea.mpg.de](mailto:cocozza@gea.mpg.de); [fernandes@gea.mpg.de](mailto:fernandes@gea.mpg.de).

**This PDF file includes:**

Supplementary text S1 to S3

In text S3: figures S3.4.1 to S3.5.2

In text S2 and S3: tables S2.1 to S3.6.5

SI References

**Supplementary Information Text S1**

**Ancient Medical Sources on Roman Breastfeeding and Weaning**

By Giulia Pedrucci

Infant nutrition in ancient Rome represents a nuanced challenge for historians, due in large part to the limited available sources on the topic. Obstacles include not only a scarcity of written documents - an issue common across many areas of ancient history - but also the lack of accounts from those directly involved in the care of children, i.e. mothers and wet-nurses. Although some documents, including poetry by women from the Greek and Roman eras, do exist, they rarely touch upon their experiences with motherhood or childcare. As a result, much of what we know comes from medical texts, which primarily discuss breastfeeding and provide scant details on weaning (mainly: Sor. *Gyn*. 2.17–29; Gal. *San. Tu.* 1.7; 1.9.1; Orib. *Med. Coll. Inc.* 31–35; 37.4–5; 38.13–23)(1–3), shadowing the influence of personal choices and circumstances such as economic status and geographical location on feeding practices.

Ancient medical texts and natural scientists, from Hippocrates to Aristotle, have described human milk as "cooked blood"(4). In contrast, Empedocles (DK A 68) peculiarly referred to it as "white pus", perhaps alluding to colostrum, which was not fed to infants likely due to cultural taboos(5). It was recommended for women in ancient times to delay breastfeeding, with periods ranging from 3 to 20 days post-birth, during which new-borns might be fed honey and some goat milk(6). Galen praised breast milk as the most natural and appropriate food, while Soranus noted the potential physical stress it could cause the mother(1, 2). The process of weaning, according to Soranus and Oribasius, should begin with semi-solids foods at six months and end by the age of two. Despite various guidelines, the precise timing for ending breastfeeding varied, with most historical documents suggesting it typically lasted about two years(7, 8). Infants’ early diets should have included items like softened bread crumbs, hydromel, milk, sweetened wine, grain soup, porridge, and eggs, whereas meats and legumes had to be initially avoided(6). Soranus emphasised tailoring the introduction of solid foods to each child's strength and the onset of teething to prevent weakness(8). Additionally, Soranus challenges the recommendations of some physicians such as the Greek Mnesitheus and Aristanax, who advocate for delaying the weaning of female infants by six months(1). In his perspective, such a gender-based approach is flawed, as there are instances where male infants exhibit greater fragility than their female counterparts. Moreover, he asserts that until the baby develops teeth, a regimen consisting solely of milk is warranted.

Interestingly, in ancient medical sources discussions about infant feeding rarely ventured beyond human milk. Soranus (Sor. 2.17)(1) also mention reintroducing breastfeeding during illness, a practice supported by Caelius Aurelianus for epilepsy (*Maladies chroniques* III, 8, 147)(9). Furthermore, Pliny (e.g. *HN* 11.239; 28.257)(10) mentioned donkey and goat milk for aiding with bowel issues and teething, respectively, and recommended butter for teething pain. However, despite its therapeutic uses, animal milk was often viewed with suspicion, believed to cause health problems like abscesses, boils, rashes, and kidney stones, leading to caution in its consumption(11, 12).

**Supplementary Information Text S2**

**Description of Sampled Populations and Anthropological Methods**

Four human permanent first molars (M1) were obtained from the Roman site of Pompeii under a collaboration with the Laboratorio di Ricerche Applicate at the Parco Archeologico di Pompei. These samples belonged to cranial remains that were part of a vast collection of skeletal findings in the city, and associated with the catastrophic eruption of Mt. Vesuvius in 79 CE. Initially stored at the Terme Femminili in Pompeii, these osteological specimens were later transferred to their current repository at the Parco Archeologico di Pompei during the 20^th^ century, resulting in the loss of much of their original contextual information. Despite this, the cranial samples allowed for the determination of biological sex, exhibiting pronounced hyperfeminine and hypermasculine morphological traits(13–15).

Additionally, six individuals were sampled from the Parco Archeologico di Ostia. These were from the rural areas of the Roman city and were uncovered during construction activities in the 1990s by the Italian highways company (ANAS) in Via del Mare(16, 17) - the principal road linking both the ancient and modern cities of Rome to Ostia. Over 300 graves were unearthed in this cemetery, featuring both burial and cremation rites, and dating back to the 1^st^-2^nd^ centuries CE. These individuals predominantly represented lower social strata, often engaged in agricultural activities in landowners’ villas disseminated in the rural landscape of Ostia and Rome. Age at death estimates were done using various methods including analysis of dental wear(18), changes in the pubic symphysis(19, 20), sternal and rib joint fusions(21), and dental development in younger individuals(22, 23). The assessment of biological sex was restricted to adult specimens and based on the examination of sexual dimorphism in skeletal attributes(24–28). Since 2017, these individuals are part of a broader anthropological investigation aimed at investigating demographic trends, pathologies, migration patterns, nutritional statuses, and genetic backgrounds of the population in ancient Ostia(29).

Table S2.1 provides a summary of anthropological data relevant to the individuals analysed in this study. Whereas Table S2.2 reports anthropological information for individuals found in Roman Thessaloniki(30, 31) (n=20, 2^nd^ C. BCE - 4th C. CE), Greece, and Bainesse(32) (n=5, 3^rd^-4^th^ C. CE), UK, compiled for this study.

***Table S2.1.*** *Information on sampled individuals and associated teeth from Pompei and Ostia AVM.*

| **Burial ID** | **Isotopic ID** | **Biological Sex** | **Age at Death** | **Sampled Tooth** | **Tooth Longitudinal Length** | **Site** |
| --- | --- | --- | --- | --- | --- | --- |
| T10 | PO1 | M | Adult | Mandibular M1 | ~17mm | Pompeii |
| TF003 | PO2 | M | Adult | Mandibular M1 | ~17mm | Pompeii |
| - | PO3 | F | Adult | Mandibular M1 | ~16mm | Pompeii |
| - | PO4 | F | Adult | Mandibular M1 | ~16mm | Pompeii |
| AVM T225 | OS1 | M | 20-30 (yrs) | Mandibular M1 | ~17mm | Ostia, Via del Mare |
| AVM T247 | OS2 | F | 24-30 (yrs) | Maxillary M1 | ~15mm | Ostia, Via del Mare |
| AVM T277 | OS3 | M | 25-35 (yrs) | Maxillary M1 | ~15mm | Ostia, Via del Mare |
| AVM T302 | OS4 | F | 24-30 (yrs) | Maxillary M1 | ~18mm | Ostia, Via del Mare |
| AVM T242 | OS5 | - | 16-17 (yrs) | Mandibular M1 | ~18mm | Ostia, Via del Mare |
| AVM T248 | OS6 | ?F | 15-20 (yrs) | Maxillary M1 | ~17mm | Ostia, Via del Mare |

***Table S2.2.*** *Information on compiled individuals and associated teeth from Thessaloniki*(30, 31) *and Bainesse*(32)*.*

| **Burial ID** | **Isotopic**  **ID** | **Biological**  **Sex** | **Age at Death** | **Sampled**  **Tooth** | **Tooth Longitudinal Length** | **Site** |
| --- | --- | --- | --- | --- | --- | --- |
| 52 | METi_257 | M | 24-30 (yrs) | Maxillary M1 | ~16mm | Thessaloniki |
| 253 | METi_247 | F | 40-50 (yrs) | Maxillary M1 | ~19mm | Thessaloniki |
| 253 | METi_245 | F | 40-50 (yrs) | Maxillary M1 | ~16mm | Thessaloniki |
| 79 | METi_239 | F | 20-25 (yrs) | Maxillary M1 | ~13mm | Thessaloniki |
| 213 | METi_237 | ?F | Adult | M1 | ~15mm | Thessaloniki |
| 376 | METi_223 | F | 44-55 (yrs) | Maxillary M1 | ~20mm | Thessaloniki |
| 859 | METi_221 | F | Adult | Maxillary M1 | ~16mm | Thessaloniki |
| 9 | METi_213 | - | - | Maxillary M1 | ~15mm | Thessaloniki |
| 1900 | METi_207 | F | Adult | Maxillary M1 | ~16mm | Thessaloniki |
| 33 | METi_203 | F | 45-55 (yrs) | Maxillary M1 | ~15mm | Thessaloniki |
| 1751 | METi_197 | M | 20-30 (yrs) | Maxillary M1 | ~15mm | Thessaloniki |
| 692 | METi_195 | M | 40-55 (yrs) | Maxillary M1 | ~18mm | Thessaloniki |
| 59 | METi_193 | F | Adult | Maxillary M1 | ~18mm | Thessaloniki |
| 329 | METi_191 | ?M | 21-25 (yrs) | Maxillary M1 | ~20mm | Thessaloniki |
| 1920 | METi_189 | F | Adult | Maxillary M1 | ~17mm | Thessaloniki |
| 1900 | METi_187 | F | Adult | Maxillary M1 | ~16 | Thessaloniki |
| 363 | METi_169 | ?F | 20-36 (yrs) | Maxillary M1 | ~15mm | Thessaloniki |
| 672 | METi_163 | M | 15-21 (yrs) | Maxillary M1 | ~19mm | Thessaloniki |
| 690a | METi_157 | M | 20-35 (yrs) | Maxillary M1 | ~20mm | Thessaloniki |
| 690b | METi_144 | M | 35-40 (yrs) | Maxillary M1 | ~16mm | Thessaloniki |
| 251 | METi_141 | M | 18-25 (yrs) | Maxillary M1 | ~18mm | Thessaloniki |
| 244 | METi_127 | F | 50+ (yrs) | Maxillary M1 | ~18mm | Thessaloniki |
| 241 | METi_125 | M | 35-50 (yrs) | Maxillary M1 | ~15mm | Thessaloniki |
| 42 | METi_119 | ?M | 21-25 (yrs) | Maxillary M1 | ~14mm | Thessaloniki |
| 8 | METi_115 | M | 35-50 (yrs) | Maxillary M1 | ~16mm | Thessaloniki |
| 1427 | METi_99 | F | 25-35 (yrs) | Maxillary M1 | ~15mm | Thessaloniki |
| 1884 | METi_97 | F | Adult | Maxillary M1 | ~17mm | Thessaloniki |
| 713 | METi_83 | F | 24-30 (yrs) | Maxillary M1 | ~14mm | Thessaloniki |
| 210 | METi_71 | M | 25-35 (yrs) | Maxillary M1 | ~15mm | Thessaloniki |
| 553 | METi_67 | M | 18-22 (yrs) | Maxillary M1 | ~15mm | Thessaloniki |
| Sk13168 | BN15 | M | 18-25 (yrs) | Maxillary M1 | ~15mm | Bainesse |
| Sk12561 | BN124 | - | 15-16 (yrs) | Maxillary M1 | ~21mm | Bainesse |
| Sk12860 | BN144 | ?F | 26-35 (yrs) | Maxillary M1 | ~16mm | Bainesse |
| Sk13146 | BN197a | M | 36+ (yrs) | Maxillary M1 | ~18mm | Bainesse |
| Sk13340 | BN213 | F | 36-45 (yrs) | Maxillary M1 | ~15mm | Bainesse |

**Supplementary Information Text S3**

**Description of Analytical Methods, Modelling, and Results**

***S3.1 Stable Isotope Analysis Protocol for Pompeii and Ostia AVM samples***

For each molar from Pompeii and Ostia AVM, tooth enamel was removed using a Dremmel Drill and saved for potential future analysis on spatial mobility. Samples were then cut in half using a Buehler Isomet Low-Speed Precision Cutter at the Department of Environmental Biology, Sapienza University of Rome, Italy. Each tooth half was then immersed in 8mL of 0.5M HCl acid and stored at 4°C for circa three to five weeks during which the acid was replaced every two days. Once the demineralisation process was complete, samples were rinsed in distilled water three times and cut into 1 mm wide horizontal sections, from the cusp to the apex of the tooth. Each section was covered by 8 ml of pH = 3 water and gelatinised at 70°C for 48 hours. Gelatinised collagen was not filtered to maximise its yield and the samples were freeze dried. Collagen samples were weighed (0.8-1.1 mg) into tin capsules and measured at the 'iCONa' lab at the Università degli studi della Campania 'Luigi Vanvitelli' in Caserta, Italy. The measurements of carbon and nitrogen isotopic ratios were performed using an Elemental Analyser (Flash EA 1112 series, Thermo Scientific) coupled to an Isotopic Ratio Mass Spectrometer (Delta V Advantage, Thermo Scientific), in continuous flow mode (by means of a Conflo IV, Thermo Scientific). International standards were used to calibrate raw measurements, i.e. IAEA-N2 (*δ*^15^N= 20.3±0.2‰), IAEA-CH3 (*δ*^13^C= -24.724±0.041‰) together with an in-house Sirfer Yeast standard (*δ*^15^N= -1.24±0.09‰, *δ*^13^C= -20.02±0.09‰). The typical analytical precision, evaluated from repeated measurements of the standard, was 0.1‰ for *δ*^13^C and 0.2‰ for *δ*^15^N. Isotopic results are reported using delta notation relative to the international standards VPDB and AIR for carbon (*δ*^13^C) and nitrogen (*δ*^15^N) stable isotopes, respectively.

***S3.2 Age Assignment Model***

Isotopic studies have employed diverse techniques to estimate age ranges or medians within the dentine layers of archaeological human teeth(33–37). Scharlotta et al.(36) summarised these methods, identifying two primary approaches: the first uses a standard growth rate for each type of tooth(33), albeit with differing rates for crowns and roots; while the second approach calculates a sample-specific growth rate depending on its length and specific formation period according to tooth type(34). Our study implements a variation of this second approach. Acknowledging the variability in tooth formation periods across different populations, both historical and modern, we used as reference the work by Cocozza and Fernandes(38). They compiled a dataset of formation periods for human permanent first molars (M1), incisors (I1, I2), and canines (C), calculating weighted mean ages for these teeth. Additionally, we accounted for variance in growth rates along the length of the tooth, following Dean(39). To estimate age ranges for each segment, we determined a uniform growth rate by dividing measured tooth length by formation time, which we then divided into five segments using the following ratio: 0.75:0.8:1.1:1.15:1.05. This proportion is ‘variant method 11’ proposed in Scharlotta et al.(36) to align multiple molars. This variant was selected because it most closely aligns with the evolutionistic growth curve outlined by Dean and Cole(40).

***S3.3 Stable carbon and nitrogen isotope incremental analysis results and estimated ages following methods delineated in section S3.2.***

***Table S3.3.1****. Newly-generated stable carbon and nitrogen isotope incremental analysis data for Pompeii (code PO) and Ostia AVM (code OS). Increments marked with a grey background were excluded from modelling and interpretation. “n.a.” stands for “not available” as dentine increment did not produce any isotopic measurement.*

| **Isotopic ID** | **Sample ID** | **Estimated min. age (years)** | **Estimated max. age (years)** | ***δ*^13^C**  **(‰ - VPDB)** | ***δ*^15^N**  **(‰ - AIR)** | **%C** | **%N** | **Atomic**  **C/N**  **ratio** |
| --- | --- | --- | --- | --- | --- | --- | --- | --- |
| PO1 | PO1 A | 0.2 | 0.6 | -18.2 | 11.7 | 39.2 | 14.0 | 3.3 |
| PO1 | PO1 B | 0.6 | 1.0 | -18.8 | 11.1 | 41.6 | 14.1 | 3.4 |
| PO1 | PO1 C | 1.0 | 1.4 | -19.3 | 10.0 | 37.7 | 13.8 | 3.2 |
| PO1 | PO1 D | 1.4 | 1.9 | n.a. | n.a. | n.a. | n.a. | n.a. |
| PO1 | PO1 E | 1.9 | 2.3 | -19.1 | 10.5 | 39.8 | 14.0 | 3.3 |
| PO1 | PO1 F | 2.3 | 2.7 | -18.9 | 10.6 | 38.2 | 13.3 | 3.4 |
| PO1 | PO1 G | 2.7 | 3.3 | -19.0 | 8.4 | 38.9 | 9.8 | 4.6 |
| PO1 | PO1 H | 3.3 | 3.9 | n.a. | n.a. | n.a. | n.a. | n.a. |
| PO1 | PO1 I | 3.9 | 4.5 | -18.9 | 10.9 | 40.4 | 14.1 | 3.4 |
| PO1 | PO1 J | 4.5 | 5.1 | n.a. | n.a. | n.a. | n.a. | n.a. |
| PO1 | PO1 K | 5.1 | 6.0 | n.a. | n.a. | n.a. | n.a. | n.a. |
| PO1 | PO1 L | 6.0 | 7.0 | n.a. | n.a. | n.a. | n.a. | n.a. |
| PO1 | PO1 M | 7.0 | 7.8 | -19.2 | 11.4 | 39.6 | 13.3 | 3.5 |
| PO1 | PO1 N | 7.8 | 8.7 | -19.1 | 10.6 | 39.2 | 13.4 | 3.4 |
| PO1 | PO1 O | 8.7 | 9.5 | -18.5 | 10.5 | 41.3 | 13.3 | 3.6 |
| PO2 | PO2 A | 0.3 | 0.7 | -17.7 | 12.9 | 39.7 | 13.7 | 3.4 |
| PO2 | PO2 B | 0.7 | 1.1 | -18.0 | 9.9 | 42.3 | 14.9 | 3.3 |
| PO2 | PO2 C | 1.1 | 1.6 | -18.8 | 9.7 | 40.9 | 14.4 | 3.3 |
| PO2 | PO2 D | 1.6 | 2.0 | n.a. | n.a. | n.a. | n.a. | n.a. |
| PO2 | PO2 E | 2.0 | 2.5 | -19.0 | 10.4 | 39.5 | 13.7 | 3.4 |
| PO2 | PO2 F | 2.5 | 2.9 | -19.1 | 9.9 | 40.0 | 13.7 | 3.4 |
| PO2 | PO2 G | 2.9 | 3.5 | -19.0 | 10.2 | 40.7 | 14.5 | 3.3 |
| PO2 | PO2 H | 3.5 | 4.4 | -19.1 | 9.7 | 38.8 | 13.4 | 3.4 |
| PO2 | PO2 I | 4.4 | 5.1 | -19.1 | 9.7 | 39.7 | 13.5 | 3.4 |
| PO2 | PO2 J | 5.1 | 5.7 | -18.9 | 10.3 | 39.3 | 14.0 | 3.3 |
| PO2 | PO2 K | 5.7 | 6.3 | -19.2 | 10.0 | 41.6 | 13.8 | 3.5 |
| PO2 | PO2 L | 6.3 | 7.0 | -18.7 | 10.7 | 40.5 | 14.2 | 3.3 |
| PO2 | PO2 M | 7.0 | 7.6 | -18.8 | 11.1 | 38.3 | 13.3 | 3.3 |
| PO2 | PO2 N | 7.6 | 8.2 | -19.1 | 10.5 | 41.1 | 13.7 | 3.5 |
| PO2 | PO2 O | 8.2 | 9.1 | -19.0 | 10.7 | 39.5 | 13.4 | 3.4 |
| PO2 | PO2 P | 9.1 | 9.5 | n.a. | n.a. | n.a. | n.a. | n.a. |
| PO3 | PO3 A | 0.2 | 0.6 | -19.0 | 11.9 | 41.4 | 13.5 | 3.6 |
| PO3 | PO3 B | 0.6 | 1.1 | n.a. | n.a. | n.a. | n.a. | n.a. |
| PO3 | PO3 C | 1.1 | 1.5 | -19.2 | 11.0 | 42.1 | 15.4 | 3.2 |
| PO3 | PO3 D | 1.5 | 2.0 | n.a. | n.a. | n.a. | n.a. | n.a. |
| PO3 | PO3 E | 2.0 | 2.5 | -19.2 | 11.2 | 39.7 | 13.8 | 3.4 |
| PO3 | PO3 F | 2.5 | 3.0 | -18.5 | 10.3 | 41.4 | 13.3 | 3.6 |
| PO3 | PO3 G | 3.0 | 3.6 | -18.8 | 12.1 | 41.5 | 14.6 | 3.3 |
| PO3 | PO3 H | 3.6 | 4.6 | -18.8 | 11.8 | 41.8 | 14.8 | 3.3 |
| PO3 | PO3 I | 4.6 | 5.6 | -18.9 | 11.3 | 41.9 | 13.9 | 3.5 |
| PO3 | PO3 J | 5.6 | 6.6 | -18.8 | 11.9 | 42.0 | 14.7 | 3.3 |
| PO3 | PO3 K | 6.6 | 7.6 | -19.6 | -1.3 | 31.8 | 38.5 | 1.0 |
| PO3 | PO3 L | 7.6 | 8.5 | -19.7 | 10.5 | 40.8 | 12.0 | 4.0 |
| PO3 | PO3 M | 8.5 | 9.4 | n.a. | n.a. | n.a. | n.a. | n.a. |
| PO4 | PO4 A | 0.2 | 0.6 | -17.4 | 12.3 | 40.5 | 16.0 | 2.9 |
| PO4 | PO4 B | 0.6 | 1.1 | -18.2 | 13.8 | 41.7 | 14.8 | 3.3 |
| PO4 | PO4 C | 1.1 | 1.5 | -18.5 | 13.4 | 42.3 | 14.6 | 3.4 |
| PO4 | PO4 D | 1.5 | 2.0 | -19.1 | 11.7 | 42.3 | 14.9 | 3.3 |
| PO4 | PO4 E | 2.0 | 2.4 | -19.3 | 10.8 | 41.5 | 14.6 | 3.3 |
| PO4 | PO4 F | 2.4 | 3.1 | -19.2 | 10.1 | 41.6 | 14.8 | 3.3 |
| PO4 | PO4 G | 3.1 | 3.7 | -18.9 | 10.0 | 44.7 | 15.9 | 3.3 |
| PO4 | PO4 H | 3.7 | 4.3 | -19.1 | 10.3 | 40.4 | 13.8 | 3.4 |
| PO4 | PO4 I | 4.3 | 5.0 | -18.9 | 9.5 | 41.2 | 14.4 | 3.3 |
| PO4 | PO4 J | 5.0 | 5.7 | -19.1 | 8.9 | 42.4 | 12.2 | 4.1 |
| PO4 | PO4 K | 5.7 | 6.6 | -18.9 | 9.7 | 41.3 | 14.4 | 3.4 |
| PO4 | PO4 L | 6.6 | 7.5 | -18.7 | 9.9 | 42.2 | 14.9 | 3.3 |
| PO4 | PO4 M+N | 7.5 | 9.3 | -18.8 | 10.1 | 41.6 | 14.6 | 3.3 |
| OS1 | OS1 A | 0.2 | 0.6 | -19.0 | 5.6 | 4.0 | 0.9 | 5.2 |
| OS1 | OS1 B | 0.6 | 1.0 | -19.3 | 6.5 | 8.1 | 2.0 | 4.8 |
| OS1 | OS1 C | 1.0 | 1.5 | -19.8 | 4.5 | 7.2 | 1.8 | 4.6 |
| OS1 | OS1 D | 1.5 | 1.9 | -21.1 | 7.3 | 16.2 | 4.5 | 4.2 |
| OS1 | OS1 E | 1.9 | 2.4 | -19.6 | 7.3 | 15.0 | 5.6 | 3.1 |
| OS1 | OS1 F | 2.4 | 2.8 | -20.1 | -4.6 | 5.1 | -1.2 | -4.9 |
| OS1 | OS1 G | 2.8 | 3.4 | -19.7 | 5.4 | 14.5 | 4.7 | 3.6 |
| OS1 | OS1 H | 3.4 | 4.0 | -19.7 | 6.0 | 22.5 | 7.8 | 3.4 |
| OS1 | OS1 I | 4.0 | 4.7 | n.a. | n.a. | n.a. | n.a. | n.a. |
| OS1 | OS1 J | 4.7 | 5.3 | -19.2 | 6.4 | 35.5 | 12.4 | 3.3 |
| OS1 | OS1 K | 5.3 | 5.9 | n.a. | n.a. | n.a. | n.a. | n.a. |
| OS1 | OS1 L | 5.9 | 6.6 | -19.6 | 5.9 | 31.6 | 11.1 | 3.3 |
| OS1 | OS1 M | 6.6 | 7.2 | -19.6 | 5.7 | 33.7 | 12.1 | 3.3 |
| OS1 | OS1 N | 7.2 | 7.8 | n.a. | n.a. | n.a. | n.a. | n.a. |
| OS1 | OS1 O | 7.8 | 8.1 | n.a. | n.a. | n.a. | n.a. | n.a. |
| OS1 | OS1 P | 8.1 | 8.4 | n.a. | n.a. | n.a. | n.a. | n.a. |
| OS1 | OS1 Q | 8.4 | 8.7 | n.a. | n.a. | n.a. | n.a. | n.a. |
| OS1 | OS1 R | 8.7 | 9.0 | -19.2 | 5.8 | 39.1 | 14.0 | 3.3 |
| OS1 | OS1 S | 9.0 | 9.3 | -19.5 | 6.4 | 39.3 | 14.6 | 3.1 |
| OS1 | OS1 T | 9.3 | 9.5 | n.a. | n.a. | n.a. | n.a. | n.a. |
| OS2 | OS2 A | 0.3 | 0.8 | -18.6 | 12.6 | 41.5 | 15.0 | 3.2 |
| OS2 | OS2 B | 0.8 | 1.2 | -21.0 | -1.8 | 33.5 | -22.9 | -1.7 |
| OS2 | OS2 C | 1.2 | 1.7 | -18.7 | 11.6 | 38.0 | 13.2 | 3.4 |
| OS2 | OS2 D | 1.7 | 2.2 | -19.2 | 9.4 | 41.0 | 13.4 | 3.6 |
| OS2 | OS2 E | 2.2 | 2.7 | -19.6 | 10.3 | 41.6 | 14.0 | 3.5 |
| OS2 | OS2 F | 2.7 | 3.4 | -19.1 | 9.7 | 39.6 | 13.5 | 3.4 |
| OS2 | OS2 G | 3.4 | 4.0 | -18.9 | 10.5 | 39.8 | 14.2 | 3.3 |
| OS2 | OS2 H | 4.0 | 4.7 | -18.9 | 10.1 | 38.5 | 14.2 | 3.2 |
| OS2 | OS2 I | 4.7 | 5.4 | -18.9 | 9.8 | 41.7 | 14.7 | 3.3 |
| OS2 | OS2 J | 5.4 | 6.1 | -18.6 | 9.9 | 39.1 | 14.1 | 3.2 |
| OS2 | OS2 K | 6.1 | 6.8 | -19.1 | 7.5 | 41.0 | 11.2 | 4.3 |
| OS2 | OS2 L | 6.8 | 7.5 | -18.8 | 10.2 | 40.4 | 14.2 | 3.3 |
| OS2 | OS2 M | 7.5 | 8.1 | -19.0 | 10.0 | 41.5 | 14.6 | 3.3 |
| OS2 | OS2 N | 8.1 | 8.8 | -19.3 | 10.5 | 41.1 | 14.3 | 3.4 |
| OS2 | OS2 O | 8.8 | 9.4 | -19.4 | 10.4 | 41.4 | 14.3 | 3.4 |
| OS3 | OS3 A | 0.3 | 0.8 | -18.5 | 12.7 | 40.1 | 15.0 | 3.1 |
| OS3 | OS3 B | 0.8 | 1.2 | -18.7 | 11.4 | 40.1 | 14.3 | 3.3 |
| OS3 | OS3 C | 1.2 | 1.7 | -18.9 | 11.6 | 38.1 | 14.0 | 3.2 |
| OS3 | OS3 D | 1.7 | 2.2 | -19.0 | 11.5 | 39.7 | 14.4 | 3.2 |
| OS3 | OS3 E | 2.2 | 2.7 | -18.9 | 11.5 | 40.6 | 14.6 | 3.3 |
| OS3 | OS3 F | 2.7 | 3.4 | n.a. | n.a. | n.a. | n.a. | n.a. |
| OS3 | OS3 G | 3.4 | 4.0 | -18.9 | 11.7 | 41.3 | 14.1 | 3.4 |
| OS3 | OS3 H | 4.0 | 4.7 | -18.6 | 10.2 | 39.6 | 12.6 | 3.7 |
| OS3 | OS3 I | 4.7 | 5.4 | -18.7 | 12.0 | 39.9 | 14.2 | 3.3 |
| OS3 | OS3 J | 5.4 | 6.1 | -19.2 | 11.1 | 41.8 | 12.2 | 4.0 |
| OS3 | OS3 K | 6.1 | 6.8 | -19.7 | 10.6 | 42.3 | 12.6 | 3.9 |
| OS3 | OS3 L+M | 6.8 | 8.1 | -19.1 | 11.7 | 42.0 | 15.2 | 3.2 |
| OS4 | OS4 A | 0.3 | 0.7 | -17.6 | 12.9 | 40.5 | 14.1 | 3.4 |
| OS4 | OS4 B | 0.7 | 1.1 | -17.8 | 13.7 | 44.5 | 16.0 | 3.2 |
| OS4 | OS4 C | 1.1 | 1.5 | -17.5 | 13.6 | 41.0 | 14.8 | 3.2 |
| OS4 | OS4 D | 1.5 | 1.9 | -17.9 | 12.9 | 41.5 | 14.8 | 3.3 |
| OS4 | OS4 E | 1.9 | 2.3 | -18.0 | 11.1 | 40.1 | 14.3 | 3.3 |
| OS4 | OS4 F | 2.3 | 2.7 | -18.3 | 10.3 | 41.6 | 14.7 | 3.3 |
| OS4 | OS4 G | 2.7 | 3.1 | -18.4 | 10.4 | 41.2 | 14.8 | 3.2 |
| OS4 | OS4 H | 3.1 | 3.6 | -18.6 | 9.2 | 37.1 | 12.0 | 3.6 |
| OS4 | OS4 I | 3.6 | 4.2 | -18.5 | 10.5 | 41.2 | 14.8 | 3.2 |
| OS4 | OS4 J | 4.2 | 4.8 | -18.7 | 10.8 | 40.7 | 14.5 | 3.3 |
| OS4 | OS4 K | 4.8 | 5.4 | -18.6 | 10.8 | 39.0 | 14.1 | 3.2 |
| OS4 | OS4 L | 5.4 | 6.2 | -18.6 | 10.5 | 42.2 | 14.1 | 3.5 |
| OS4 | OS4 M | 6.2 | 7.0 | -18.4 | 10.7 | 37.3 | 13.3 | 3.3 |
| OS4 | OS4 N | 7.0 | 7.9 | -18.2 | 10.8 | 31.5 | 11.1 | 3.3 |
| OS4 | OS4 O | 7.9 | 8.7 | -18.2 | 10.6 | 40.8 | 14.5 | 3.3 |
| OS4 | OS4 P | 8.7 | 9.5 | -18.3 | 10.5 | 42.4 | 14.6 | 3.4 |
| OS5 | OS5 A | 0.2 | 0.6 | -18.9 | 10.3 | 42.2 | 14.8 | 3.3 |
| OS5 | OS5 B | 0.6 | 1.0 | -19.3 | 9.7 | 42.4 | 14.9 | 3.3 |
| OS5 | OS5 C | 1.0 | 1.4 | -19.2 | 9.4 | 41.4 | 15.4 | 3.1 |
| OS5 | OS5 D | 1.4 | 1.8 | -19.1 | 9.9 | 42.5 | 15.6 | 3.2 |
| OS5 | OS5 E | 1.8 | 2.2 | -19.2 | 8.3 | 42.4 | 15.4 | 3.2 |
| OS5 | OS5 F | 2.2 | 2.6 | -19.1 | 8.2 | 42.5 | 15.6 | 3.2 |
| OS5 | OS5 G | 2.6 | 3.2 | -19.3 | 7.9 | 41.7 | 15.2 | 3.2 |
| OS5 | OS5 H | 3.2 | 3.7 | -19.5 | 7.1 | 41.7 | 14.2 | 3.4 |
| OS5 | OS5 I | 3.7 | 4.3 | -19.4 | 7.8 | 41.8 | 15.0 | 3.2 |
| OS5 | OS5 J | 4.3 | 4.9 | -19.4 | 7.5 | 41.0 | 13.7 | 3.5 |
| OS5 | OS5 K | 4.9 | 5.5 | -19.3 | 7.7 | 41.3 | 14.8 | 3.3 |
| OS5 | OS5 L | 5.5 | 6.1 | -19.4 | 6.9 | 40.0 | 12.7 | 3.7 |
| OS5 | OS5 M | 6.1 | 6.6 | -19.1 | 7.7 | 41.4 | 13.8 | 3.5 |
| OS5 | OS5 N | 6.6 | 7.2 | -19.6 | 7.5 | 40.2 | 11.3 | 4.2 |
| OS5 | OS5 O | 7.2 | 7.7 | -19.4 | 8.0 | 41.3 | 13.5 | 3.6 |
| OS5 | OS5 P | 7.7 | 8.3 | -19.6 | 8.2 | 42.5 | 13.8 | 3.6 |
| OS5 | OS5 Q | 8.3 | 8.8 | -19.8 | 7.6 | 40.0 | 10.8 | 4.3 |
| OS5 | OS5 R | 8.8 | 9.3 | -19.5 | 6.9 | 67.0 | 16.8 | 4.7 |
| OS6 | OS6 A | 0.3 | 0.7 | -19.1 | 10.6 | 43.6 | 16.2 | 3.1 |
| OS6 | OS6 B | 0.7 | 1.1 | -19.3 | 9.3 | 41.7 | 15.4 | 3.2 |
| OS6 | OS6 C | 1.1 | 1.6 | -19.3 | 9.8 | 41.6 | 15.3 | 3.2 |
| OS6 | OS6 D | 1.6 | 2.0 | -19.5 | 8.7 | 45.1 | 16.6 | 3.2 |
| OS6 | OS6 E | 2.0 | 2.5 | -19.8 | 7.4 | 42.3 | 16.3 | 3.0 |
| OS6 | OS6 F | 2.5 | 2.9 | -19.6 | 6.9 | 42.6 | 16.4 | 3.0 |
| OS6 | OS6 G | 2.9 | 3.5 | -19.8 | 6.8 | 38.8 | 13.4 | 3.4 |
| OS6 | OS6 H | 3.5 | 4.4 | -18.9 | 6.2 | 34.6 | 7.2 | 5.6 |
| OS6 | OS6 I | 4.4 | 5.1 | -19.7 | 7.4 | 41.1 | 14.9 | 3.2 |
| OS6 | OS6 J | 5.1 | 5.7 | -19.6 | 7.6 | 41.0 | 14.2 | 3.4 |
| OS6 | OS6 K | 5.7 | 6.3 | -19.5 | 7.8 | 41.6 | 14.5 | 3.4 |
| OS6 | OS6 L | 6.3 | 7.0 | -19.7 | 8.0 | 42.7 | 14.1 | 3.5 |
| OS6 | OS6 M | 7.0 | 7.6 | -19.5 | 8.3 | 41.9 | 14.0 | 3.5 |
| OS6 | OS6 N | 7.6 | 8.2 | -19.6 | 8.1 | 41.3 | 13.9 | 3.5 |
| OS6 | OS6 O | 8.2 | 9.1 | -19.5 | 8.4 | 45.2 | 15.3 | 3.4 |
| OS6 | OS6 P | 9.1 | 9.5 | -19.6 | 8.4 | 40.0 | 8.5 | 5.5 |

**Table S3.3.2.** Compiled stable carbon and nitrogen isotope incremental analysis data for Thessaloniki (code METi_)(30, 31) and Bainesse (code BN)(32). Increments marked with a grey background were excluded from modelling and interpretation. “n.a.” stands for “not available” as dentine increment did not produce any isotopic measurement.

| **Isotopic ID** | **Sample ID** | **Estimated min. age (years)** | **Estimated max. age (years)** | ***δ*^13^C (‰ - VPDB)** | ***δ*^15^N (‰ - AIR)** | **%C** | **%N** | **Atomic C/N ratio** |
| --- | --- | --- | --- | --- | --- | --- | --- | --- |
| METi_257 | METi_257.1 | 0.3 | 0.7 | -18.4 | 10.2 | 42.9 | 15.8 | 3.2 |
| METi_257 | METi_257.2 | 0.7 | 1.2 | -18.6 | 9.5 | 43.0 | 16.0 | 3.1 |
| METi_257 | METi_257.3 | 1.2 | 1.6 | -18.7 | 9.0 | 40.0 | 14.9 | 3.1 |
| METi_257 | METi_257.4 | 1.6 | 2.1 | -18.5 | 8.9 | 40.8 | 15.2 | 3.1 |
| METi_257 | METi_257.5 | 2.1 | 2.5 | -18.5 | 8.9 | 43.0 | 16.0 | 3.1 |
| METi_257 | METi_257.6 | 2.5 | 3.0 | -17.7 | 8.8 | 40.9 | 15.2 | 3.1 |
| METi_257 | METi_257.7 | 3.0 | 3.6 | -17.7 | 8.9 | 39.1 | 14.5 | 3.1 |
| METi_257 | METi_257.8 | 3.6 | 4.2 | -18.1 | 9.0 | 42.4 | 15.7 | 3.1 |
| METi_257 | METi_257.9 | 4.2 | 4.9 | -18.5 | 9.0 | 43.1 | 16.0 | 3.1 |
| METi_257 | METi_257.10 | 4.9 | 5.5 | -18.6 | 9.0 | 43.2 | 16.1 | 3.1 |
| METi_257 | METi_257.11 | 5.5 | 6.2 | n.a. | n.a. | n.a. | n.a. | n.a. |
| METi_257 | METi_257.12 | 6.2 | 6.9 | n.a. | n.a. | n.a. | n.a. | n.a. |
| METi_257 | METi_257.13 | 6.9 | 7.5 | n.a. | n.a. | n.a. | n.a. | n.a. |
| METi_257 | METi_257.14 | 7.5 | 8.1 | n.a. | n.a. | n.a. | n.a. | n.a. |
| METi_257 | METi_257.15 | 8.1 | 8.7 | n.a. | n.a. | n.a. | n.a. | n.a. |
| METi_257 | METi_257.16 | 8.7 | 9.3 | n.a. | n.a. | n.a. | n.a. | n.a. |
| METi_247 | METi_247.1 | 0.3 | 0.7 | -18.5 | 12.9 | 42.8 | 15.8 | 3.2 |
| METi_247 | METi_247.2 | 0.7 | 1.0 | -18.8 | 11.7 | 42.9 | 15.9 | 3.2 |
| METi_247 | METi_247.3 | 1.0 | 1.4 | -19.4 | 10.5 | 41.7 | 15.4 | 3.2 |
| METi_247 | METi_247.4 | 1.4 | 1.8 | -19.6 | 9.9 | 42.8 | 15.8 | 3.2 |
| METi_247 | METi_247.5 | 1.8 | 2.2 | -19.2 | 9.9 | 41.2 | 15.2 | 3.2 |
| METi_247 | METi_247.6 | 2.2 | 2.6 | -19.4 | 9.7 | 41.1 | 15.1 | 3.2 |
| METi_247 | METi_247.7 | 2.6 | 2.9 | -19.4 | 9.9 | 41.0 | 15.0 | 3.2 |
| METi_247 | METi_247.8 | 2.9 | 3.5 | -19.4 | 10.0 | 43.0 | 15.8 | 3.2 |
| METi_247 | METi_247.9 | 3.5 | 4.0 | -19.3 | 9.8 | 42.6 | 15.6 | 3.2 |
| METi_247 | METi_247.10 | 4.0 | 4.5 | -19.2 | 10.0 | 42.7 | 15.7 | 3.2 |
| METi_247 | METi_247.11 | 4.5 | 5.1 | -19.2 | 9.9 | 41.6 | 15.3 | 3.2 |
| METi_247 | METi_247.12 | 5.1 | 5.6 | -19.0 | 9.8 | 43.9 | 16.2 | 3.2 |
| METi_247 | METi_247.13 | 5.6 | 6.2 | -19.0 | 10.0 | 39.1 | 14.4 | 3.2 |
| METi_247 | METi_247.14 | 6.2 | 6.7 | n.a. | n.a. | n.a. | n.a. | n.a. |
| METi_247 | METi_247.15 | 6.7 | 7.3 | n.a. | n.a. | n.a. | n.a. | n.a. |
| METi_247 | METi_247.16 | 7.3 | 7.8 | n.a. | n.a. | n.a. | n.a. | n.a. |
| METi_247 | METi_247.17 | 7.8 | 8.3 | n.a. | n.a. | n.a. | n.a. | n.a. |
| METi_247 | METi_247.18 | 8.3 | 8.8 | n.a. | n.a. | n.a. | n.a. | n.a. |
| METi_247 | METi_247.19 | 8.8 | 9.3 | n.a. | n.a. | n.a. | n.a. | n.a. |
| METi_245 | METi_245.1 | 0.3 | 0.7 | -18.9 | 12.1 | 45.7 | 16.9 | 3.1 |
| METi_245 | METi_245.2 | 0.7 | 1.2 | -19.2 | 11.0 | 40.4 | 15.0 | 3.1 |
| METi_245 | METi_245.3 | 1.2 | 1.6 | -19.4 | 11.1 | 40.2 | 14.9 | 3.1 |
| METi_245 | METi_245.4 | 1.6 | 2.1 | -19.5 | 10.9 | 42.0 | 15.5 | 3.2 |
| METi_245 | METi_245.5 | 2.1 | 2.5 | -19.6 | 10.5 | 39.1 | 14.5 | 3.1 |
| METi_245 | METi_245.6 | 2.5 | 3.0 | -19.6 | 10.7 | 41.8 | 15.5 | 3.1 |
| METi_245 | METi_245.7 | 3.0 | 3.6 | -19.1 | 10.7 | 41.2 | 15.1 | 3.2 |
| METi_245 | METi_245.8 | 3.6 | 4.2 | -19.7 | 10.4 | 42.0 | 15.6 | 3.1 |
| METi_245 | METi_245.9 | 4.2 | 4.9 | -19.6 | 10.8 | 42.5 | 15.7 | 3.2 |
| METi_245 | METi_245.10 | 4.9 | 5.5 | -19.1 | 10.7 | 41.2 | 15.3 | 3.1 |
| METi_245 | METi_245.11 | 5.5 | 6.2 | -19.1 | 10.7 | 39.1 | 14.5 | 3.2 |
| METi_245 | METi_245.12 | 6.2 | 6.9 | -18.8 | 10.7 | 38.0 | 14.0 | 3.2 |
| METi_245 | METi_245.13 | 6.9 | 7.5 | n.a. | n.a. | n.a. | n.a. | n.a. |
| METi_245 | METi_245.14 | 7.5 | 8.1 | n.a. | n.a. | n.a. | n.a. | n.a. |
| METi_245 | METi_245.15 | 8.1 | 8.7 | n.a. | n.a. | n.a. | n.a. | n.a. |
| METi_245 | METi_245.16 | 8.7 | 9.3 | n.a. | n.a. | n.a. | n.a. | n.a. |
| METi_239 | METi_239.1 | 0.3 | 0.8 | -18.9 | 12.6 | 42.3 | 15.5 | 3.2 |
| METi_239 | METi_239.2 | 0.8 | 1.4 | -18.9 | 12.2 | 41.4 | 15.3 | 3.2 |
| METi_239 | METi_239.3 | 1.4 | 1.9 | -18.9 | 10.4 | 41.9 | 15.5 | 3.2 |
| METi_239 | METi_239.4 | 1.9 | 2.5 | -19.0 | 10.0 | 41.3 | 15.2 | 3.2 |
| METi_239 | METi_239.5 | 2.5 | 3.1 | -19.1 | 9.9 | 40.1 | 14.8 | 3.2 |
| METi_239 | METi_239.6 | 3.1 | 3.8 | -19.3 | 9.9 | 41.7 | 15.3 | 3.2 |
| METi_239 | METi_239.7 | 3.8 | 4.6 | -19.4 | 10.1 | 43.8 | 16.1 | 3.2 |
| METi_239 | METi_239.8 | 4.6 | 5.4 | -19.3 | 9.9 | 41.5 | 15.1 | 3.2 |
| METi_239 | METi_239.9 | 5.4 | 6.2 | -19.3 | 10.1 | 43.2 | 15.7 | 3.2 |
| METi_239 | METi_239.10 | 6.2 | 7.1 | -19.1 | 10.1 | 42.9 | 15.7 | 3.2 |
| METi_239 | METi_239.11 | 7.1 | 7.9 | n.a. | n.a. | n.a. | n.a. | n.a. |
| METi_239 | METi_239.12 | 7.9 | 8.6 | n.a. | n.a. | n.a. | n.a. | n.a. |
| METi_239 | METi_239.13 | 8.6 | 9.4 | n.a. | n.a. | n.a. | n.a. | n.a. |
| METi_237 | METi_237.1 | 0.2 | 0.7 | -16.7 | 15.5 | 46.8 | 17.8 | 3.1 |
| METi_237 | METi_237.2 | 0.7 | 1.1 | -16.9 | 13.0 | 43.3 | 16.5 | 3.1 |
| METi_237 | METi_237.3 | 1.1 | 1.6 | -16.8 | 15.0 | 43.6 | 16.5 | 3.1 |
| METi_237 | METi_237.4 | 1.6 | 2.1 | -17.1 | 13.1 | 42.5 | 16.1 | 3.1 |
| METi_237 | METi_237.5 | 2.1 | 2.6 | -17.0 | 12.2 | 40.6 | 15.3 | 3.1 |
| METi_237 | METi_237.6 | 2.6 | 3.1 | -17.2 | 11.9 | 42.2 | 15.7 | 3.1 |
| METi_237 | METi_237.7 | 3.1 | 3.8 | -17.3 | 11.8 | 42.9 | 16.0 | 3.1 |
| METi_237 | METi_237.8 | 3.8 | 4.4 | -17.4 | 11.4 | 41.4 | 15.4 | 3.1 |
| METi_237 | METi_237.9 | 4.4 | 5.1 | -17.4 | 11.1 | 43.5 | 16.1 | 3.1 |
| METi_237 | METi_237.10 | 5.1 | 5.8 | -17.4 | 11.1 | 41.6 | 15.4 | 3.2 |
| METi_237 | METi_237.11 | 5.8 | 6.6 | n.a. | n.a. | n.a. | n.a. | n.a. |
| METi_237 | METi_237.12 | 6.6 | 7.3 | n.a. | n.a. | n.a. | n.a. | n.a. |
| METi_237 | METi_237.13 | 7.3 | 7.9 | n.a. | n.a. | n.a. | n.a. | n.a. |
| METi_237 | METi_237.14 | 7.9 | 8.6 | n.a. | n.a. | n.a. | n.a. | n.a. |
| METi_237 | METi_237.15 | 8.6 | 9.2 | n.a. | n.a. | n.a. | n.a. | n.a. |
| METi_223 | METi_223.1 | 0.3 | 0.6 | -17.9 | 12.9 | 39.7 | 14.9 | 3.1 |
| METi_223 | METi_223.2 | 0.6 | 1.0 | -18.2 | 11.7 | 43.0 | 16.0 | 3.1 |
| METi_223 | METi_223.3 | 1.0 | 1.3 | -18.6 | 10.4 | 45.1 | 16.8 | 3.1 |
| METi_223 | METi_223.4 | 1.3 | 1.7 | -18.6 | 10.3 | 42.5 | 15.8 | 3.1 |
| METi_223 | METi_223.5 | 1.7 | 2.0 | -18.5 | 10.5 | 41.9 | 15.5 | 3.2 |
| METi_223 | METi_223.6 | 2.0 | 2.4 | -18.5 | 10.3 | 40.0 | 14.8 | 3.2 |
| METi_223 | METi_223.7 | 2.4 | 2.8 | -18.6 | 10.4 | 43.0 | 15.9 | 3.2 |
| METi_223 | METi_223.8 | 2.8 | 3.2 | -18.7 | 10.4 | 41.4 | 15.3 | 3.2 |
| METi_223 | METi_223.9 | 3.2 | 3.7 | -18.7 | 10.2 | 43.9 | 16.3 | 3.2 |
| METi_223 | METi_223.10 | 3.7 | 4.2 | -18.7 | 10.2 | 39.7 | 14.7 | 3.2 |
| METi_223 | METi_223.11 | 4.2 | 4.7 | -18.8 | 10.4 | 43.6 | 16.1 | 3.2 |
| METi_223 | METi_223.12 | 4.7 | 5.2 | -18.7 | 10.6 | 41.2 | 15.2 | 3.2 |
| METi_223 | METi_223.13 | 5.2 | 5.7 | -18.7 | 10.8 | 43.5 | 16.0 | 3.2 |
| METi_223 | METi_223.14 | 5.7 | 6.2 | n.a. | n.a. | n.a. | n.a. | n.a. |
| METi_223 | METi_223.15 | 6.2 | 6.8 | n.a. | n.a. | n.a. | n.a. | n.a. |
| METi_223 | METi_223.16 | 6.8 | 7.3 | n.a. | n.a. | n.a. | n.a. | n.a. |
| METi_223 | METi_223.17 | 7.3 | 7.8 | n.a. | n.a. | n.a. | n.a. | n.a. |
| METi_223 | METi_223.18 | 7.8 | 8.3 | n.a. | n.a. | n.a. | n.a. | n.a. |
| METi_223 | METi_223.19 | 8.3 | 8.7 | n.a. | n.a. | n.a. | n.a. | n.a. |
| METi_223 | METi_223.20 | 8.7 | 9.2 | n.a. | n.a. | n.a. | n.a. | n.a. |
| METi_221 | METi_221.1 | 0.3 | 0.7 | -18.7 | 14.1 | 44.1 | 16.0 | 3.2 |
| METi_221 | METi_221.2 | 0.7 | 1.2 | -19.1 | 12.2 | 43.1 | 15.8 | 3.2 |
| METi_221 | METi_221.3 | 1.2 | 1.6 | -19.0 | 10.8 | 40.2 | 14.7 | 3.2 |
| METi_221 | METi_221.4 | 1.6 | 2.1 | -18.7 | 10.6 | 41.8 | 15.6 | 3.1 |
| METi_221 | METi_221.5 | 2.1 | 2.5 | -18.8 | 10.3 | 41.2 | 15.3 | 3.1 |
| METi_221 | METi_221.6 | 2.5 | 3.0 | -18.8 | 10.2 | 44.1 | 16.2 | 3.2 |
| METi_221 | METi_221.7 | 3.0 | 3.6 | -18.6 | 10.1 | 41.0 | 15.2 | 3.1 |
| METi_221 | METi_221.8 | 3.6 | 4.2 | -18.7 | 10.0 | 40.7 | 15.0 | 3.2 |
| METi_221 | METi_221.9 | 4.2 | 4.9 | -18.8 | 10.1 | 44.5 | 16.3 | 3.2 |
| METi_221 | METi_221.10 | 4.9 | 5.5 | -19.1 | 10.2 | 42.8 | 15.0 | 3.3 |
| METi_221 | METi_221.11 | 5.5 | 6.2 | -19.4 | 10.4 | 42.3 | 14.9 | 3.3 |
| METi_221 | METi_221.12 | 6.2 | 6.9 | -19.4 | 10.7 | 44.8 | 16.1 | 3.2 |
| METi_221 | METi_221.13 | 6.9 | 7.5 | n.a. | n.a. | n.a. | n.a. | n.a. |
| METi_221 | METi_221.14 | 7.5 | 8.1 | n.a. | n.a. | n.a. | n.a. | n.a. |
| METi_221 | METi_221.15 | 8.1 | 8.7 | n.a. | n.a. | n.a. | n.a. | n.a. |
| METi_221 | METi_221.16 | 8.7 | 9.3 | n.a. | n.a. | n.a. | n.a. | n.a. |
| METi_213 | METi_213.1 | 0.3 | 0.8 | -18.6 | 12.7 | 40.3 | 14.9 | 3.2 |
| METi_213 | METi_213.2 | 0.8 | 1.2 | -19.7 | 9.7 | 41.7 | 15.4 | 3.2 |
| METi_213 | METi_213.3 | 1.2 | 1.7 | -19.2 | 9.6 | 40.7 | 15.1 | 3.1 |
| METi_213 | METi_213.4 | 1.7 | 2.2 | -19.6 | 9.5 | 42.6 | 15.8 | 3.1 |
| METi_213 | METi_213.5 | 2.2 | 2.7 | -19.2 | 9.9 | 43.5 | 16.1 | 3.1 |
| METi_213 | METi_213.6 | 2.7 | 3.2 | -19.3 | 9.9 | 43.4 | 16.1 | 3.1 |
| METi_213 | METi_213.7 | 3.2 | 3.8 | -19.4 | 10.0 | 42.0 | 15.3 | 3.2 |
| METi_213 | METi_213.8 | 3.8 | 4.5 | -19.3 | 10.0 | 40.3 | 14.9 | 3.1 |
| METi_213 | METi_213.9 | 4.5 | 5.2 | -19.3 | 10.1 | 44.1 | 16.1 | 3.2 |
| METi_213 | METi_213.10 | 5.2 | 5.9 | -19.4 | 10.1 | 42.6 | 15.5 | 3.2 |
| METi_213 | METi_213.11 | 5.9 | 6.6 | -19.2 | 10.3 | 43.4 | 15.9 | 3.2 |
| METi_213 | METi_213.12 | 6.6 | 7.3 | n.a. | n.a. | n.a. | n.a. | n.a. |
| METi_213 | METi_213.13 | 7.3 | 7.9 | n.a. | n.a. | n.a. | n.a. | n.a. |
| METi_213 | METi_213.14 | 7.9 | 8.6 | n.a. | n.a. | n.a. | n.a. | n.a. |
| METi_213 | METi_213.15 | 8.6 | 9.2 | n.a. | n.a. | n.a. | n.a. | n.a. |
| METi_207 | METi_207.1 | 0.3 | 0.7 | -18.9 | 11.2 | 40.6 | 15.1 | 3.2 |
| METi_207 | METi_207.2 | 0.7 | 1.2 | -19.2 | 9.5 | 43.0 | 15.9 | 3.2 |
| METi_207 | METi_207.3 | 1.2 | 1.6 | -19.3 | 9.0 | 41.8 | 15.5 | 3.2 |
| METi_207 | METi_207.4 | 1.6 | 2.1 | -19.5 | 8.9 | 39.5 | 14.5 | 3.2 |
| METi_207 | METi_207.5 | 2.1 | 2.5 | -19.2 | 8.6 | 40.0 | 14.5 | 3.2 |
| METi_207 | METi_207.6 | 2.5 | 3.0 | -18.9 | 8.7 | 41.4 | 15.1 | 3.2 |
| METi_207 | METi_207.7 | 3.0 | 3.6 | -18.7 | 8.9 | 42.5 | 15.4 | 3.2 |
| METi_207 | METi_207.8 | 3.6 | 4.2 | -18.9 | 8.8 | 43.2 | 15.9 | 3.2 |
| METi_207 | METi_207.9 | 4.2 | 4.9 | -19.0 | 8.8 | 43.2 | 15.8 | 3.2 |
| METi_207 | METi_207.10 | 4.9 | 5.5 | -19.4 | 8.8 | 43.6 | 15.7 | 3.2 |
| METi_207 | METi_207.11 | 5.5 | 6.2 | -19.2 | 8.9 | 42.4 | 15.7 | 3.2 |
| METi_207 | METi_207.12 | 6.2 | 6.9 | -19.2 | 8.9 | 42.0 | 15.4 | 3.2 |
| METi_207 | METi_207.13 | 6.9 | 7.5 | n.a. | n.a. | n.a. | n.a. | n.a. |
| METi_207 | METi_207.14 | 7.5 | 8.1 | n.a. | n.a. | n.a. | n.a. | n.a. |
| METi_207 | METi_207.15 | 8.1 | 8.7 | n.a. | n.a. | n.a. | n.a. | n.a. |
| METi_207 | METi_207.16 | 8.7 | 9.3 | n.a. | n.a. | n.a. | n.a. | n.a. |
| METi_203 | METi_203.1 | 0.3 | 0.8 | -16.2 | 12.5 | 43.3 | 15.9 | 3.2 |
| METi_203 | METi_203.2 | 0.8 | 1.2 | -16.3 | 11.2 | 41.8 | 15.5 | 3.2 |
| METi_203 | METi_203.3 | 1.2 | 1.7 | -14.7 | 11.5 | 42.2 | 15.7 | 3.1 |
| METi_203 | METi_203.4 | 1.7 | 2.2 | -13.7 | 11.7 | 42.5 | 15.7 | 3.2 |
| METi_203 | METi_203.5 | 2.2 | 2.7 | -13.3 | 11.8 | 43.1 | 15.9 | 3.2 |
| METi_203 | METi_203.6 | 2.7 | 3.2 | -13.6 | 11.6 | 42.4 | 15.6 | 3.2 |
| METi_203 | METi_203.7 | 3.2 | 3.8 | -14.1 | 11.5 | 41.4 | 15.3 | 3.2 |
| METi_203 | METi_203.8 | 3.8 | 4.5 | -14.9 | 11.4 | 43.0 | 15.8 | 3.2 |
| METi_203 | METi_203.9 | 4.5 | 5.2 | -15.3 | 11.4 | 42.1 | 15.4 | 3.2 |
| METi_203 | METi_203.10 | 5.2 | 5.9 | -15.8 | 11.5 | 42.0 | 15.4 | 3.2 |
| METi_203 | METi_203.11 | 5.9 | 6.6 | -16.6 | 11.3 | 41.8 | 15.3 | 3.2 |
| METi_203 | METi_203.12 | 6.6 | 7.3 | n.a. | n.a. | n.a. | n.a. | n.a. |
| METi_203 | METi_203.13 | 7.3 | 7.9 | n.a. | n.a. | n.a. | n.a. | n.a. |
| METi_203 | METi_203.14 | 7.9 | 8.6 | n.a. | n.a. | n.a. | n.a. | n.a. |
| METi_203 | METi_203.15 | 8.6 | 9.2 | n.a. | n.a. | n.a. | n.a. | n.a. |
| METi_197 | METi_197.1 | 0.3 | 0.8 | -18.7 | 9.1 | 44.2 | 16.4 | 3.1 |
| METi_197 | METi_197.2 | 0.8 | 1.2 | -18.6 | 8.8 | 42.9 | 16.1 | 3.1 |
| METi_197 | METi_197.3 | 1.2 | 1.7 | -18.5 | 9.2 | 43.2 | 16.2 | 3.1 |
| METi_197 | METi_197.4 | 1.7 | 2.2 | -18.6 | 8.6 | 43.2 | 16.1 | 3.1 |
| METi_197 | METi_197.5 | 2.2 | 2.7 | -18.5 | 8.4 | 42.8 | 16.0 | 3.1 |
| METi_197 | METi_197.6 | 2.7 | 3.2 | -18.6 | 8.5 | 42.4 | 15.6 | 3.2 |
| METi_197 | METi_197.7 | 3.2 | 3.8 | -18.5 | 8.9 | 42.8 | 15.7 | 3.2 |
| METi_197 | METi_197.8 | 3.8 | 4.5 | -18.6 | 8.6 | 42.4 | 15.6 | 3.2 |
| METi_197 | METi_197.9 | 4.5 | 5.2 | -18.6 | 8.7 | 42.0 | 15.5 | 3.2 |
| METi_197 | METi_197.10 | 5.2 | 5.9 | -18.7 | 9.3 | 42.7 | 15.6 | 3.2 |
| METi_197 | METi_197.11 | 5.9 | 6.6 | n.a. | n.a. | n.a. | n.a. | n.a. |
| METi_197 | METi_197.12 | 6.6 | 7.3 | n.a. | n.a. | n.a. | n.a. | n.a. |
| METi_197 | METi_197.13 | 7.3 | 7.9 | n.a. | n.a. | n.a. | n.a. | n.a. |
| METi_197 | METi_197.14 | 7.9 | 8.6 | n.a. | n.a. | n.a. | n.a. | n.a. |
| METi_197 | METi_197.15 | 8.6 | 9.2 | n.a. | n.a. | n.a. | n.a. | n.a. |
| METi_195 | METi_195.1 | 0.3 | 0.7 | -18.2 | 15.2 | 42.1 | 15.7 | 3.1 |
| METi_195 | METi_195.2 | 0.7 | 1.1 | -18.3 | 12.7 | 31.4 | 11.8 | 3.1 |
| METi_195 | METi_195.3 | 1.1 | 1.5 | -18.8 | 11.2 | 20.5 | 7.6 | 3.1 |
| METi_195 | METi_195.4 | 1.5 | 1.9 | -18.9 | 10.7 | 23.5 | 8.7 | 3.1 |
| METi_195 | METi_195.5 | 1.9 | 2.3 | -18.7 | 10.5 | 23.4 | 8.6 | 3.2 |
| METi_195 | METi_195.6 | 2.3 | 2.7 | -18.7 | 10.6 | 28.6 | 10.5 | 3.2 |
| METi_195 | METi_195.7 | 2.7 | 3.1 | -18.8 | 10.7 | 30.6 | 11.3 | 3.2 |
| METi_195 | METi_195.8 | 3.1 | 3.6 | -19.1 | 10.6 | 43.1 | 15.9 | 3.2 |
| METi_195 | METi_195.9 | 3.6 | 4.2 | -19.1 | 10.6 | 42.4 | 15.5 | 3.2 |
| METi_195 | METi_195.10 | 4.2 | 4.8 | -18.9 | 10.7 | 42.8 | 15.9 | 3.2 |
| METi_195 | METi_195.11 | 4.8 | 5.4 | -18.9 | 10.7 | 42.2 | 15.4 | 3.2 |
| METi_195 | METi_195.12 | 5.4 | 5.9 | -18.9 | 11.1 | 41.7 | 15.2 | 3.2 |
| METi_195 | METi_195.13 | 5.9 | 6.5 | -18.9 | 11.0 | 41.8 | 15.5 | 3.2 |
| METi_195 | METi_195.14 | 6.5 | 7.1 | -19.0 | 10.9 | 40.6 | 14.9 | 3.2 |
| METi_195 | METi_195.15 | 7.1 | 7.7 | n.a. | n.a. | n.a. | n.a. | n.a. |
| METi_195 | METi_195.16 | 7.7 | 8.2 | n.a. | n.a. | n.a. | n.a. | n.a. |
| METi_195 | METi_195.17 | 8.2 | 8.7 | n.a. | n.a. | n.a. | n.a. | n.a. |
| METi_195 | METi_195.18 | 8.7 | 9.3 | n.a. | n.a. | n.a. | n.a. | n.a. |
| METi_193 | METi_193.1 | 0.3 | 0.7 | -18.7 | 10.7 | 42.6 | 15.7 | 3.2 |
| METi_193 | METi_193.2 | 0.7 | 1.1 | -19.6 | 8.1 | 43.3 | 16.0 | 3.2 |
| METi_193 | METi_193.3 | 1.1 | 1.5 | -20.1 | 7.6 | 43.6 | 16.0 | 3.2 |
| METi_193 | METi_193.4 | 1.5 | 1.9 | -19.8 | 7.8 | 43.4 | 15.9 | 3.2 |
| METi_193 | METi_193.5 | 1.9 | 2.3 | -19.4 | 7.7 | 42.9 | 15.7 | 3.2 |
| METi_193 | METi_193.6 | 2.3 | 2.7 | -19.1 | 7.8 | 45.9 | 16.8 | 3.2 |
| METi_193 | METi_193.7 | 2.7 | 3.1 | -19.0 | 7.8 | 41.6 | 15.2 | 3.2 |
| METi_193 | METi_193.8 | 3.1 | 3.6 | -19.2 | 7.8 | 43.0 | 15.8 | 3.2 |
| METi_193 | METi_193.9 | 3.6 | 4.2 | -19.2 | 8.0 | 44.4 | 16.3 | 3.2 |
| METi_193 | METi_193.10 | 4.2 | 4.8 | -19.5 | 8.1 | 43.9 | 16.1 | 3.2 |
| METi_193 | METi_193.11 | 4.8 | 5.4 | -19.6 | 8.3 | 43.0 | 15.7 | 3.2 |
| METi_193 | METi_193.12 | 5.4 | 5.9 | -19.5 | 8.4 | 43.2 | 15.9 | 3.2 |
| METi_193 | METi_193.13 | 5.9 | 6.5 | n.a. | n.a. | n.a. | n.a. | n.a. |
| METi_193 | METi_193.14 | 6.5 | 7.1 | n.a. | n.a. | n.a. | n.a. | n.a. |
| METi_193 | METi_193.15 | 7.1 | 7.7 | n.a. | n.a. | n.a. | n.a. | n.a. |
| METi_193 | METi_193.16 | 7.7 | 8.2 | n.a. | n.a. | n.a. | n.a. | n.a. |
| METi_193 | METi_193.17 | 8.2 | 8.7 | n.a. | n.a. | n.a. | n.a. | n.a. |
| METi_193 | METi_193.18 | 8.7 | 9.3 | n.a. | n.a. | n.a. | n.a. | n.a. |
| METi_191 | METi_191.1 | 0.3 | 0.6 | -18.6 | 12.8 | 41.6 | 15.3 | 3.2 |
| METi_191 | METi_191.2 | 0.6 | 1.0 | -18.8 | 12.0 | 41.7 | 15.5 | 3.1 |
| METi_191 | METi_191.3 | 1.0 | 1.3 | -19.0 | 10.9 | 43.2 | 16.1 | 3.1 |
| METi_191 | METi_191.4 | 1.3 | 1.7 | -19.2 | 10.5 | 42.1 | 15.7 | 3.1 |
| METi_191 | METi_191.5 | 1.7 | 2.0 | -19.3 | 10.2 | 41.2 | 15.4 | 3.1 |
| METi_191 | METi_191.6 | 2.0 | 2.4 | -19.3 | 10.1 | 41.1 | 15.3 | 3.1 |
| METi_191 | METi_191.7 | 2.4 | 2.8 | -19.5 | 10.0 | 41.7 | 15.5 | 3.1 |
| METi_191 | METi_191.8 | 2.8 | 3.2 | -19.5 | 10.0 | 42.0 | 15.5 | 3.2 |
| METi_191 | METi_191.9 | 3.2 | 3.7 | -19.4 | 10.1 | 41.7 | 15.4 | 3.2 |
| METi_191 | METi_191.10 | 3.7 | 4.2 | -19.4 | 10.0 | 41.3 | 15.2 | 3.2 |
| METi_191 | METi_191.11 | 4.2 | 4.7 | -19.4 | 10.0 | 43.0 | 15.9 | 3.1 |
| METi_191 | METi_191.12 | 4.7 | 5.2 | -19.4 | 9.8 | 43.2 | 16.0 | 3.1 |
| METi_191 | METi_191.13 | 5.2 | 5.7 | -19.3 | 10.0 | 40.3 | 15.0 | 3.1 |
| METi_191 | METi_191.14 | 5.7 | 6.2 | n.a. | n.a. | n.a. | n.a. | n.a. |
| METi_191 | METi_191.15 | 6.2 | 6.8 | n.a. | n.a. | n.a. | n.a. | n.a. |
| METi_191 | METi_191.16 | 6.8 | 7.3 | n.a. | n.a. | n.a. | n.a. | n.a. |
| METi_191 | METi_191.17 | 7.3 | 7.8 | n.a. | n.a. | n.a. | n.a. | n.a. |
| METi_191 | METi_191.18 | 7.8 | 8.3 | n.a. | n.a. | n.a. | n.a. | n.a. |
| METi_191 | METi_191.19 | 8.3 | 8.7 | n.a. | n.a. | n.a. | n.a. | n.a. |
| METi_191 | METi_191.20 | 8.7 | 9.2 | n.a. | n.a. | n.a. | n.a. | n.a. |
| METi_189 | METi_189.1 | 0.3 | 0.7 | -18.1 | 12.2 | 42.2 | 15.8 | 3.1 |
| METi_189 | METi_189.2 | 0.7 | 1.1 | -18.3 | 11.0 | 41.1 | 15.3 | 3.1 |
| METi_189 | METi_189.3 | 1.1 | 1.5 | -18.6 | 10.5 | 41.2 | 15.3 | 3.1 |
| METi_189 | METi_189.4 | 1.5 | 2.0 | -18.9 | 10.1 | 41.6 | 15.5 | 3.1 |
| METi_189 | METi_189.5 | 2.0 | 2.4 | -18.9 | 10.6 | 41.7 | 15.5 | 3.1 |
| METi_189 | METi_189.6 | 2.4 | 2.8 | -18.9 | 10.6 | 41.8 | 15.5 | 3.1 |
| METi_189 | METi_189.7 | 2.8 | 3.4 | -18.9 | 10.1 | 41.2 | 15.3 | 3.1 |
| METi_189 | METi_189.8 | 3.4 | 4.0 | -18.8 | 10.2 | 41.8 | 15.7 | 3.1 |
| METi_189 | METi_189.9 | 4.0 | 4.6 | -18.9 | 10.1 | 43.5 | 16.4 | 3.1 |
| METi_189 | METi_189.10 | 4.6 | 5.2 | -19.0 | 10.2 | 28.8 | 10.8 | 3.1 |
| METi_189 | METi_189.11 | 5.2 | 5.8 | -19.0 | 10.4 | 42.8 | 16.0 | 3.1 |
| METi_189 | METi_189.12 | 5.8 | 6.4 | -18.9 | 10.6 | 42.4 | 15.9 | 3.1 |
| METi_189 | METi_189.13 | 6.4 | 7.1 | n.a. | n.a. | n.a. | n.a. | n.a. |
| METi_189 | METi_189.14 | 7.1 | 7.6 | n.a. | n.a. | n.a. | n.a. | n.a. |
| METi_189 | METi_189.15 | 7.6 | 8.2 | n.a. | n.a. | n.a. | n.a. | n.a. |
| METi_189 | METi_189.16 | 8.2 | 8.8 | n.a. | n.a. | n.a. | n.a. | n.a. |
| METi_189 | METi_189.17 | 8.8 | 9.3 | n.a. | n.a. | n.a. | n.a. | n.a. |
| METi_187 | METi_187.1 | 0.3 | 0.7 | -18.2 | 11.5 | 41.6 | 15.0 | 3.2 |
| METi_187 | METi_187.2 | 0.7 | 1.2 | -18.6 | 10.7 | 41.7 | 15.3 | 3.2 |
| METi_187 | METi_187.3 | 1.2 | 1.6 | -19.1 | 10.1 | 41.3 | 15.1 | 3.2 |
| METi_187 | METi_187.4 | 1.6 | 2.1 | -19.4 | 9.5 | 41.0 | 14.8 | 3.2 |
| METi_187 | METi_187.5 | 2.1 | 2.5 | -19.3 | 9.5 | 40.8 | 14.8 | 3.2 |
| METi_187 | METi_187.6 | 2.5 | 3.0 | -19.0 | 9.4 | 41.9 | 15.1 | 3.2 |
| METi_187 | METi_187.7 | 3.0 | 3.6 | -18.9 | 9.5 | 41.3 | 15.0 | 3.2 |
| METi_187 | METi_187.8 | 3.6 | 4.2 | -18.7 | 9.5 | 44.3 | 16.1 | 3.2 |
| METi_187 | METi_187.9 | 4.2 | 4.9 | -18.9 | 9.7 | 40.4 | 14.6 | 3.2 |
| METi_187 | METi_187.10 | 4.9 | 5.5 | -18.9 | 9.5 | 42.0 | 15.1 | 3.3 |
| METi_187 | METi_187.11 | 5.5 | 6.2 | -18.8 | 10.0 | 41.6 | 15.1 | 3.2 |
| METi_187 | METi_187.12 | 6.2 | 6.9 | n.a. | n.a. | n.a. | n.a. | n.a. |
| METi_187 | METi_187.13 | 6.9 | 7.5 | n.a. | n.a. | n.a. | n.a. | n.a. |
| METi_187 | METi_187.14 | 7.5 | 8.1 | n.a. | n.a. | n.a. | n.a. | n.a. |
| METi_187 | METi_187.15 | 8.1 | 8.7 | n.a. | n.a. | n.a. | n.a. | n.a. |
| METi_187 | METi_187.16 | 8.7 | 9.3 | n.a. | n.a. | n.a. | n.a. | n.a. |
| METi_169 | METi_169.1 | 0.3 | 0.8 | -18.6 | 13.5 | 42.4 | 15.7 | 3.2 |
| METi_169 | METi_169.2 | 0.8 | 1.2 | -18.7 | 13.6 | 42.5 | 15.8 | 3.1 |
| METi_169 | METi_169.3 | 1.2 | 1.7 | -18.9 | 11.9 | 41.5 | 15.5 | 3.1 |
| METi_169 | METi_169.4 | 1.7 | 2.2 | -19.1 | 10.8 | 42.2 | 15.7 | 3.1 |
| METi_169 | METi_169.5 | 2.2 | 2.7 | -19.2 | 10.4 | 41.5 | 15.5 | 3.1 |
| METi_169 | METi_169.6 | 2.7 | 3.2 | -19.1 | 9.8 | 40.4 | 15.0 | 3.1 |
| METi_169 | METi_169.7 | 3.2 | 3.8 | -19.0 | 9.8 | 40.8 | 15.1 | 3.2 |
| METi_169 | METi_169.8 | 3.8 | 4.5 | -18.9 | 9.2 | 42.0 | 15.6 | 3.1 |
| METi_169 | METi_169.9 | 4.5 | 5.2 | -18.7 | 8.8 | 41.7 | 15.5 | 3.2 |
| METi_169 | METi_169.10 | 5.2 | 5.9 | -18.8 | 8.7 | 41.4 | 15.4 | 3.1 |
| METi_169 | METi_169.11 | 5.9 | 6.6 | -18.8 | 8.5 | 43.4 | 16.0 | 3.2 |
| METi_169 | METi_169.12 | 6.6 | 7.3 | -18.9 | 8.6 | 42.2 | 15.7 | 3.1 |
| METi_169 | METi_169.13 | 7.3 | 7.9 | n.a. | n.a. | n.a. | n.a. | n.a. |
| METi_169 | METi_169.14 | 7.9 | 8.6 | n.a. | n.a. | n.a. | n.a. | n.a. |
| METi_169 | METi_169.15 | 8.6 | 9.2 | n.a. | n.a. | n.a. | n.a. | n.a. |
| METi_163 | METi_163.1 | 0.3 | 0.7 | -18.4 | 11.4 | 43.0 | 15.8 | 3.2 |
| METi_163 | METi_163.2 | 0.7 | 1.0 | -18.3 | 11.3 | 41.0 | 15.2 | 3.2 |
| METi_163 | METi_163.3 | 1.0 | 1.4 | -18.5 | 10.3 | 41.0 | 15.2 | 3.2 |
| METi_163 | METi_163.4 | 1.4 | 1.8 | -18.7 | 10.1 | 41.7 | 15.4 | 3.2 |
| METi_163 | METi_163.5 | 1.8 | 2.2 | -18.4 | 10.1 | 41.4 | 15.4 | 3.2 |
| METi_163 | METi_163.6 | 2.2 | 2.6 | -18.1 | 10.1 | 42.4 | 15.7 | 3.2 |
| METi_163 | METi_163.7 | 2.6 | 2.9 | -17.9 | 10.0 | 42.7 | 15.8 | 3.2 |
| METi_163 | METi_163.8 | 2.9 | 3.5 | -18.0 | 9.8 | 41.3 | 15.3 | 3.2 |
| METi_163 | METi_163.9 | 3.5 | 4.0 | -18.1 | 9.9 | 41.6 | 15.4 | 3.2 |
| METi_163 | METi_163.10 | 4.0 | 4.5 | -18.2 | 9.9 | 42.4 | 15.7 | 3.2 |
| METi_163 | METi_163.11 | 4.5 | 5.1 | -18.2 | 10.1 | 38.7 | 14.3 | 3.2 |
| METi_163 | METi_163.12 | 5.1 | 5.6 | -18.2 | 10.2 | 40.9 | 15.1 | 3.2 |
| METi_163 | METi_163.13 | 5.6 | 6.2 | -18.4 | 10.2 | 40.2 | 14.8 | 3.2 |
| METi_163 | METi_163.14 | 6.2 | 6.7 | n.a. | n.a. | n.a. | n.a. | n.a. |
| METi_163 | METi_163.15 | 6.7 | 7.3 | n.a. | n.a. | n.a. | n.a. | n.a. |
| METi_163 | METi_163.16 | 7.3 | 7.8 | n.a. | n.a. | n.a. | n.a. | n.a. |
| METi_163 | METi_163.17 | 7.8 | 8.3 | n.a. | n.a. | n.a. | n.a. | n.a. |
| METi_163 | METi_163.18 | 8.3 | 8.8 | n.a. | n.a. | n.a. | n.a. | n.a. |
| METi_163 | METi_163.19 | 8.8 | 9.3 | n.a. | n.a. | n.a. | n.a. | n.a. |
| METi_157 | METi_157.1 | 0.3 | 0.6 | -19.9 | 11.6 | 41.2 | 15.2 | 3.2 |
| METi_157 | METi_157.2 | 0.6 | 1.0 | -19.8 | 11.1 | 41.6 | 15.5 | 3.1 |
| METi_157 | METi_157.3 | 1.0 | 1.3 | -19.4 | 10.5 | 40.7 | 15.1 | 3.1 |
| METi_157 | METi_157.4 | 1.3 | 1.7 | -19.3 | 10.6 | 42.2 | 15.6 | 3.2 |
| METi_157 | METi_157.5 | 1.7 | 2.0 | -19.5 | 10.6 | 40.7 | 15.1 | 3.2 |
| METi_157 | METi_157.6 | 2.0 | 2.4 | -19.8 | 10.5 | 40.6 | 15.1 | 3.2 |
| METi_157 | METi_157.7 | 2.4 | 2.8 | -19.5 | 10.3 | 42.8 | 15.8 | 3.2 |
| METi_157 | METi_157.8 | 2.8 | 3.2 | -18.9 | 9.8 | 43.5 | 16.0 | 3.2 |
| METi_157 | METi_157.9 | 3.2 | 3.7 | -18.6 | 9.5 | 41.8 | 15.5 | 3.2 |
| METi_157 | METi_157.10 | 3.7 | 4.2 | -18.6 | 9.6 | 44.1 | 16.2 | 3.2 |
| METi_157 | METi_157.11 | 4.2 | 4.7 | -18.7 | 9.8 | 42.4 | 15.6 | 3.2 |
| METi_157 | METi_157.12 | 4.7 | 5.2 | -18.8 | 9.9 | 42.4 | 15.5 | 3.2 |
| METi_157 | METi_157.13 | 5.2 | 5.7 | -18.8 | 9.8 | 41.5 | 15.3 | 3.2 |
| METi_157 | METi_157.14 | 5.7 | 6.2 | -18.9 | 9.9 | 40.8 | 15.0 | 3.2 |
| METi_157 | METi_157.15 | 6.2 | 6.8 | -19.1 | 9.9 | 38.9 | 14.2 | 3.2 |
| METi_157 | METi_157.16 | 6.8 | 7.3 | n.a. | n.a. | n.a. | n.a. | n.a. |
| METi_157 | METi_157.17 | 7.3 | 7.8 | n.a. | n.a. | n.a. | n.a. | n.a. |
| METi_157 | METi_157.18 | 7.8 | 8.3 | n.a. | n.a. | n.a. | n.a. | n.a. |
| METi_157 | METi_157.19 | 8.3 | 8.7 | n.a. | n.a. | n.a. | n.a. | n.a. |
| METi_157 | METi_157.20 | 8.7 | 9.2 | n.a. | n.a. | n.a. | n.a. | n.a. |
| METi_144 | METi_144.1 | 0.3 | 0.7 | -15.6 | 13.0 | 41.0 | 15.4 | 3.1 |
| METi_144 | METi_144.2 | 0.7 | 1.2 | -15.8 | 12.0 | 42.4 | 15.9 | 3.1 |
| METi_144 | METi_144.3 | 1.2 | 1.6 | -16.3 | 11.5 | 38.9 | 14.6 | 3.1 |
| METi_144 | METi_144.4 | 1.6 | 2.1 | -16.5 | 11.3 | 41.6 | 15.5 | 3.1 |
| METi_144 | METi_144.5 | 2.1 | 2.5 | -16.5 | 11.3 | 40.3 | 15.1 | 3.1 |
| METi_144 | METi_144.6 | 2.5 | 3.0 | -16.3 | 11.4 | 40.8 | 15.2 | 3.1 |
| METi_144 | METi_144.7 | 3.0 | 3.6 | -16.3 | 11.6 | 41.8 | 15.5 | 3.1 |
| METi_144 | METi_144.8 | 3.6 | 4.2 | -16.3 | 11.6 | 41.5 | 15.4 | 3.2 |
| METi_144 | METi_144.9 | 4.2 | 4.9 | -16.3 | 11.9 | 37.5 | 13.9 | 3.2 |
| METi_144 | METi_144.10 | 4.9 | 5.5 | -16.2 | 12.1 | 41.4 | 15.3 | 3.2 |
| METi_144 | METi_144.11 | 5.5 | 6.2 | -16.1 | 12.2 | 39.4 | 14.7 | 3.1 |
| METi_144 | METi_144.12 | 6.2 | 6.9 | n.a. | n.a. | n.a. | n.a. | n.a. |
| METi_144 | METi_144.13 | 6.9 | 7.5 | n.a. | n.a. | n.a. | n.a. | n.a. |
| METi_144 | METi_144.14 | 7.5 | 8.1 | n.a. | n.a. | n.a. | n.a. | n.a. |
| METi_144 | METi_144.15 | 8.1 | 8.7 | n.a. | n.a. | n.a. | n.a. | n.a. |
| METi_144 | METi_144.16 | 8.7 | 9.3 | n.a. | n.a. | n.a. | n.a. | n.a. |
| METi_141 | METi_141.1 | 0.3 | 0.7 | -18.6 | 13.4 | 41.2 | 15.3 | 3.1 |
| METi_141 | METi_141.2 | 0.7 | 1.1 | -19.3 | 11.3 | 43.3 | 16.1 | 3.1 |
| METi_141 | METi_141.3 | 1.1 | 1.5 | -19.2 | 10.6 | 41.2 | 15.3 | 3.1 |
| METi_141 | METi_141.4 | 1.5 | 1.9 | -19.4 | 10.7 | 42.2 | 15.6 | 3.2 |
| METi_141 | METi_141.5 | 1.9 | 2.3 | -19.6 | 11.2 | 41.2 | 15.2 | 3.2 |
| METi_141 | METi_141.6 | 2.3 | 2.7 | -19.5 | 10.8 | 38.9 | 14.5 | 3.1 |
| METi_141 | METi_141.7 | 2.7 | 3.1 | -19.0 | 10.7 | 40.0 | 14.9 | 3.1 |
| METi_141 | METi_141.8 | 3.1 | 3.6 | -18.7 | 10.9 | 41.3 | 15.3 | 3.2 |
| METi_141 | METi_141.9 | 3.6 | 4.2 | -18.6 | 11.3 | 41.8 | 15.4 | 3.2 |
| METi_141 | METi_141.10 | 4.2 | 4.8 | -19.1 | 10.5 | 40.5 | 15.0 | 3.2 |
| METi_141 | METi_141.11 | 4.8 | 5.4 | -19.0 | 10.9 | 47.8 | 17.2 | 3.2 |
| METi_141 | METi_141.12 | 5.4 | 5.9 | -18.5 | 10.7 | 46.2 | 17.2 | 3.1 |
| METi_141 | METi_141.13 | 5.9 | 6.5 | -18.8 | 10.6 | 45.2 | 16.8 | 3.2 |
| METi_141 | METi_141.14 | 6.5 | 7.1 | n.a. | n.a. | n.a. | n.a. | n.a. |
| METi_141 | METi_141.15 | 7.1 | 7.7 | n.a. | n.a. | n.a. | n.a. | n.a. |
| METi_141 | METi_141.16 | 7.7 | 8.2 | n.a. | n.a. | n.a. | n.a. | n.a. |
| METi_141 | METi_141.17 | 8.2 | 8.7 | n.a. | n.a. | n.a. | n.a. | n.a. |
| METi_141 | METi_141.18 | 8.7 | 9.3 | n.a. | n.a. | n.a. | n.a. | n.a. |
| METi_127 | METi_127.1 | 0.3 | 0.7 | -17.4 | 13.8 | 41.6 | 15.5 | 3.1 |
| METi_127 | METi_127.2 | 0.7 | 1.1 | -18.5 | 12.3 | 41.8 | 15.6 | 3.1 |
| METi_127 | METi_127.3 | 1.1 | 1.5 | -19.4 | 11.8 | 41.5 | 15.6 | 3.1 |
| METi_127 | METi_127.4 | 1.5 | 1.9 | -19.4 | 11.9 | 40.9 | 15.3 | 3.1 |
| METi_127 | METi_127.5 | 1.9 | 2.3 | -19.2 | 11.5 | 41.8 | 15.4 | 3.2 |
| METi_127 | METi_127.6 | 2.3 | 2.7 | -19.1 | 11.2 | 42.2 | 15.6 | 3.2 |
| METi_127 | METi_127.7 | 2.7 | 3.1 | -19.1 | 11.3 | 41.5 | 15.5 | 3.1 |
| METi_127 | METi_127.8 | 3.1 | 3.6 | -19.1 | 11.1 | 40.6 | 15.2 | 3.1 |
| METi_127 | METi_127.9 | 3.6 | 4.2 | -19.2 | 11.3 | 40.6 | 15.1 | 3.2 |
| METi_127 | METi_127.10 | 4.2 | 4.8 | -19.3 | 11.2 | 42.6 | 15.8 | 3.2 |
| METi_127 | METi_127.11 | 4.8 | 5.4 | -19.3 | 11.1 | 42.1 | 15.7 | 3.1 |
| METi_127 | METi_127.12 | 5.4 | 5.9 | -19.2 | 11.0 | 42.2 | 15.7 | 3.1 |
| METi_127 | METi_127.13 | 5.9 | 6.5 | -19.3 | 10.9 | 41.5 | 15.4 | 3.1 |
| METi_127 | METi_127.14 | 6.5 | 7.1 | -19.2 | 11.0 | 40.3 | 15.0 | 3.1 |
| METi_127 | METi_127.15 | 7.1 | 7.7 | n.a. | n.a. | n.a. | n.a. | n.a. |
| METi_127 | METi_127.16 | 7.7 | 8.2 | n.a. | n.a. | n.a. | n.a. | n.a. |
| METi_127 | METi_127.17 | 8.2 | 8.7 | n.a. | n.a. | n.a. | n.a. | n.a. |
| METi_127 | METi_127.18 | 8.7 | 9.3 | n.a. | n.a. | n.a. | n.a. | n.a. |
| METi_125 | METi_125.1 | 0.3 | 0.8 | -19.3 | 9.7 | 41.2 | 15.2 | 3.2 |
| METi_125 | METi_125.2 | 0.8 | 1.2 | -19.2 | 9.7 | 41.6 | 15.5 | 3.1 |
| METi_125 | METi_125.3 | 1.2 | 1.7 | -19.1 | 9.5 | 42.6 | 15.7 | 3.2 |
| METi_125 | METi_125.4 | 1.7 | 2.2 | -19.2 | 9.5 | 44.2 | 16.4 | 3.1 |
| METi_125 | METi_125.5 | 2.2 | 2.7 | -19.2 | 9.5 | 43.5 | 16.2 | 3.1 |
| METi_125 | METi_125.6 | 2.7 | 3.2 | -18.2 | 10.0 | 42.2 | 15.7 | 3.1 |
| METi_125 | METi_125.7 | 3.2 | 3.8 | -18.8 | 9.6 | 41.9 | 15.5 | 3.2 |
| METi_125 | METi_125.8 | 3.8 | 4.5 | -18.7 | 9.6 | 42.0 | 15.6 | 3.1 |
| METi_125 | METi_125.9 | 4.5 | 5.2 | -18.8 | 9.9 | 41.9 | 15.5 | 3.1 |
| METi_125 | METi_125.10 | 5.2 | 5.9 | -18.9 | 10.1 | 43.7 | 16.1 | 3.2 |
| METi_125 | METi_125.11 | 5.9 | 6.6 | -18.9 | 10.3 | 43.7 | 16.1 | 3.2 |
| METi_125 | METi_125.12 | 6.6 | 7.3 | n.a. | n.a. | n.a. | n.a. | n.a. |
| METi_125 | METi_125.13 | 7.3 | 7.9 | n.a. | n.a. | n.a. | n.a. | n.a. |
| METi_125 | METi_125.14 | 7.9 | 8.6 | n.a. | n.a. | n.a. | n.a. | n.a. |
| METi_125 | METi_125.15 | 8.6 | 9.2 | n.a. | n.a. | n.a. | n.a. | n.a. |
| METi_119 | METi_119.1 | 0.3 | 0.8 | n.a. | n.a. | n.a. | n.a. | n.a. |
| METi_119 | METi_119.2 | 0.8 | 1.3 | -17.2 | 9.4 | 40.1 | 15.0 | 3.1 |
| METi_119 | METi_119.3 | 1.3 | 1.8 | -17.2 | 8.9 | 45.0 | 16.8 | 3.1 |
| METi_119 | METi_119.4 | 1.8 | 2.3 | -16.6 | 8.9 | 43.5 | 16.2 | 3.1 |
| METi_119 | METi_119.5 | 2.3 | 2.9 | -16.4 | 8.6 | 41.7 | 15.6 | 3.1 |
| METi_119 | METi_119.6 | 2.9 | 3.6 | -16.3 | 8.4 | 42.3 | 15.8 | 3.1 |
| METi_119 | METi_119.7 | 3.6 | 4.3 | -16.0 | 8.3 | 42.4 | 15.8 | 3.1 |
| METi_119 | METi_119.8 | 4.3 | 5.0 | -16.1 | 8.0 | 44.0 | 16.4 | 3.1 |
| METi_119 | METi_119.9 | 5.0 | 5.8 | -16.1 | 7.8 | 44.0 | 16.3 | 3.1 |
| METi_119 | METi_119.10 | 5.8 | 6.5 | -16.2 | 8.0 | 43.6 | 16.2 | 3.1 |
| METi_119 | METi_119.11 | 6.5 | 7.3 | -16.4 | 8.1 | 42.9 | 15.9 | 3.1 |
| METi_119 | METi_119.12 | 7.3 | 8.0 | n.a. | n.a. | n.a. | n.a. | n.a. |
| METi_119 | METi_119.13 | 8.0 | 8.7 | n.a. | n.a. | n.a. | n.a. | n.a. |
| METi_119 | METi_119.14 | 8.7 | 9.4 | n.a. | n.a. | n.a. | n.a. | n.a. |
| METi_115 | METi_115.1 | 0.3 | 0.7 | -18.1 | 14.2 | 40.5 | 15.1 | 3.2 |
| METi_115 | METi_115.2 | 0.7 | 1.2 | -18.1 | 11.9 | 43.5 | 16.2 | 3.1 |
| METi_115 | METi_115.3 | 1.2 | 1.6 | -18.2 | 11.1 | 43.3 | 16.1 | 3.2 |
| METi_115 | METi_115.4 | 1.6 | 2.1 | -18.4 | 10.6 | 42.9 | 16.0 | 3.1 |
| METi_115 | METi_115.5 | 2.1 | 2.5 | -18.5 | 10.8 | 43.3 | 16.0 | 3.1 |
| METi_115 | METi_115.6 | 2.5 | 3.0 | -18.5 | 10.8 | 43.1 | 16.0 | 3.1 |
| METi_115 | METi_115.7 | 3.0 | 3.6 | -18.5 | 10.8 | 44.2 | 16.4 | 3.1 |
| METi_115 | METi_115.8 | 3.6 | 4.2 | -18.5 | 10.9 | 41.1 | 15.4 | 3.1 |
| METi_115 | METi_115.9 | 4.2 | 4.9 | -18.5 | 10.8 | 42.2 | 15.8 | 3.1 |
| METi_115 | METi_115.10 | 4.9 | 5.5 | -18.4 | 10.8 | 41.9 | 15.6 | 3.1 |
| METi_115 | METi_115.11 | 5.5 | 6.2 | -18.4 | 10.8 | 42.1 | 15.7 | 3.1 |
| METi_115 | METi_115.12 | 6.2 | 6.9 | -18.5 | 11.0 | 38.0 | 13.9 | 3.2 |
| METi_115 | METi_115.13 | 6.9 | 7.5 | -18.5 | 11.0 | 39.8 | 14.8 | 3.1 |
| METi_115 | METi_115.14 | 7.5 | 8.1 | n.a. | n.a. | n.a. | n.a. | n.a. |
| METi_115 | METi_115.15 | 8.1 | 8.7 | n.a. | n.a. | n.a. | n.a. | n.a. |
| METi_115 | METi_115.16 | 8.7 | 9.3 | n.a. | n.a. | n.a. | n.a. | n.a. |
| METi_99 | METi_99.1 | 0.3 | 0.8 | -17.7 | 12.5 | 41.4 | 15.4 | 3.1 |
| METi_99 | METi_99.2 | 0.8 | 1.2 | -18.0 | 12.1 | 41.6 | 15.6 | 3.1 |
| METi_99 | METi_99.3 | 1.2 | 1.7 | -18.6 | 9.9 | 42.6 | 15.9 | 3.1 |
| METi_99 | METi_99.4 | 1.7 | 2.2 | -18.2 | 8.9 | 41.8 | 15.6 | 3.1 |
| METi_99 | METi_99.5 | 2.2 | 2.7 | -18.0 | 8.8 | 40.1 | 14.9 | 3.2 |
| METi_99 | METi_99.6 | 2.7 | 3.2 | -18.5 | 9.2 | 42.0 | 15.6 | 3.1 |
| METi_99 | METi_99.7 | 3.2 | 3.8 | -18.7 | 9.1 | 39.7 | 14.8 | 3.1 |
| METi_99 | METi_99.8 | 3.8 | 4.5 | -18.8 | 9.4 | 42.2 | 15.7 | 3.1 |
| METi_99 | METi_99.9 | 4.5 | 5.2 | -18.8 | 9.6 | 41.4 | 15.4 | 3.1 |
| METi_99 | METi_99.10 | 5.2 | 5.9 | -18.9 | 9.7 | 41.4 | 15.1 | 3.2 |
| METi_99 | METi_99.11 | 5.9 | 6.6 | -19.0 | 9.6 | 42.1 | 15.7 | 3.1 |
| METi_99 | METi_99.12 | 6.6 | 7.3 | n.a. | n.a. | n.a. | n.a. | n.a. |
| METi_99 | METi_99.13 | 7.3 | 7.9 | n.a. | n.a. | n.a. | n.a. | n.a. |
| METi_99 | METi_99.14 | 7.9 | 8.6 | n.a. | n.a. | n.a. | n.a. | n.a. |
| METi_99 | METi_99.15 | 8.6 | 9.2 | n.a. | n.a. | n.a. | n.a. | n.a. |
| METi_97 | METi_97.1 | 0.3 | 0.7 | -18.2 | 13.5 | 17.8 | 6.6 | 3.1 |
| METi_97 | METi_97.2 | 0.7 | 1.1 | -18.5 | 12.0 | 22.8 | 8.6 | 3.1 |
| METi_97 | METi_97.3 | 1.1 | 1.5 | -18.9 | 10.6 | 29.9 | 11.2 | 3.1 |
| METi_97 | METi_97.4 | 1.5 | 2.0 | -18.9 | 10.4 | 30.3 | 11.4 | 3.1 |
| METi_97 | METi_97.5 | 2.0 | 2.4 | -18.9 | 10.3 | 29.1 | 11.0 | 3.1 |
| METi_97 | METi_97.6 | 2.4 | 2.8 | -19.0 | 10.0 | 26.3 | 9.9 | 3.1 |
| METi_97 | METi_97.7 | 2.8 | 3.4 | -19.6 | 10.1 | 37.7 | 12.5 | 3.5 |
| METi_97 | METi_97.8 | 3.4 | 4.0 | -19.0 | 10.1 | 35.1 | 13.2 | 3.1 |
| METi_97 | METi_97.9 | 4.0 | 4.6 | -19.0 | 10.3 | 40.1 | 15.0 | 3.1 |
| METi_97 | METi_97.10 | 4.6 | 5.2 | -19.1 | 10.3 | 37.2 | 14.0 | 3.1 |
| METi_97 | METi_97.11 | 5.2 | 5.8 | -19.1 | 10.4 | 40.6 | 15.3 | 3.1 |
| METi_97 | METi_97.12 | 5.8 | 6.5 | -18.9 | 10.8 | 42.8 | 16.0 | 3.1 |
| METi_97 | METi_97.13 | 6.5 | 7.1 | n.a. | n.a. | n.a. | n.a. | n.a. |
| METi_97 | METi_97.14 | 7.1 | 7.7 | n.a. | n.a. | n.a. | n.a. | n.a. |
| METi_97 | METi_97.15 | 7.7 | 8.2 | n.a. | n.a. | n.a. | n.a. | n.a. |
| METi_97 | METi_97.16 | 8.2 | 8.8 | n.a. | n.a. | n.a. | n.a. | n.a. |
| METi_97 | METi_97.17 | 8.8 | 9.4 | n.a. | n.a. | n.a. | n.a. | n.a. |
| METi_83 | METi_83.1 | 0.3 | 0.8 | -17.9 | 13.5 | 41.1 | 15.3 | 3.1 |
| METi_83 | METi_83.2 | 0.8 | 1.3 | -18.4 | 12.0 | 40.7 | 15.2 | 3.1 |
| METi_83 | METi_83.3 | 1.3 | 1.8 | -18.8 | 10.8 | 40.9 | 15.3 | 3.1 |
| METi_83 | METi_83.4 | 1.8 | 2.3 | -18.9 | 10.6 | 40.8 | 15.2 | 3.1 |
| METi_83 | METi_83.5 | 2.3 | 2.9 | -18.9 | 10.5 | 40.3 | 15.0 | 3.1 |
| METi_83 | METi_83.6 | 2.9 | 3.6 | -19.0 | 10.2 | 37.4 | 14.0 | 3.1 |
| METi_83 | METi_83.7 | 3.6 | 4.3 | -18.9 | 10.1 | 36.7 | 13.7 | 3.1 |
| METi_83 | METi_83.8 | 4.3 | 5.0 | -18.8 | 10.5 | 36.7 | 13.7 | 3.1 |
| METi_83 | METi_83.9 | 5.0 | 5.8 | -18.6 | 10.7 | 36.3 | 13.5 | 3.1 |
| METi_83 | METi_83.10 | 5.8 | 6.5 | -18.7 | 10.8 | 36.3 | 13.6 | 3.1 |
| METi_83 | METi_83.11 | 6.5 | 7.3 | -18.9 | 10.6 | 34.4 | 12.8 | 3.1 |
| METi_83 | METi_83.12 | 7.3 | 8.0 | n.a. | n.a. | n.a. | n.a. | n.a. |
| METi_83 | METi_83.13 | 8.0 | 8.7 | n.a. | n.a. | n.a. | n.a. | n.a. |
| METi_83 | METi_83.14 | 8.7 | 9.4 | n.a. | n.a. | n.a. | n.a. | n.a. |
| METi_71 | METi_71.1 | 0.3 | 0.8 | -15.7 | 10.2 | 41.5 | 15.4 | 3.1 |
| METi_71 | METi_71.2 | 0.8 | 1.2 | -14.7 | 9.0 | 42.4 | 15.8 | 3.1 |
| METi_71 | METi_71.3 | 1.2 | 1.7 | -15.2 | 8.4 | 41.6 | 15.5 | 3.1 |
| METi_71 | METi_71.4 | 1.7 | 2.2 | -16.1 | 7.8 | 40.8 | 15.1 | 3.2 |
| METi_71 | METi_71.5 | 2.2 | 2.7 | -15.9 | 7.7 | 42.1 | 15.6 | 3.2 |
| METi_71 | METi_71.6 | 2.7 | 3.2 | -15.3 | 8.0 | 41.4 | 15.3 | 3.2 |
| METi_71 | METi_71.7 | 3.2 | 3.8 | -15.7 | 7.9 | 41.1 | 15.3 | 3.1 |
| METi_71 | METi_71.8 | 3.8 | 4.5 | -16.2 | 8.2 | 42.4 | 15.8 | 3.1 |
| METi_71 | METi_71.9 | 4.5 | 5.2 | -16.5 | 7.9 | 43.0 | 16.0 | 3.1 |
| METi_71 | METi_71.10 | 5.2 | 5.9 | -16.2 | 8.0 | 42.2 | 15.6 | 3.2 |
| METi_71 | METi_71.11 | 5.9 | 6.6 | -15.8 | 7.9 | 41.8 | 15.5 | 3.2 |
| METi_71 | METi_71.12 | 6.6 | 7.3 | -16.0 | 7.8 | 41.8 | 15.4 | 3.2 |
| METi_71 | METi_71.13 | 7.3 | 7.9 | n.a. | n.a. | n.a. | n.a. | n.a. |
| METi_71 | METi_71.14 | 7.9 | 8.6 | n.a. | n.a. | n.a. | n.a. | n.a. |
| METi_71 | METi_71.15 | 8.6 | 9.2 | n.a. | n.a. | n.a. | n.a. | n.a. |
| METi_67 | METi_67.1 | 0.3 | 0.8 | -18.8 | 10.1 | 42.8 | 15.8 | 3.2 |
| METi_67 | METi_67.2 | 0.8 | 1.2 | -18.7 | 10.6 | 40.8 | 15.1 | 3.2 |
| METi_67 | METi_67.3 | 1.2 | 1.7 | -18.1 | 10.9 | 41.1 | 15.3 | 3.1 |
| METi_67 | METi_67.4 | 1.7 | 2.2 | -18.4 | 10.9 | 42.3 | 15.6 | 3.2 |
| METi_67 | METi_67.5 | 2.2 | 2.7 | -18.6 | 10.7 | 40.9 | 15.1 | 3.2 |
| METi_67 | METi_67.6 | 2.7 | 3.2 | -18.7 | 10.6 | 42.6 | 15.7 | 3.2 |
| METi_67 | METi_67.7 | 3.2 | 3.8 | -18.6 | 10.5 | 41.9 | 15.5 | 3.2 |
| METi_67 | METi_67.8 | 3.8 | 4.5 | -18.4 | 10.7 | 42.2 | 15.5 | 3.2 |
| METi_67 | METi_67.9 | 4.5 | 5.2 | -18.5 | 10.8 | 42.3 | 15.5 | 3.2 |
| METi_67 | METi_67.10 | 5.2 | 5.9 | -18.7 | 10.9 | 42.8 | 15.7 | 3.2 |
| METi_67 | METi_67.11 | 5.9 | 6.6 | -18.9 | 11.1 | 42.1 | 15.4 | 3.2 |
| METi_67 | METi_67.12 | 6.6 | 7.3 | -18.9 | 11.2 | 42.8 | 15.8 | 3.2 |
| METi_67 | METi_67.13 | 7.3 | 7.9 | n.a. | n.a. | n.a. | n.a. | n.a. |
| METi_67 | METi_67.14 | 7.9 | 8.6 | n.a. | n.a. | n.a. | n.a. | n.a. |
| METi_67 | METi_67.15 | 8.6 | 9.2 | n.a. | n.a. | n.a. | n.a. | n.a. |
| BN15 | BN15-M1-A | 0.3 | 0.8 | -20.6 | 12.1 | 39.6 | 14.5 | 3.2 |
| BN15 | BN15-M1-B | 0.8 | 1.2 | -21.0 | 11.5 | 41.7 | 14.9 | 3.3 |
| BN15 | BN15-M1-C | 1.2 | 1.7 | -21.1 | 10.6 | 42.7 | 15.6 | 3.2 |
| BN15 | BN15-M1-D | 1.7 | 2.2 | -21.1 | 10.2 | 39.1 | 14.5 | 3.2 |
| BN15 | BN15-M1-E | 2.2 | 2.7 | -21.0 | 9.7 | 41.5 | 15.2 | 3.2 |
| BN15 | BN15-M1-F | 2.7 | 3.4 | -21.1 | 9.7 | 40.9 | 15.0 | 3.2 |
| BN15 | BN15-M1-G | 3.4 | 4.0 | -21.0 | 9.8 | 38.8 | 14.3 | 3.2 |
| BN15 | BN15-M1-H+I | 4.0 | 5.5 | -20.9 | 9.5 | 40.7 | 15.0 | 3.2 |
| BN15 | BN15-M1-J | 5.5 | 6.2 | -21.0 | 9.6 | 40.4 | 14.7 | 3.2 |
| BN15 | BN15-M1-K | 6.2 | 6.9 | -21.0 | 9.8 | 45.1 | 16.1 | 3.3 |
| BN15 | BN15-M1-L | 6.9 | 7.6 | -20.9 | 9.8 | 39.3 | 14.4 | 3.2 |
| BN15 | BN15-M1-M | 7.6 | 8.2 | -21.0 | 10.1 | 44.0 | 15.8 | 3.3 |
| BN15 | BN15-M1-N | 8.2 | 8.9 | -21.0 | 10.7 | 41.7 | 14.8 | 3.3 |
| BN15 | BN15-M1-O | 8.9 | 9.5 | -21.4 | 10.7 | 40.8 | 14.6 | 3.3 |
| BN124 | BN124-M1-A+B | 0.3 | 1.0 | -20.0 | 12.3 | 36.7 | 13.6 | 3.1 |
| BN124 | BN124-M1-C+D | 1.0 | 1.6 | -20.1 | 11.9 | 37.9 | 14.1 | 3.1 |
| BN124 | BN124-M1-E+F | 1.6 | 2.3 | -20.4 | 11.9 | 38.3 | 14.1 | 3.2 |
| BN124 | BN124-M1-G | 2.3 | 2.7 | -20.3 | 11.5 | 38.9 | 14.6 | 3.1 |
| BN124 | BN124-M1-H+I | 2.7 | 3.7 | -20.2 | 11.5 | 36.6 | 13.4 | 3.2 |
| BN124 | BN124-M1-J | 3.7 | 4.1 | -20.8 | 11.1 | 36.9 | 13.5 | 3.2 |
| BN124 | BN124-M1-K | 4.1 | 4.6 | -20.6 | 10.7 | 38.2 | 14.2 | 3.1 |
| BN124 | BN124-M1-L+M+N | 4.6 | 6.1 | -20.7 | 11.3 | 26.0 | 9.6 | 3.3 |
| BN124 | BN124-M1-O+P+Q+R+S+T+U | 6.1 | 9.3 | n.a. | n.a. | 7.0 | 2.5 | 3.2 |
| BN144 | BN144-M1-A+B+C | 0.3 | 1.6 | -20.3 | 11.7 | 40.7 | 15.0 | 3.2 |
| BN144 | BN144-M1-D | 1.6 | 2.1 | -20.2 | 10.4 | 39.9 | 14.7 | 3.1 |
| BN144 | BN144-M1-E | 2.1 | 2.5 | -20.2 | 10.1 | 38.8 | 14.4 | 3.1 |
| BN144 | BN144-M1-F | 2.5 | 3.0 | -20.3 | 9.9 | 39.8 | 14.6 | 3.2 |
| BN144 | BN144-M1-G | 3.0 | 3.6 | -20.5 | 9.8 | 40.0 | 14.6 | 3.2 |
| BN144 | BN144-M1-H | 3.6 | 4.2 | -20.4 | 9.6 | 39.0 | 14.5 | 3.1 |
| BN144 | BN144-M1-I | 4.2 | 4.9 | -20.4 | 9.8 | 39.4 | 14.4 | 3.2 |
| BN144 | BN144-M1-J | 4.9 | 5.5 | -20.4 | 10.1 | 39.1 | 14.3 | 3.2 |
| BN144 | BN144-M1-K | 5.5 | 6.2 | -20.3 | 10.5 | 40.0 | 14.3 | 3.3 |
| BN144 | BN144-M1-L | 6.2 | 6.9 | -20.3 | 10.6 | 41.5 | 15.1 | 3.2 |
| BN144 | BN144-M1-M+N | 6.9 | 8.1 | -20.2 | 10.8 | 40.7 | 14.8 | 3.2 |
| BN144 | BN144-M1-O+P | 8.1 | 9.3 | -20.8 | 11.3 | 37.6 | 13.3 | 3.3 |
| BN197a | BN197a-M1-A | 0.3 | 0.7 | -20.5 | 12.1 | 42.2 | 14.5 | 3.2 |
| BN197a | BN197a-M1-B | 0.7 | 1.1 | -20.7 | 11.8 | 40.8 | 14.9 | 3.2 |
| BN197a | BN197a-M1-C | 1.1 | 1.5 | -20.8 | 11.0 | 40.4 | 14.9 | 3.2 |
| BN197a | BN197a-M1-D | 1.5 | 1.9 | -20.8 | 11.1 | 41.5 | 15.1 | 3.2 |
| BN197a | BN197a-M1-E | 1.9 | 2.3 | -20.7 | 10.5 | 40.6 | 15.1 | 3.1 |
| BN197a | BN197a-M1-F+G | 2.3 | 3.1 | -20.4 | 11.2 | 40.7 | 14.9 | 3.2 |
| BN197a | BN197a-M1-H | 3.1 | 3.6 | -20.4 | 11.1 | 42.2 | 15.2 | 3.2 |
| BN197a | BN197a-M1-I | 3.6 | 4.2 | -20.3 | 10.7 | 39.0 | 14.3 | 3.2 |
| BN197a | BN197a-M1-J | 4.2 | 4.8 | -20.2 | 11.3 | 42.1 | 15.3 | 3.2 |
| BN197a | BN197a-M1-K | 4.8 | 5.4 | -20.1 | 11.7 | 41.3 | 15.2 | 3.2 |
| BN197a | BN197a-M1-L | 5.4 | 5.9 | -20.4 | 11.2 | 39.6 | 14.4 | 3.2 |
| BN197a | BN197a-M1-M | 5.9 | 6.5 | -20.5 | 10.9 | 41.4 | 14.8 | 3.3 |
| BN197a | BN197a-M1-N+O | 6.5 | 7.7 | -20.6 | 11.0 | 41.2 | 14.7 | 3.3 |
| BN197a | BN197a-M1-P | 7.7 | 8.2 | -20.6 | 11.1 | 41.8 | 14.9 | 3.3 |
| BN197a | BN197a-M1-Q | 8.2 | 8.7 | -20.4 | 11.7 | 41.8 | 15.0 | 3.3 |
| BN197a | BN197a-M1-R | 8.7 | 9.3 | -21.7 | 10.6 | 40.3 | 14.0 | 3.4 |
| BN213 | BN213-M1-A | 0.3 | 0.8 | -20.9 | 12.7 | 41.0 | 15.0 | 3.2 |
| BN213 | BN213-M1-B | 0.8 | 1.2 | -20.8 | 12.9 | 41.6 | 15.4 | 3.1 |
| BN213 | BN213-M1-C | 1.2 | 1.7 | -20.9 | 12.2 | 40.9 | 15.1 | 3.1 |
| BN213 | BN213-M1-D+E | 1.7 | 2.7 | -20.5 | 11.6 | 39.1 | 14.4 | 3.2 |
| BN213 | BN213-M1-F | 2.7 | 3.2 | -20.6 | 10.5 | 40.7 | 15.0 | 3.1 |
| BN213 | BN213-M1-G | 3.2 | 3.8 | -20.8 | 10.5 | 41.2 | 15.2 | 3.2 |
| BN213 | BN213-M1-H | 3.8 | 4.5 | -20.6 | 10.2 | 41.5 | 15.4 | 3.1 |
| BN213 | BN213-M1-I | 4.5 | 5.2 | -20.6 | 10.4 | 41.5 | 15.3 | 3.2 |
| BN213 | BN213-M1-J+K | 5.2 | 6.6 | -20.5 | 10.8 | 37.8 | 14.0 | 3.1 |
| BN213 | BN213-M1-L | 6.6 | 7.3 | -20.5 | 11.0 | 41.3 | 15.3 | 3.2 |
| BN213 | BN213-M1-M+N+O | 7.3 | 9.2 | -20.6 | 11.6 | 39.7 | 14.4 | 3.2 |

***S3.4 OsteoBioR Bayesian Modelling***

The OsteoBioR software allows for the modelling of temporal isotopic variability from length and isotopic measurements. Users must provide a matrix of values wherein each entry denotes the percentage of remodelling within skeletal materials. The OsteoBioR R code is available on the GitHub of the Pandora & IsoMemo software platform (<https://github.com/Pandora-IsoMemo/osteo-bior>). A user-friendly Shiny online interface is available online at: <https://isomemoapp.com/app/osteo-bio-r>. Matrix row values correspond to time intervals, whereas the columns correspond to isotopic measurements obtained from various parts of the skeleton. In our study, we employed an OsteoBioR model to reconstruct isotopic profiles from birth to roughly nine and a half years of age from isotopic measurements on tooth sections. The sectioning technique employed (refer to Supplementary Information S3.1) yields tooth segments that represent circa six-month periods. The age attributed to each segment was calculated based on the growth rates of individual teeth and the difference in growth rates across different tooth segments, as detailed in Supplementary Information S3.2. Under a Bayesian framework, employed by OsteoBioR model, the generated outputs allow for direct comparison of temporal variations in isotopic values for different individuals on the same time resolution. The model considers that the length of a segment might not precisely match the standard six-month period used in our analysis. Therefore, each segment is divided into columns that correspond to the number of six-month intervals it contains. The remodelling percentages in each cell of these columns reflect the portion of the segment pertaining to each time interval. For more details on the OsteBioR implementation see Cocozza et al. (2021)(32).

Figure S3.4.1, S3.4.2, S3.4.3, and S3.4.4 below present Bayesian modelled temporal plots for individuals from Pompeii, Ostia AVM, Bainesse, and Thessaloniki.

**Figure S3.4.1.** Bayesian temporal modelling of M1 incremental dentine *δ*^13^C and *δ*^15^N for individuals from Pompeii.


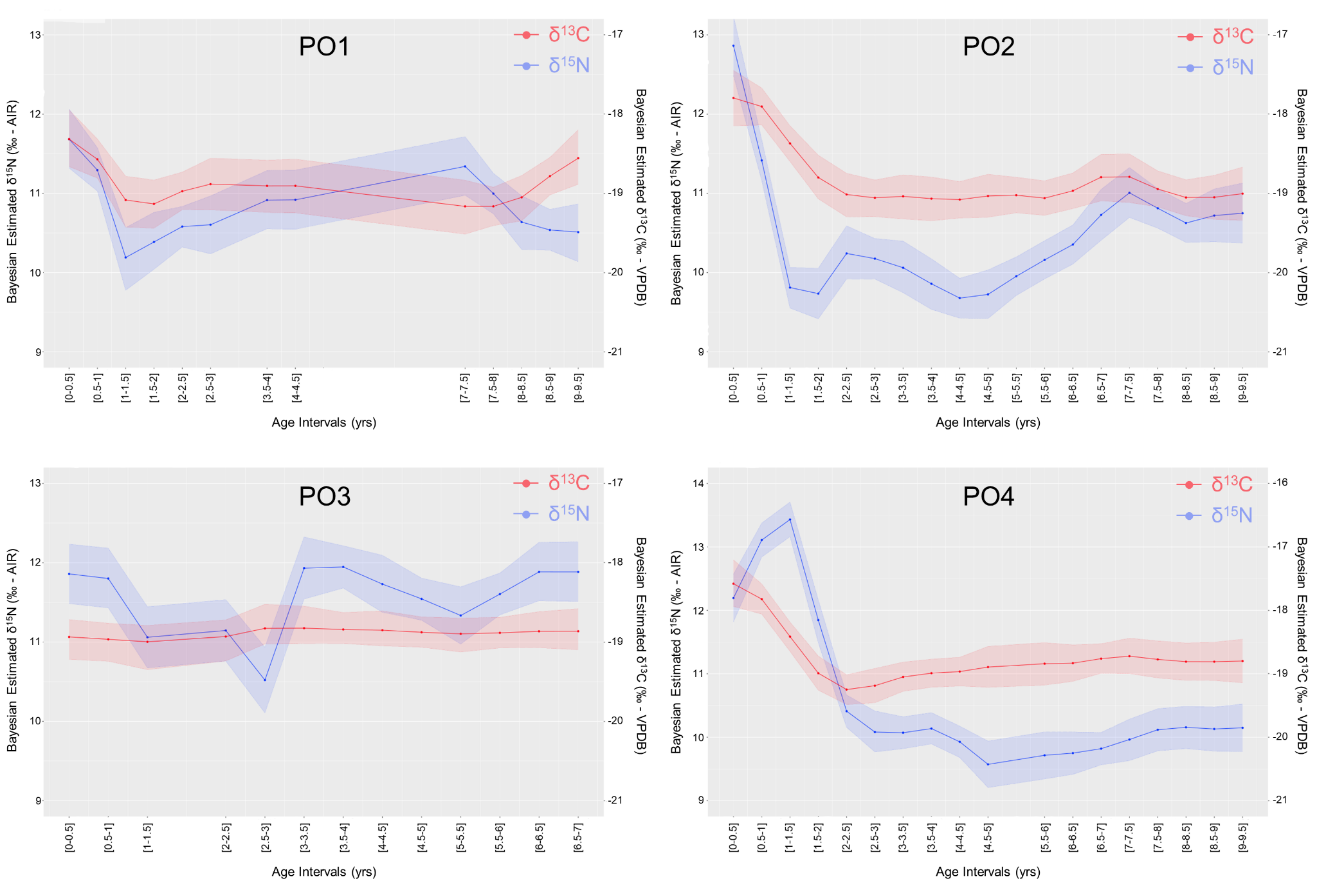


***Figure S3.4.2.*** *Bayesian temporal modelling of M1 incremental dentine δ^13^C and δ^15^N for individuals from Ostia AVM.*

**
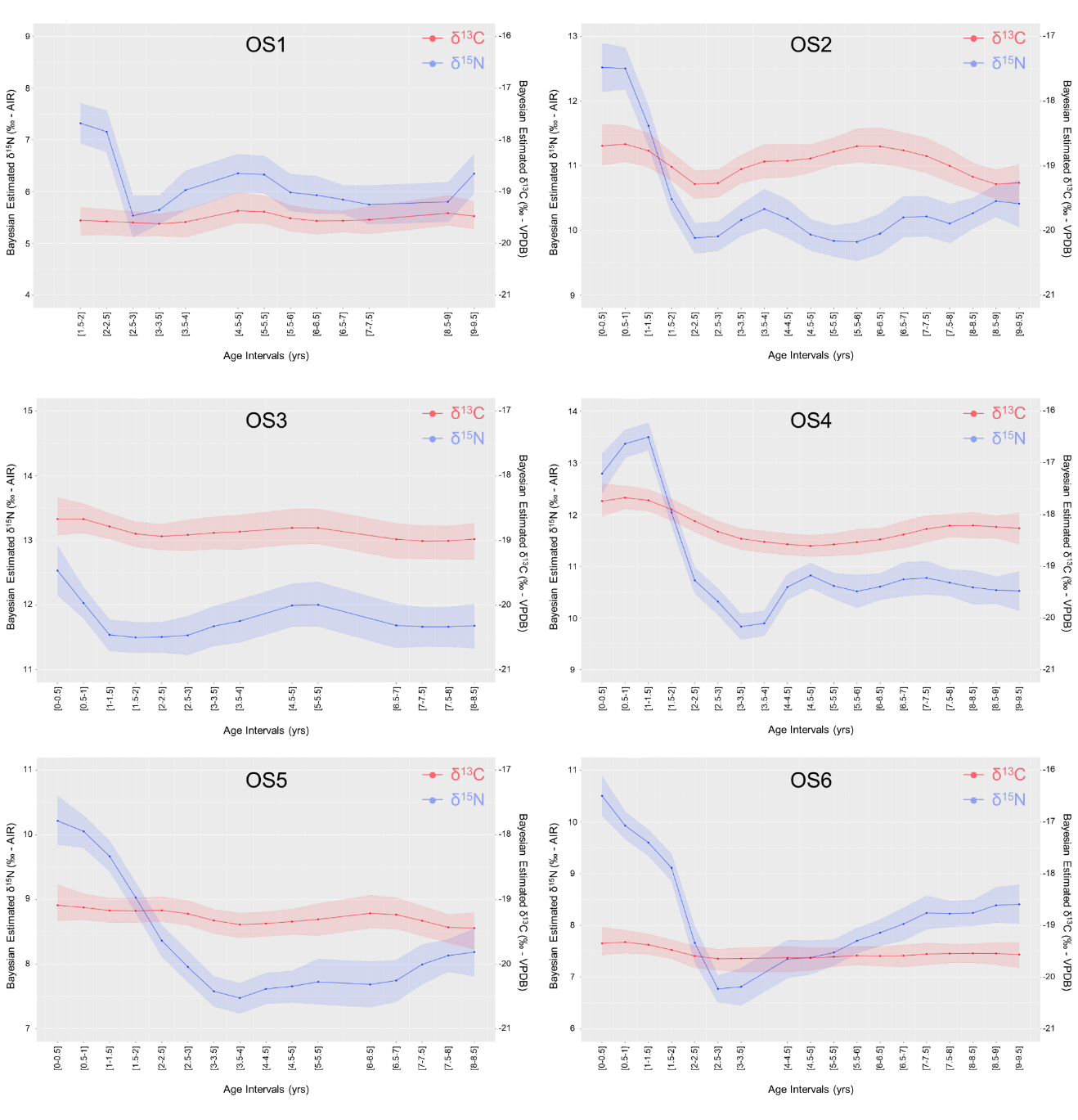
**

***Figure S3.4.3.*** *Bayesian temporal modelling of M1 incremental dentine δ^13^C and δ^15^N for individuals from Bainesse.*

**
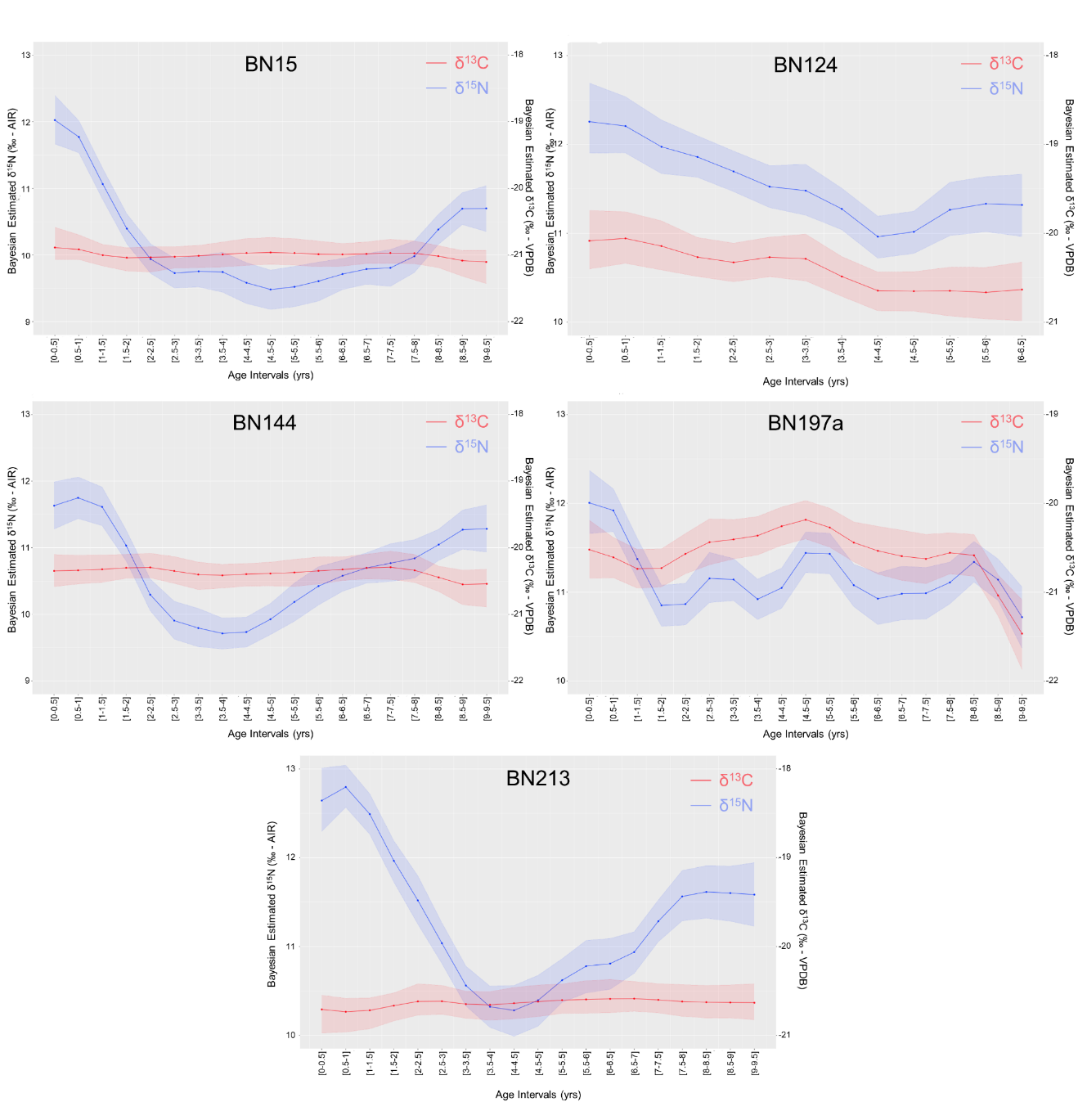
**

***Figure S3.4.4.*** *Bayesian temporal modelling of M1 incremental dentine δ^13^C and δ^15^N for individuals from Thessaloniki.*


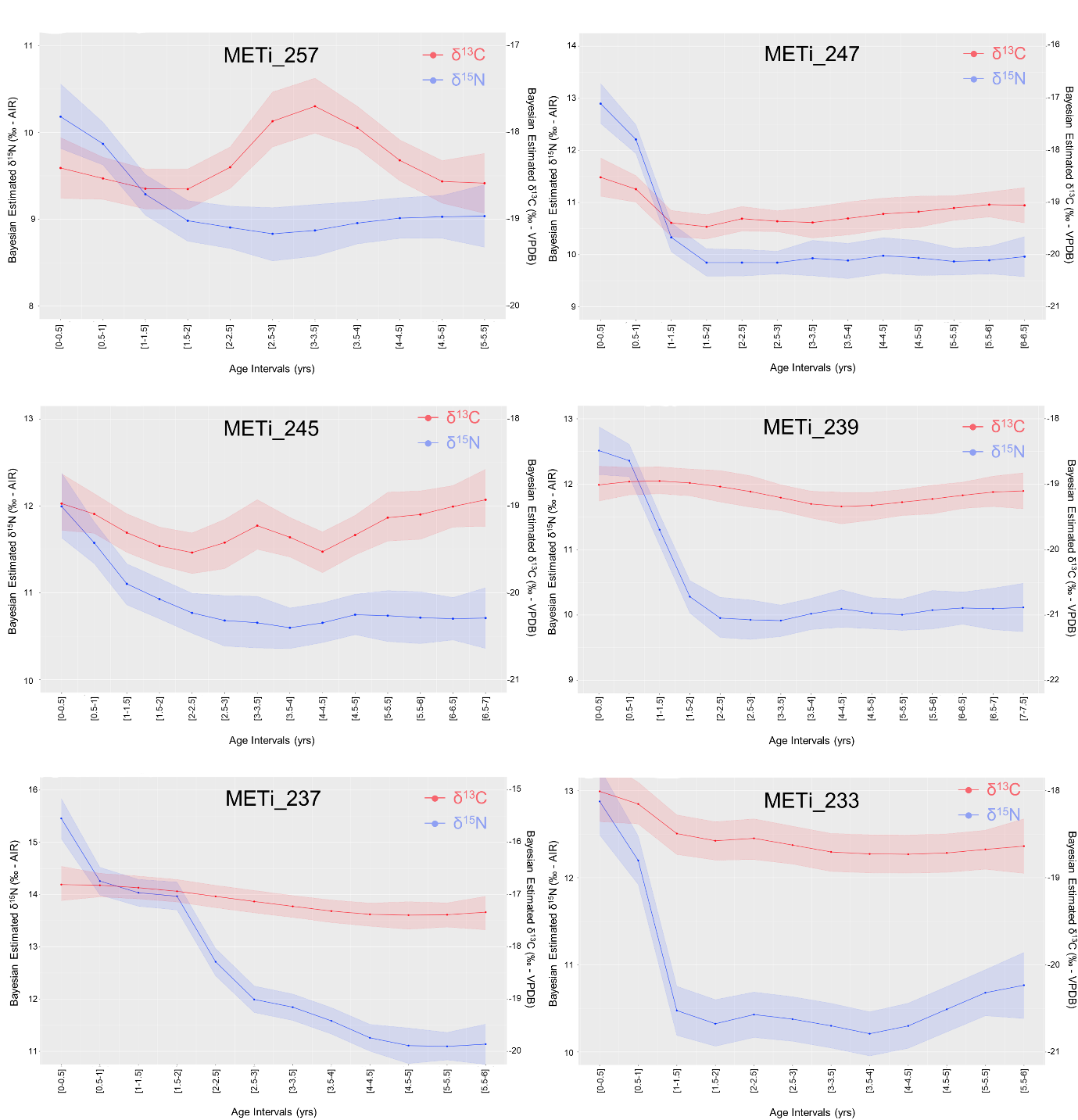


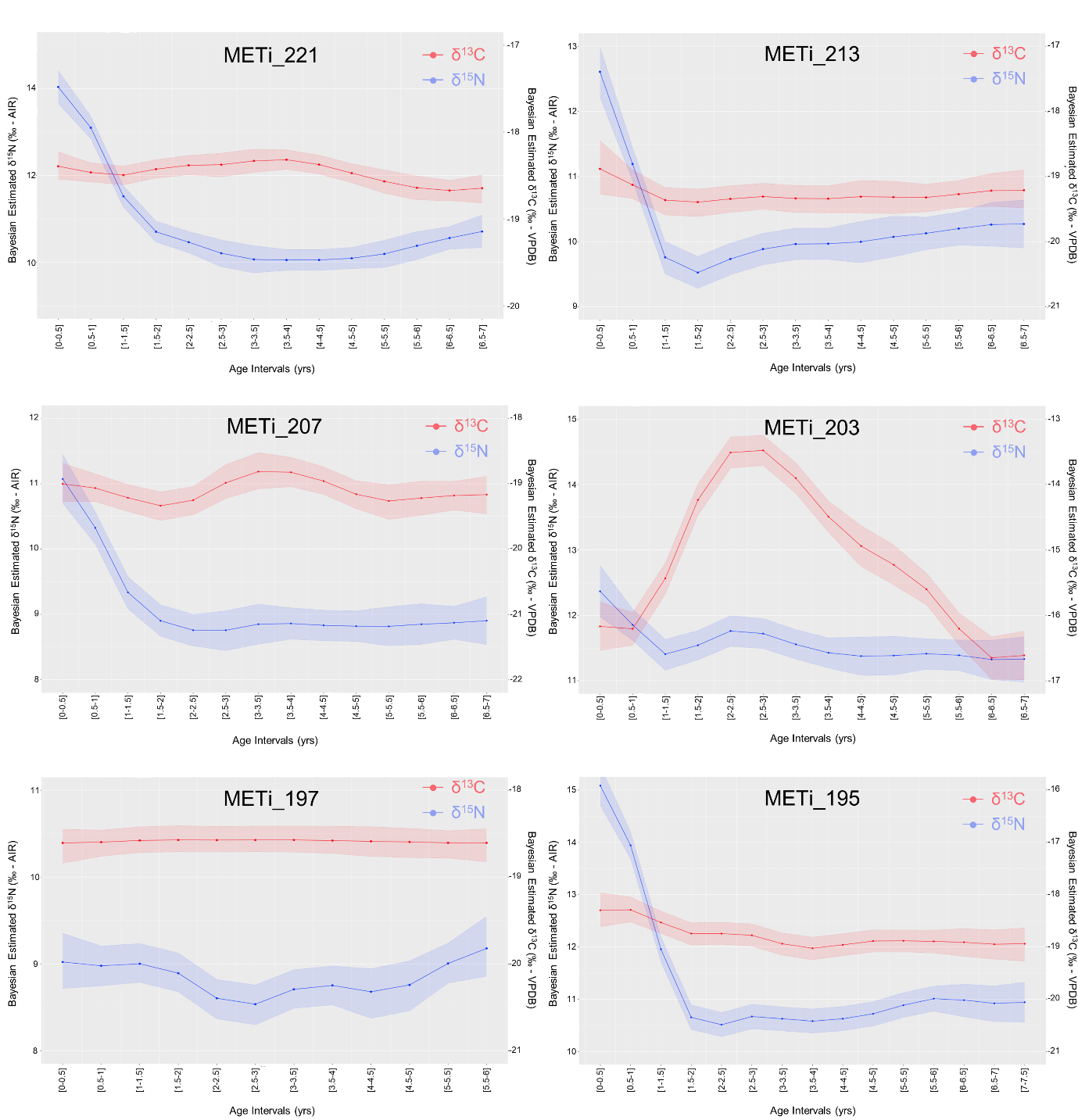


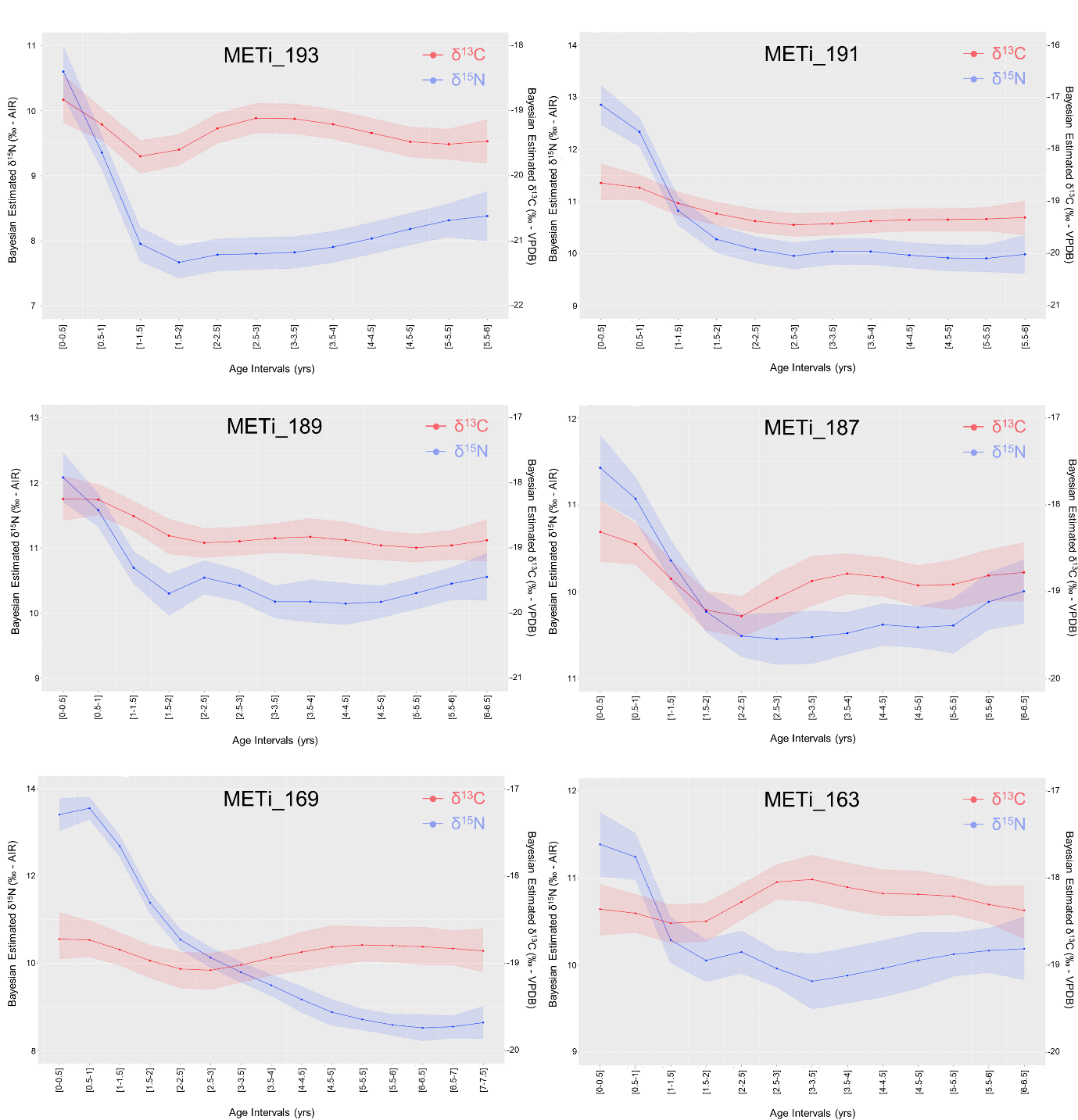


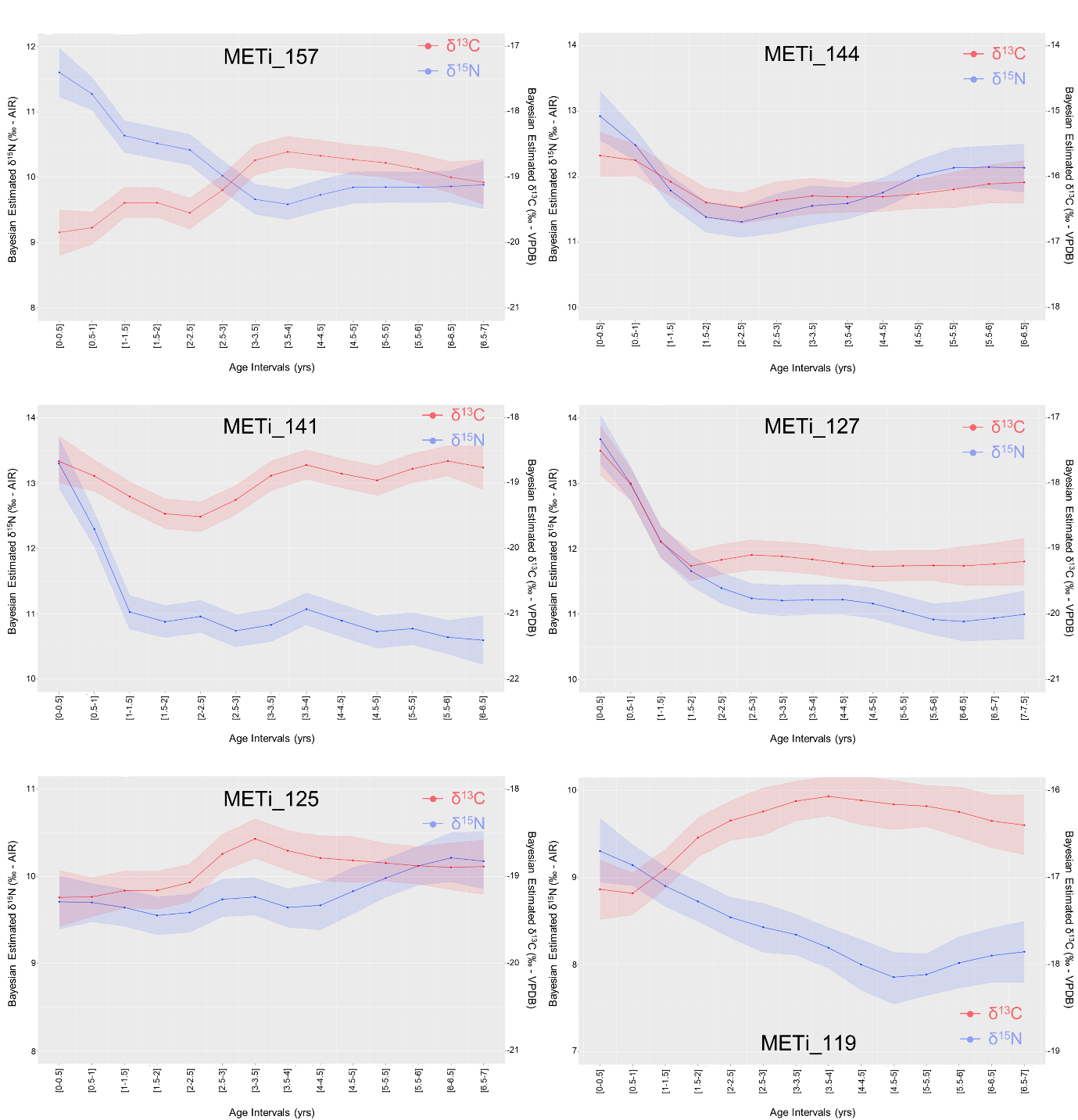


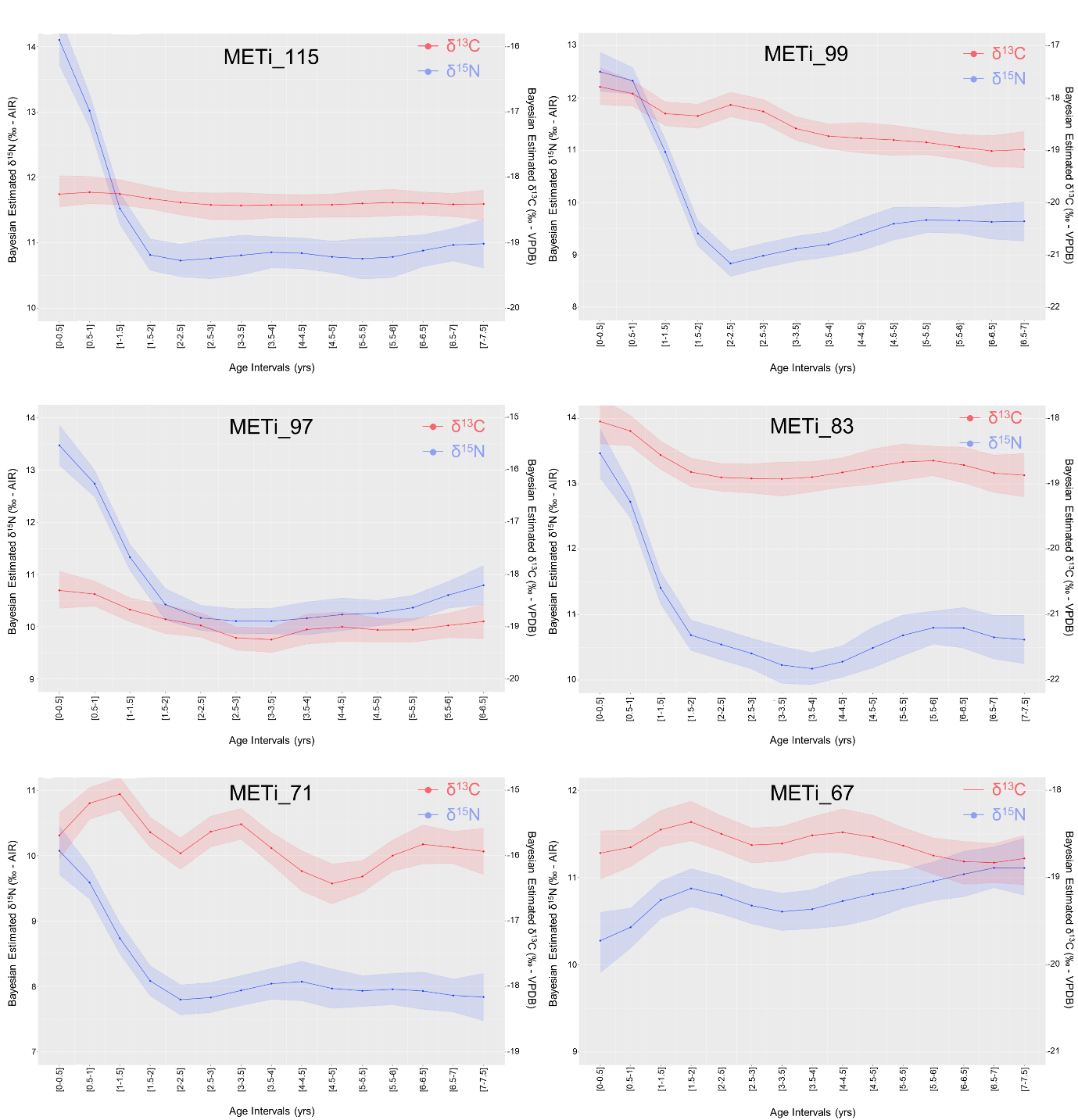


***S3.5 ChangeR model results***

Results from the ChangeR model are listed in table S3.5.1 and plotted in figure 3.5.2 below.

***Table S3.5.1.*** *Estimated breastfeeding cessation ages for individuals from Ostia AVM, Pompeii, Bainesse, and Thessaloniki at 68% and 95% credibility range. Breastfeeding cessation ages for OS1, METi_197, METi_125, and METi_67 could not be estimated.*

| **Sample ID** | **Site** | **Biological Sex** | **Min. Breastfeeding Cessation Age (68% - Years)** | **Max. Breastfeeding Cessation Age (68% - Years)** | **Max. Breastfeeding Cessation Age (95%- Years)** | **Max. Breastfeeding Cessation Age (95%- Years)** |
| --- | --- | --- | --- | --- | --- | --- |
| OS1 | Ostia AVM | M | - | - | - | - |
| OS2 | Ostia AVM | F | 2.14 | 2.44 | 1.98 | 2.71 |
| OS3 | Ostia AVM | M | 0.80 | 1.36 | 0.41 | 1.55 |
| OS4 | Ostia AVM | F | 2.65 | 3.27 | 2.34 | 3.61 |
| OS5 | Ostia AVM | ? | 3.23 | 3.51 | 3.07 | 3.66 |
| OS6 | Ostia AVM | ?F | 2.72 | 2.98 | 2.59 | 3.13 |
| PO1 | Pompeii | M | 0.71 | 1.38 | 0.34 | 1.65 |
| PO2 | Pompeii | M | 1.12 | 1.37 | 1.04 | 1.54 |
| PO3 | Pompeii | F | 0.26 | 1.08 | 0.25 | 1.60 |
| PO4 | Pompeii | F | 2.79 | 3.98 | 2.31 | 4.89 |
| BN15 | Bainesse | M | 2.52 | 3.20 | 2.18 | 3.8 |
| BN124 | Bainesse | ? | 4.17 | 4.54 | 3.83 | 4.83 |
| BN144 | Bainesse | ?F | 3.14 | 3.58 | 2.93 | 3.80 |
| BN197a | Bainesse | M | 1.36 | 1.90 | 0.98 | 2.14 |
| BN213 | Bainesse | F | 3.72 | 4.06 | 3.51 | 4.21 |
| METi_257 | Thessaloniki | M | 1.79 | 2.01 | 1.58 | 2.17 |
| METi_247 | Thessaloniki | F | 1.44 | 1.64 | 1.35 | 1.78 |
| METi_245 | Thessaloniki | F | 1.85 | 2.15 | 1.62 | 2.47 |
| METi_239 | Thessaloniki | F | 1.95 | 2.24 | 1.81 | 2.40 |
| METi_237 | Thessaloniki | ?F | 2.95 | 4.04 | 2.50 | 4.67 |
| METi_223 | Thessaloniki | F | 1.29 | 1.58 | 1.13 | 1.90 |
| METi_221 | Thessaloniki | F | 1.81 | 2.15 | 1.60 | 2.6 |
| METi_213 | Thessaloniki | - | 1.27 | 1.33 | 1.24 | 1.37 |
| METi_207 | Thessaloniki | F | 1.53 | 1.64 | 1.48 | 1.70 |
| METi_203 | Thessaloniki | F | 0.45 | 1.05 | 0.40 | 1.27 |
| METi_197 | Thessaloniki | M | - | - | - | - |
| METi_195 | Thessaloniki | M | 1.73 | 1.86 | 1.66 | 1.93 |
| METi_193 | Thessaloniki | F | 1.37 | 1.44 | 1.34 | 1.49 |
| METi_191 | Thessaloniki | ?M | 1.65 | 1.96 | 1.50 | 2.14 |
| METi_189 | Thessaloniki | F | 1.43 | 1.82 | 1.18 | 2.09 |
| METi_187 | Thessaloniki | M | 2.00 | 2.30 | 1.87 | 2.48 |
| METi_169 | Thessaloniki | ?F | 3.08 | 4.16 | 2.62 | 4.89 |
| METi_163 | Thessaloniki | M | 1.50 | 2.00 | 1.29 | 2.44 |
| METi_157 | Thessaloniki | M | 3.09 | 3.45 | 2.85 | 3.67 |
| METi_144 | Thessaloniki | M | 1.73 | 1.94 | 1.57 | 2.03 |
| METi_141 | Thessaloniki | M | 1.20 | 1.35 | 1.10 | 1.42 |
| METi_127 | Thessaloniki | F | 1.83 | 2.00 | 1.63 | 2.12 |
| METi_125 | Thessaloniki | M | - | - | - | - |
| METi_119 | Thessaloniki | ?M | 4.55 | 4.84 | 4.36 | 4.99 |
| METi_115 | Thessaloniki | M | 1.53 | 1.62 | 1.50 | 1.67 |
| METi_99 | Thessaloniki | F | 1.96 | 2.26 | 1.78 | 2.47 |
| METi_97 | Thessaloniki | F | 1.88 | 2.07 | 1.78 | 2.27 |
| METi_83 | Thessaloniki | F | 1.79 | 2.05 | 1.54 | 2.22 |
| METi_71 | Thessaloniki | M | 1.78 | 1.97 | 1.61 | 2.06 |
| METi_67 | Thessaloniki | M | - | - | - | - |

***Figure S3.5.2.*** *Estimated breastfeeding cessation ages for individuals from Ostia AVM, Pompeii, Bainesse, and Thessaloniki at 68% and 95% credibility range.*


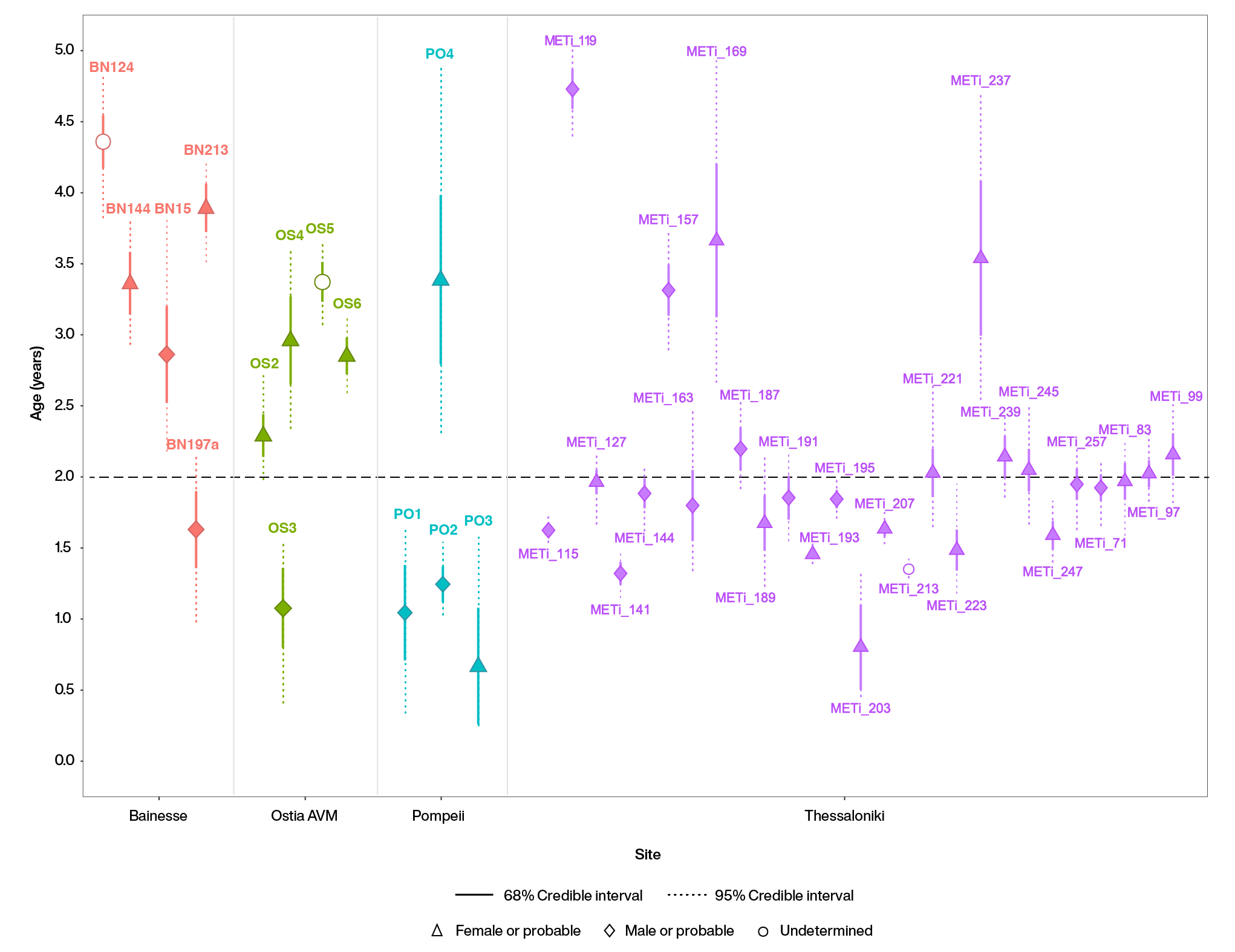


***S3.6 Bayesian mixing model ReSources***

ReSources is a software designed to implement Bayesian mixing models for isotopic-based dietary reconstruction(41–45). This software is an enhanced version of the Bayesian software FRUITS. The ReSources R code is available on the GitHub of the Pandora & IsoMemo software platform (<https://github.com/Pandora-IsoMemo/resources>). A user-friendly Shiny online interface is available online at: <https://isomemoapp.com/app/resources>. We applied this model to estimate the protein contribution of different food sources during the childhood diets of individuals from the 1^st^ century CE Pompeii and Ostia Via del Mare. We modelled each individual separately. We also implemented a random effects structure on the categorical covariate "Age," with each level indicating the designated age for each interval(44). This approach differs from models without covariates in that the Dirichlet prior values of the source contribution parameters ("alpha") are variable, originating from a distribution with a common mean and standard deviation for each age group and food source category.

The primary food groups considered were human milk, C_3_ plants (i.e. wheat, barley, rye, legumes, fruit, and vegetables), terrestrial animals, C_4_ cereals (i.e. millet and sorghum), and marine resources. We generated independent estimates of the protein contributions from these groups for each isotopic value (*δ*^13^C, *δ*^15^N) obtained from isotopic modelled values by the software OsteoBioR(32). For terrestrial fauna and C_3_ plants, we relied on available data from Pompeii(46) and Portus(47). In the absence of isotopic measurements for C_4_ cereals from these sites, we referenced values reported for the Bronze Age in Greece by Nitsch et al.(48). Despite the chronological differences, Greece's temperate Mediterranean climate and environment bear similarities to those of southern-central Italy, making these values the closest available. For marine foods, we utilised isotopic measurements from Roman sites along the Tyrrhenian Sea, namely Pompeii(46), Portus(47), and Velia(49), to establish our baseline. The *δ*^13^C and *δ*^15^N values for the food remain are detailed below in Table S3.6.1. In modelling human milk's isotopic reference, we considered modelled values generated by OsteoBioR(32) for the first interval (i.e. [0-0.5]) independently for each individual (Table S3.6.2). Individual OS1 was not investigated due to the low level of preservation of the sample.

***Table S3.6.1.*** *Isotopic values for food remain employed in Bayesian dietary modelling. These do not include corrections for offsets between edible tissues and food remains (e.g. muscle meat protein vs. bone collagen collagen). Corrected values are given in table S3.6.3.*

|  | ***δ*^13^C**  **(‰ - VPDB)** |  | ***δ*^15^N**  **(‰ - AIR)** |  |
| --- | --- | --- | --- | --- |
|  | **Mean** | **Unc.** | **Mean** | **Unc.** |
| **Marine resources bone collagen** | -13.01 | 2.24 | 9.53 | 2.18 |
| **Charred C4 Cereals remains** | -10.53 | 0.27 | 6.50 | 2.74 |
| **Charred C3 Plant remains** | -23.18 | 0.87 | 8.53 | 3.11 |
| **Terrestrial Animals bone collagen** | -20.69 | 0.73 | 5.81 | 2.27 |

***Table S3.6.2.*** *Isotopic values for human milk employed in Bayesian dietary modelling for each individual. These do not include corrections for offsets between milk and human dentine collagen. Corrected values are given in table S3.6.4.*

|  | ***δ*^13^C**  **(‰ - VPDB)** |  | ***δ*^15^N**  **(‰ - AIR)** |  |
| --- | --- | --- | --- | --- |
|  | **Mean** | **Unc.** | **Mean** | **Unc.** |
| **OS1** | - | - | - | - |
| **OS2** | -18.69 | 0.5 | 12.52 | 0.5 |
| **OS3** | -18.66 | 0.5 | 12.53 | 0.5 |
| **OS4** | -17.73 | 0.5 | 12.79 | 0.5 |
| **OS5** | -19.08 | 0.5 | 10.22 | 0.5 |
| **OS6** | -19.34 | 0.5 | 10.51 | 0.5 |
| **PO1** | -18.31 | 0.5 | 11.69 | 0.5 |
| **PO2** | -17.8 | 0.5 | 12.86 | 0.5 |
| **PO3** | -18.94 | 0.5 | 11.86 | 0.5 |
| **PO4** | -17.58 | 0.5 | 12.19 | 0.5 |

We applied offsets between archaeological food remains and the actual edible food components. These adjustments draw from the work of Fernandes et al.(42), with updates by Soncin et al.(50). For plants, the corrections applied were *Δ*^13^C_protein-bulk=-2‰; *Δ*^15^N_protein-bulk=0‰. For terrestrial animals, the adjustments were *Δ*^13^C_protein-collagen=-2‰; *Δ*^15^N_protein-collagen=0‰. For marine fish, the corrections were *Δ*^13^C_protein-collagen = -1‰ and *Δ*^15^N_protein-collagen=+1.5‰. Adjusted isotopic values used for each source are detailed in Table S3.6.3. For human milk, we implemented the following corrections: *Δ*^13^C_protein-bulk=-5‰; *Δ*^15^N_protein-bulk=-5.5‰(43). Corrected values for each individual are presented in Table S3.6.4.

***Table S3.6.3.*** *Corrected isotopic food values as employed in dietary modelling.*

|  | ***δ*^13^C_corrected_**  **(‰ - VPDB)** |  | ***δ*^15^N_corrected_**  **(‰ - AIR)** |  |
| --- | --- | --- | --- | --- |
|  | **Mean** | **Unc.** | **Mean** | **Unc.** |
| **Marine Resources** | -14.01 | 2.24 | 11.03 | 2.18 |
| **C4 Cereals** | -12.53 | 0.27 | 6.50 | 2.74 |
| **C3 Plants** | -25.18 | 0.87 | 8.53 | 3.11 |
| **Terrestrial Animals** | -22.69 | 0.73 | 5.81 | 2.27 |

***Table S3.6.4.*** *Corrected isotopic human milk values as employed in dietary modelling.*

|  | ***δ*^13^C_corrected_**  **(‰ - VPDB)** |  | ***δ*^15^N_corrected_**  **(‰ - AIR)** |  |
| --- | --- | --- | --- | --- |
|  | **Mean** | **Unc.** | **Mean** | **Unc.** |
| **OS1** | - | - | - | - |
| **OS2** | -23.69 | 0.5 | 7.02 | 0.5 |
| **OS3** | -23.66 | 0.5 | 7.03 | 0.5 |
| **OS4** | -22.73 | 0.5 | 7.29 | 0.5 |
| **OS5** | -24.08 | 0.5 | 4.72 | 0.5 |
| **OS6** | -24.34 | 0.5 | 5.01 | 0.5 |
| **PO1** | -23.31 | 0.5 | 6.19 | 0.5 |
| **PO2** | -22.8 | 0.5 | 7.36 | 0.5 |
| **PO3** | -23.94 | 0.5 | 6.36 | 0.5 |
| **PO4** | -22.58 | 0.5 | 5.69 | 0.5 |

The study by Fernandes et al.(42) provides protein concentration values, albeit we doubled their uncertainty: for C_4_ cereals, the values are 10±5%; for terrestrial animals, the values are 30±5%; for marine resources, the concentration is reported as 65±10%. In the context of C_3_ plants, particularly with the addition of legumes, a higher protein concentration of 15±10% was determined. For human milk, we calculated a conservative protein concentration of 10±5%, considering its variability throughout the lactation stage(51–53).

Our Bayesian mixing model also incorporated isotopic offsets between dietary protein and human tissue composition, alongside dietary routing mechanisms(42, 43, 54). Specifically, *δ*^15^N values of dentine collagen were considered to entirely reflect the dietary protein intake, although with an isotopic offset set at 5.5±0.5‰(42). Meanwhile, for *δ*^13^C in dentine collagen, an offset of 5±2.3‰ was established relative to the food sources(43). Fernandes(43) demonstrated the validity of such an offset for simpler Bayesian mixing models aiming at protein estimates, despite its large *δ*^13^C uncertainty.

To enhance the accuracy of dietary estimates and ensure that human milk consumption is not erroneously accounted for as a food source post-weaning, we incorporated prior constraints into our model(44, 45). Guided by both contemporary and historical medical advice(1–3, 55–58), we initially posited that infants rely solely on human milk for nourishment during the first six months of life. We acknowledged the possibility that breastfeeding might stop slightly earlier than this period by adjusting our model so that, in the earliest phase, human milk contributed (only) more than 80% of the infant's protein intake. We then progressively adjusted our priors for subsequent intervals to gradually decrease the proportion of human milk in the diet, until breastfeeding was fully stopped, grounding on the results of the ChangeR algorithm. From this interval through to the final age considered in the model, the contribution of human milk to the diet was set at 0%. Due to the impossibility of clearly separating estimates from C_3_ plants and C_3_ animal products and the lack of additional information necessary for imposing priors, we added estimates from these two groups to display a combined C_3_ estimate.

Final estimates are presented and summarised in the table S3.6.5 below and in the main text of this study in Figure 2b.

***Tab. S3.6.5.*** *Dietary estimates (95% credibility interval) for each age interval for each individual from Pompeii and Ostia AVM according to four main food sources (C_3_ Sources; Marine; C_4_ Cereals; Human Milk).*

| **Isotopic**  **ID** | **Age**  **Interval**  **(years)** | **C_3_**  **Sources**  **Min.**  **(%)** | **C_3_**  **Sources**  **Max.**  **(%)** | **Marine**  **Min.**  **(%)** | **Marine**  **Max.**  **(%)** | **C_4_**  **Cereals**  **Min.**  **(%)** | **C_4_**  **Cereals**  **Max.**  **(%)** | **Human**  **Milk**  **Min.**  **(%)** | **Human**  **Milk**  **Max.**  **(%)** |
| --- | --- | --- | --- | --- | --- | --- | --- | --- | --- |
| OS2 | 0-0.5 | 0 | 1.8 | 0 | 0.5 | 0 | 0.3 | 97.5 | 100 |
| OS2 | 0.5-1 | 0 | 8.3 | 0 | 1.9 | 0 | 4.5 | 88.6 | 100 |
| OS2 | 1-1.5 | 0 | 99.6 | 0 | 2 | 0 | 15.3 | 0 | 100 |
| OS2 | 1.5-2 | 18.3 | 100 | 0 | 1.1 | 0 | 11.9 | 0 | 80.1 |
| OS2 | 2-2.5 | 88.6 | 100 | 0 | 1.1 | 0 | 10.1 | 0 | 0 |
| OS2 | 2.5-3 | 91.9 | 100 | 0 | 1.2 | 0 | 7.2 | 0 | 0 |
| OS2 | 3-3.5 | 88.4 | 100 | 0 | 2.8 | 0 | 7.2 | 0 | 0 |
| OS2 | 3.5-4 | 83.3 | 100 | 0 | 2.7 | 0 | 13.6 | 0 | 0 |
| OS2 | 4-4.5 | 83.2 | 100 | 0 | 2.6 | 0 | 16 | 0 | 0 |
| OS2 | 4.5-5 | 86.7 | 100 | 0 | 2.7 | 0 | 13.1 | 0 | 0 |
| OS2 | 5-5.5 | 84.8 | 100 | 0 | 2.5 | 0 | 14.6 | 0 | 0 |
| OS2 | 5.5-6 | 86.9 | 100 | 0 | 3.3 | 0 | 11.8 | 0 | 0 |
| OS2 | 6-6.5 | 81.5 | 100 | 0 | 2.5 | 0 | 16.3 | 0 | 0 |
| OS2 | 6.5-7 | 79.4 | 100 | 0 | 3.2 | 0 | 20.6 | 0 | 0 |
| OS2 | 7-7.5 | 89.5 | 100 | 0 | 2.9 | 0 | 10 | 0 | 0 |
| OS2 | 7.5-8 | 84.6 | 100 | 0 | 1.6 | 0 | 15.2 | 0 | 0 |
| OS2 | 8-8.5 | 87.4 | 100 | 0 | 1.6 | 0 | 12.3 | 0 | 0 |
| OS2 | 8.5-9 | 91.3 | 100 | 0 | 0.9 | 0 | 7.1 | 0 | 0 |
| OS2 | 9-9.5 | 87.2 | 100 | 0 | 1.5 | 0 | 11.2 | 0 | 0 |
| OS3 | 0-0.5 | 0 | 100 | 0 | 2 | 0 | 15.1 | 0 | 100 |
| OS3 | 0.5-1 | 0 | 100 | 0 | 3 | 0 | 19 | 0 | 99.8 |
| OS3 | 1-1.5 | 80.9 | 100 | 0 | 1.9 | 0 | 18.9 | 0 | 0 |
| OS3 | 1.5-2 | 85.7 | 100 | 0 | 1.9 | 0 | 14.3 | 0 | 0 |
| OS3 | 2-2.5 | 88.8 | 100 | 0 | 1.9 | 0 | 11 | 0 | 0 |
| OS3 | 2.5-3 | 85 | 100 | 0 | 1.7 | 0 | 15 | 0 | 0 |
| OS3 | 3-3.5 | 83.5 | 100 | 0 | 2.2 | 0 | 15.7 | 0 | 0 |
| OS3 | 3.5-4 | 87.8 | 100 | 0 | 1.7 | 0 | 12 | 0 | 0 |
| OS3 | 4-4.5 | - | - | - | - | - | - | - | - |
| OS3 | 4.5-5 | 83.5 | 100 | 0 | 2.4 | 0 | 15.9 | 0 | 0 |
| OS3 | 5-5.5 | 80.1 | 100 | 0 | 2.9 | 0 | 19.3 | 0 | 0 |
| OS3 | 5.5-6 | - | - | - | - | - | - | - | - |
| OS3 | 6-6.5 | - | - | - | - | - | - | - | - |
| OS3 | 6.5-7 | 88.1 | 100 | 0 | 1.5 | 0 | 11.6 | 0 | 0 |
| OS3 | 7-7.5 | 86.8 | 100 | 0 | 1.8 | 0 | 12.8 | 0 | 0 |
| OS3 | 7.5-8 | 82.9 | 100 | 0 | 2 | 0 | 16.9 | 0 | 0 |
| OS3 | 8-8.5 | 88.9 | 100 | 0 | 1.6 | 0 | 11 | 0 | 0 |
| OS3 | 8.5-9 | - | - | - | - | - | - | - | - |
| OS3 | 9-9.5 | - | - | - | - | - | - | - | - |
| OS4 | 0-0.5 | 0 | 0.1 | 0 | 0.00 | 0 | 0 | 99.7 | 100 |
| OS4 | 0.5-1 | 0 | 1.1 | 0 | 0.60 | 0 | 0.6 | 98.1 | 100 |
| OS4 | 1-1.5 | 0 | 4 | 0 | 2.30 | 0 | 4.4 | 93.5 | 100 |
| OS4 | 1.5-2 | 0 | 95.7 | 0 | 0.60 | 0 | 16.2 | 2.2 | 99.9 |
| OS4 | 2-2.5 | 20.7 | 100 | 0 | 2.40 | 0 | 14.3 | 0 | 77.4 |
| OS4 | 2.5-3 | 77.8 | 100 | 0 | 1.50 | 0 | 11.3 | 0 | 7.7 |
| OS4 | 3-3.5 | 90.5 | 100 | 0 | 1.20 | 0 | 8.5 | 0 | 0 |
| OS4 | 3.5-4 | 91.1 | 100 | 0 | 1.80 | 0 | 8.7 | 0 | 0 |
| OS4 | 4-4.5 | 88.8 | 100 | 0 | 1.20 | 0 | 10.9 | 0 | 0 |
| OS4 | 4.5-5 | 89.7 | 100 | 0 | 1.50 | 0 | 9.3 | 0 | 0 |
| OS4 | 5-5.5 | 89.9 | 100 | 0 | 1.20 | 0 | 8.5 | 0 | 0 |
| OS4 | 5.5-6 | 89.6 | 100 | 0 | 1.60 | 0 | 10.4 | 0 | 0 |
| OS4 | 6-6.5 | 85.1 | 100 | 0 | 1.80 | 0 | 12.7 | 0 | 0 |
| OS4 | 6.5-7 | 85 | 100 | 0 | 1.40 | 0 | 12.4 | 0 | 0 |
| OS4 | 7-7.5 | 82.8 | 100 | 0 | 2.60 | 0 | 17.1 | 0 | 0 |
| OS4 | 7.5-8 | 79.2 | 100 | 0 | 3.40 | 0 | 15.5 | 0 | 0 |
| OS4 | 8-8.5 | 85.6 | 100 | 0 | 2.60 | 0 | 14.3 | 0 | 0 |
| OS4 | 8.5-9 | 86.2 | 100 | 0 | 1.90 | 0 | 13.3 | 0 | 0 |
| OS4 | 9-9.5 | 87.6 | 100 | 0 | 1.70 | 0 | 12.4 | 0 | 0 |
| OS5 | 0-0.5 | 0 | 1.2 | 0 | 0.3 | 0 | 0.7 | 98 | 100 |
| OS5 | 0.5-1 | 0 | 9 | 0 | 0.7 | 0 | 5.9 | 86.8 | 100 |
| OS5 | 1-1.5 | 0 | 71.1 | 0 | 1 | 0 | 34.4 | 0.1 | 100 |
| OS5 | 1.5-2 | 0 | 100 | 0 | 1.8 | 0 | 23.1 | 0 | 99.3 |
| OS5 | 2-2.5 | 14.2 | 100 | 0 | 3 | 0 | 12.9 | 0 | 85.3 |
| OS5 | 2.5-3 | 68.7 | 100 | 0 | 2.1 | 0 | 9.7 | 0 | 26.4 |
| OS5 | 3-3.5 | 92.7 | 100 | 0 | 1.8 | 0 | 7 | 0 | 0 |
| OS5 | 3.5-4 | 91.7 | 100 | 0 | 1.4 | 0 | 7.8 | 0 | 0 |
| OS5 | 4-4.5 | 93.2 | 100 | 0 | 1.9 | 0 | 6.5 | 0 | 0 |
| OS5 | 4.5-5 | 91.6 | 100 | 0 | 1.7 | 0 | 7.5 | 0 | 0 |
| OS5 | 5-5.5 | 89.8 | 100 | 0 | 2.1 | 0 | 10 | 0 | 0 |
| OS5 | 5.5-6 | - | - | - | - | - | - | - | - |
| OS5 | 6-6.5 | 92 | 100 | 0 | 2.1 | 0 | 7.9 | 0 | 0 |
| OS5 | 6.5-7 | 92 | 100 | 0 | 2.1 | 0 | 7.4 | 0 | 0 |
| OS5 | 7-7.5 | 89.4 | 100 | 0 | 1.6 | 0 | 10.5 | 0 | 0 |
| OS5 | 7.5-8 | 90 | 100 | 0 | 1.6 | 0 | 9.8 | 0 | 0 |
| OS5 | 8-8.5 | 88.6 | 100 | 0 | 1.6 | 0 | 11.4 | 0 | 0 |
| OS5 | 8.5-9 | - | - | - | - | - | - | - | - |
| OS5 | 9-9.5 | - | - | - | - | - | - | - | - |
| OS6 | 0-0.5 | 0 | 1 | 0 | 0.4 | 0 | 0.4 | 98 | 100 |
| OS6 | 0.5-1 | 0 | 9.2 | 0 | 1 | 0 | 4.2 | 89.4 | 100 |
| OS6 | 1-1.5 | 0 | 70.8 | 0 | 1.3 | 0 | 16.2 | 2.4 | 100 |
| OS6 | 1.5-2 | 0 | 100 | 0 | 1.6 | 0 | 27.9 | 0 | 99.1 |
| OS6 | 2-2.5 | 42.8 | 100 | 0 | 1.7 | 0 | 8.6 | 0 | 56.5 |
| OS6 | 2.5-3 | 92.5 | 100 | 0 | 1.3 | 0 | 7.4 | 0 | 0 |
| OS6 | 3-3.5 | 95.9 | 100 | 0 | 1.3 | 0 | 4.1 | 0 | 0 |
| OS6 | 3.5-4 | - | - | - | - | - | - | - | - |
| OS6 | 4-4.5 | 92.4 | 100 | 0 | 1.4 | 0 | 7.4 | 0 | 0 |
| OS6 | 4.5-5 | 92.5 | 100 | 0 | 1.2 | 0 | 7.4 | 0 | 0 |
| OS6 | 5-5.5 | 93.3 | 100 | 0 | 1.7 | 0 | 5.7 | 0 | 0 |
| OS6 | 5.5-6 | 92.1 | 100 | 0 | 2 | 0 | 7.4 | 0 | 0 |
| OS6 | 6-6.5 | 89 | 100 | 0 | 1.3 | 0 | 10.9 | 0 | 0 |
| OS6 | 6.5-7 | 88 | 100 | 0 | 1.8 | 0 | 12 | 0 | 0 |
| OS6 | 7-7.5 | 85 | 100 | 0 | 2.1 | 0 | 15 | 0 | 0 |
| OS6 | 7.5-8 | 85.6 | 100 | 0 | 1.9 | 0 | 14.3 | 0 | 0 |
| OS6 | 8-8.5 | 86.4 | 100 | 0 | 2.3 | 0 | 13.6 | 0 | 0 |
| OS6 | 8.5-9 | 84.6 | 100 | 0 | 2 | 0 | 15.4 | 0 | 0 |
| OS6 | 9-9.5 | 85.9 | 100 | 0 | 2.4 | 0 | 14.1 | 0 | 0 |
| PO1 | 0-0.5 | 0 | 100 | 0 | 3.9 | 0 | 23.4 | 0 | 100 |
| PO1 | 0.5-1 | 0.5 | 100 | 0 | 4 | 0 | 30.4 | 0 | 100 |
| PO1 | 1-1.5 | 85 | 100 | 0 | 1.3 | 0 | 14.9 | 0 | 0 |
| PO1 | 1.5-2 | 92.5 | 100 | 0 | 2.1 | 0 | 6.7 | 0 | 0 |
| PO1 | 2-2.5 | 87.6 | 100 | 0 | 1.7 | 0 | 12.3 | 0 | 0 |
| PO1 | 2.5-3 | 90.3 | 100 | 0 | 2.9 | 0 | 9.5 | 0 | 0 |
| PO1 | 3-3.5 | - | - | - | - | - | - | - | - |
| PO1 | 3.5-4 | 88.5 | 100 | 0 | 2 | 0 | 11.3 | 0 | 0 |
| PO1 | 4-4.5 | 86.3 | 100 | 0 | 2.5 | 0 | 13.4 | 0 | 0 |
| PO1 | 4.5-5 | - | - | - | - | - | - | - | - |
| PO1 | 5-5.5 | - | - | - | - | - | - | - | - |
| PO1 | 5.5-6 | - | - | - | - | - | - | - | - |
| PO1 | 6-6.5 | - | - | - | - | - | - | - | - |
| PO1 | 6.5-7 | - | - | - | - | - | - | - | - |
| PO1 | 7-7.5 | 89 | 100 | 0 | 1.8 | 0 | 10.9 | 0 | 0 |
| PO1 | 7.5-8 | 89.6 | 100 | 0 | 1.4 | 0 | 9.5 | 0 | 0 |
| PO1 | 8-8.5 | 89.9 | 100 | 0 | 1.5 | 0 | 9.9 | 0 | 0 |
| PO1 | 8.5-9 | 88.3 | 100 | 0 | 2.7 | 0 | 11.6 | 0 | 0 |
| PO1 | 9-9.5 | 79.4 | 100 | 0 | 3.9 | 0 | 20 | 0 | 0 |
| PO2 | 0-0.5 | 0 | 7.7 | 0 | 1.3 | 0 | 5.4 | 89 | 100 |
| PO2 | 0.5-1 | 0 | 100 | 0 | 8 | 0 | 44.8 | 0 | 99.9 |
| PO2 | 1-1.5 | 84.2 | 100 | 0 | 4 | 0 | 15.8 | 0 | 0 |
| PO2 | 1.5-2 | 91.4 | 100 | 0 | 2.1 | 0 | 8.6 | 0 | 0 |
| PO2 | 2-2.5 | 92.1 | 100 | 0 | 1.5 | 0 | 7.3 | 0 | 0 |
| PO2 | 2.5-3 | 92.3 | 100 | 0 | 1.3 | 0 | 7.6 | 0 | 0 |
| PO2 | 3-3.5 | 93.3 | 100 | 0 | 1.4 | 0 | 6.6 | 0 | 0 |
| PO2 | 3.5-4 | 92.4 | 100 | 0 | 1.2 | 0 | 7 | 0 | 0 |
| PO2 | 4-4.5 | 94.1 | 100 | 0 | 1.1 | 0 | 5.5 | 0 | 0 |
| PO2 | 4.5-5 | 92.2 | 100 | 0 | 1 | 0 | 7.7 | 0 | 0 |
| PO2 | 5-5.5 | 93.4 | 100 | 0 | 1.4 | 0 | 6.5 | 0 | 0 |
| PO2 | 5.5-6 | 93.1 | 100 | 0 | 1.3 | 0 | 5.5 | 0 | 0 |
| PO2 | 6-6.5 | 92.8 | 100 | 0 | 1.1 | 0 | 7.2 | 0 | 0 |
| PO2 | 6.5-7 | 85.5 | 100 | 0 | 2.4 | 0 | 14 | 0 | 0 |
| PO2 | 7-7.5 | 79.9 | 100 | 0 | 2.9 | 0 | 20.1 | 0 | 0 |
| PO2 | 7.5-8 | 84.7 | 100 | 0 | 1.9 | 0 | 15.3 | 0 | 0 |
| PO2 | 8-8.5 | 91.6 | 100 | 0 | 1.8 | 0 | 6.7 | 0 | 0 |
| PO2 | 8.5-9 | 91 | 100 | 0 | 1.6 | 0 | 9 | 0 | 0 |
| PO2 | 9-9.5 | 90.7 | 100 | 0 | 2 | 0 | 8.5 | 0 | 0 |
| PO3 | 0-0.5 | 0 | 100 | 0 | 1.9 | 0 | 12 | 0 | 100 |
| PO3 | 0.5-1 | 0.4 | 100 | 0 | 2.3 | 0 | 5.9 | 0 | 99.2 |
| PO3 | 1-1.5 | 83.9 | 100 | 0 | 2.2 | 0 | 15.3 | 0 | 0 |
| PO3 | 1.5-2 | - | - | - | - | - | - | - | - |
| PO3 | 2-2.5 | 85.5 | 100 | 0 | 2.1 | 0 | 14.5 | 0 | 0 |
| PO3 | 2.5-3 | 72.1 | 100 | 0 | 2.3 | 0 | 27.9 | 0 | 0 |
| PO3 | 3-3.5 | 85.5 | 100 | 0 | 3.3 | 0 | 14.2 | 0 | 0 |
| PO3 | 3.5-4 | 83.8 | 100 | 0 | 1.9 | 0 | 15.9 | 0 | 0 |
| PO3 | 4-4.5 | 86.7 | 100 | 0 | 1.8 | 0 | 13.2 | 0 | 0 |
| PO3 | 4.5-5 | 87.2 | 100 | 0 | 2.4 | 0 | 12.6 | 0 | 0 |
| PO3 | 5-5.5 | 85.7 | 100 | 0 | 1.6 | 0 | 14.2 | 0 | 0 |
| PO3 | 5.5-6 | 87.6 | 100 | 0 | 2.1 | 0 | 12.3 | 0 | 0 |
| PO3 | 6-6.5 | 87.4 | 100 | 0 | 2.5 | 0 | 12.2 | 0 | 0 |
| PO3 | 6.5-7 | 81.2 | 100 | 0 | 2.1 | 0 | 15.2 | 0 | 0 |
| PO3 | 7-7.5 | - | - | - | - | - | - | - | - |
| PO3 | 7.5-8 | - | - | - | - | - | - | - | - |
| PO3 | 8-8.5 | - | - | - | - | - | - | - | - |
| PO3 | 8.5-9 | - | - | - | - | - | - | - | - |
| PO3 | 9-9.5 | - | - | - | - | - | - | - | - |
| PO4 | 0-0.5 | 0 | 0.2 | 0 | 0 | 0 | 0.1 | 99.3 | 100 |
| PO4 | 0.5-1 | 0 | 7.5 | 0 | 0.8 | 0 | 1.2 | 92.3 | 100 |
| PO4 | 1-1.5 | 0 | 98.8 | 0 | 1.7 | 0 | 5 | 0 | 100 |
| PO4 | 1.5-2 | 0 | 100 | 0 | 0.6 | 0 | 20.5 | 0 | 99.9 |
| PO4 | 2-2.5 | 93.5 | 100 | 0 | 1.5 | 0 | 5.5 | 0 | 0 |
| PO4 | 2.5-3 | 95 | 100 | 0 | 0.6 | 0 | 4.5 | 0 | 0 |
| PO4 | 3-3.5 | 91.7 | 100 | 0 | 1.8 | 0 | 8.3 | 0 | 0 |
| PO4 | 3.5-4 | 92.8 | 100 | 0 | 1.2 | 0 | 7 | 0 | 0 |
| PO4 | 4-4.5 | 94.8 | 100 | 0 | 1.2 | 0 | 4.5 | 0 | 0 |
| PO4 | 4.5-5 | 94.2 | 100 | 0 | 1.1 | 0 | 5.2 | 0 | 0 |
| PO4 | 5-5.5 | 92.1 | 100 | 0 | 1.9 | 0 | 7.9 | 0 | 0 |
| PO4 | 5.5-6 | - | - | - | - | - | - | - | - |
| PO4 | 6-6.5 | 90.6 | 100 | 0 | 1.4 | 0 | 9.4 | 0 | 0 |
| PO4 | 6.5-7 | 92 | 100 | 0 | 2.2 | 0 | 7.9 | 0 | 0 |
| PO4 | 7-7.5 | 91 | 100 | 0 | 1.9 | 0 | 8.9 | 0 | 0 |
| PO4 | 7.5-8 | 91.5 | 100 | 0 | 2.1 | 0 | 8 | 0 | 0 |
| PO4 | 8-8.5 | 93.3 | 100 | 0 | 2.5 | 0 | 6.6 | 0 | 0 |
| PO4 | 8.5-9 | 92.1 | 100 | 0 | 2.1 | 0 | 7.5 | 0 | 0 |
| PO4 | 9-9.5 | 89.1 | 100 | 0 | 2.3 | 0 | 10.1 | 0 | 0 |

**SI References**

1. O. Temkin, *Soranus’ gynecology* (John Hopkins Press, 1956).

2. Galen, *Hygiene, Volume I: Books 1–4*, I. Johnston, Ed. (Harvard University Press, 2018).

3. Grant, “Dieting for an Emperor: A Translation of Books 1 and 4 of Oribasius’ *Medical Compilations* with an Introduction and Commentary” in *Dieting for an Emperor*, (Brill, 2018).

4. G. Pedrucci, “On the use of human milk and mestrual blood between medicine and magic in the Greek and Roman worlds” in *Ancient Magic: Then and Now*, 1st edition, A. Mastrocinque, J. E. Sanzo, M. Scapini, Eds. (Franz Steiner Verlag, 2020), pp. 287–302.

5. Empedocles, *The Fragments of Empedocles*, W. E. Leonard, Ed. (The open court publishing company, 1908).

6. C. Bourbou, S. J. Garvie-Lok, Breastfeeding and weaning patterns in Byzantine times: evidence from human remains and written sources. *Becoming Byzantine: Children and Childhood in Byzantium* 65–83 (2009).

7. L. M. V. Totelin, “Breastfeeding” in *The Encyclopedia of Ancient History*, R. S. Bagnall, K. Brodersen, C. B. Champion, A. Erskine, S. R. Huebner, Eds. (Wiley-Blackwell, 2016), pp. 1–2.

8. L. Totelin, “Weaning and Lactation Cessation in Late Antiquity and the Early Byzantine Period: Medical Advice in Context” in *Breastfeeding and Mothering in Antiquity and Early Byzantium*, (Routledge, 2023).

9. Caelius Aurelianus, *On acute diseases: and On chronic diseases*, I. E. Drabkin, Ed. (University of Chicago Press, 1950).

10. Pliny the Elder, *Natural History*, H. Rackham, W. H. S. Jones, D. E. Eichholz, Eds. (Harvard University Press ; W. Heinemann, 1949).

11. G. Pedrucci, *Maternità e allattamenti nel mondo greco e romano. Un percorso fra scienza delle religioni e studi sulla maternità* (Scienze e Lettere, 2018).

12. S. Jaeggi-Richoz, Lait de femme ou rien : l’alimentation lactée des nourrissons grecs et romains. *Annales de Bretagne et des Pays de l’Ouest. Anjou. Maine. Poitou-Charente. Touraine* 111–123 (2022). https://doi.org/10.4000/abpo.7833.

13. D. H. Ubelaker, *Human Skeletal Remains: Excavation, Analysis, Interpretation* (Aldine De Gruyter, 2008).

14. J. E. Buikstra, D. H. Ubelaker, *Standards for Data Collection from Human Skeletal Remains: Proceedings of a Seminar at the Field Museum of Natural History* (Arkansas Archeological Survey, 1994).

15. T. D. White, P. A. Folkens, *The Human Bone Manual*, 1° edizione (Academic Press, 2005).

16. A. Pellegrino, “La necropoli della via Ostiense ad Acilia” in *Dalle Necropoli Di Ostia: Riti Ed Usi Funerari*, (Soprintendenza archeologica di Ostia, 1999).

17. P. F. Rossi, A. Kjellström, “A Brief Osteological Overview of 30 Individuals from Anas Acilia Via del Mare” in *Life and Death in a Multicultural Harbour City: Ostia Antica From the Republic Through Late Antiquity*, Acta Instituti Romani Finlandiae., A. Karivieri, Ed. (Institutum Romanum Finlandiae, 2020).

18. C. O. Lovejoy, Dental wear in the Libben population: its functional pattern and role in the determination of adult skeletal age at death. *Am J Phys Anthropol* **68**, 47–56 (1985).

19. T. W. Todd, Age changes in the pubic bone. *American journal of physical anthropology* **4**, 1–70 (1921).

20. S. Brooks, J. M. Suchey, Skeletal age determination based on the os pubis: A comparison of the Acsádi-Nemeskéri and Suchey-Brooks methods. *Human Evolution* **5**, 227–238 (1990).

21. M. Y. İşcan, S. R. Loth, “Estimation of age and determination of sex from the sternal rib” in *Forensic Osteology: Advances in the Identification of Human Remains*, K. J. Reichs, W. M. Bass, Eds. (Charles C. Thomas, 1998), pp. 68–89.

22. L. Scheuer, S. M. Black, *Developmental juvenile osteology* (Academic Press, 2000).

23. S. J. AlQahtani, M. P. Hector, H. M. Liversidge, Brief communication: The London atlas of human tooth development and eruption. *Am. J. Phys. Anthropol.* **142**, 481–490 (2010).

24. E. Nikita, *Osteoarchaeology: A Guide to the Macroscopic Study of Human Skeletal Remains*, Illustrated edition (Academic Press, 2017).

25. E. Nikita, A. Karligkioti, *Basic Guidelines for the Excavation and Study of Human Skeletal Remains* (Zenodo, 2020).

26. T. D. White, M. T. Black, P. A. Folkens, *Human osteology*, 3rd ed (Academic Press, 2012).

27. S. Minozzi, A. Canci, *Archeologia dei resti umani. Dallo scavo al laboratorio* (Carocci, 2015).

28. F. Mallegni, B. Lippi, *Non omnis moriar* (CISU, 2009).

29. S. Vaccaro, I. Fiore, M. Lo Blundo, P. F. Rossi, Archivi biologici e Archivi di carta: I dati antropologici degli inumati dell’antemurale di Portus e il sistema di gestione dei resti umani antichi al Parco archeologico di Ostia antica. *Bollettino di Archeologia online* **XIV**, 21–62 (2023).

30. E. Ganiatsou, E. Vika, A. Georgiadou, T. Protopsalti, C. Papageorgopoulou, Breastfeeding and Weaning in Roman Thessaloniki. An Investigation of Infant Diet based on Incremental Analysis of Human Dentine. *Environmental Archaeology* 1–19 (2022). https://doi.org/10.1080/14614103.2022.2083925.

31. E. Ganiatsou, *et al.*, Application of machine learning on isotopic data from tooth microsections for reconstructing weaning patterns and physiological stress. *Journal of Archaeological Science: Reports* **47**, 103765 (2023).

32. C. Cocozza, R. Fernandes, A. Ughi, M. Groß, M. M. Alexander, Investigating infant feeding strategies at Roman Bainesse through Bayesian modelling of incremental dentine isotopic data. *International Journal of Osteoarchaeology* **31**, 429–439 (2021).

33. J. W. Eerkens, A. G. Berget, E. J. Bartelink, Estimating weaning and early childhood diet from serial micro-samples of dentin collagen. *Journal of Archaeological Science* **38**, 3101–3111 (2011).

34. J. Beaumont, J. Montgomery, Oral histories: a simple method of assigning chronological age to isotopic values from human dentine collagen. *Annals of Human Biology* **42**, 407–414 (2015).

35. R. C. Henderson, J. Lee-Thorp, L. Loe, Early life histories of the London poor using δ13C and δ15N stable isotope incremental dentine sampling. *Am. J. Phys. Anthropol.* **154**, 585–593 (2014).

36. I. Scharlotta, G. Goude, E. Herrscher, V. I. Bazaliiskii, A. W. Weber, “Mind the gap”-Assessing methods for aligning age determination and growth rate in multi-molar sequences of dietary isotopic data. *Am J Hum Biol* **30**, e23163 (2018).

37. A. Czermak, T. Fernández‐Crespo, P. W. Ditchfield, J. A. Lee‐Thorp, A guide for an anatomically sensitive dentine microsampling and age‐alignment approach for human teeth isotopic sequences. *Am J Phys Anthropol* **173**, 776–783 (2020).

38. C. Cocozza, R. Fernandes, Tooth formation age dataset for early childhood bioarchaeological and medical studies. *Data in Brief* **36**, 107141 (2021).

39. C. Dean, Extension rates and growth in tooth height of modern human and fossil hominin canines and molars. *Front Oral Biol* **13**, 68–73 (2009).

40. M. C. Dean, T. J. Cole, Human Life History Evolution Explains Dissociation between the Timing of Tooth Eruption and Peak Rates of Root Growth. *PLoS ONE* **8**, e54534 (2013).

41. R. Fernandes, A. R. Millard, M. Brabec, M.-J. Nadeau, P. Grootes, Food Reconstruction Using Isotopic Transferred Signals (FRUITS): A Bayesian Model for Diet Reconstruction. *PLoS ONE* **9**, e87436 (2014).

42. R. Fernandes, P. Grootes, M.-J. Nadeau, O. Nehlich, Quantitative diet reconstruction of a Neolithic population using a Bayesian mixing model (FRUITS): The case study of Ostorf (Germany). *American Journal of Physical Anthropology* **158**, 325–340 (2015).

43. R. Fernandes, A Simple(R) Model to Predict the Source of Dietary Carbon in Individual Consumers: A simple(r) model to predict the source of dietary carbon. *Archaeometry* **58**, 500–512 (2016).

44. C. Cocozza, E. Cirelli, M. Groß, W.-R. Teegen, R. Fernandes, Presenting the Compendium Isotoporum Medii Aevi, a Multi-Isotope Database for Medieval Europe. *Sci Data* **9**, 354 (2022).

45. A. Sołtysiak, R. Fernandes, Much ado about nothing: assessing the impact of the 4.2 kya event on human subsistence patterns in northern Mesopotamia using stable isotope analysis. *Antiquity* **95**, 1145–1160 (2021).

46. F. D. Pate, R. J. Henneberg, M. Henneberg, Stable Carbon And Nitrogen Isotope Evidence For Dietary Variability At Ancient Pompeii, Italy. *Mediterranean Archaeology and Archaeometry* **16**, 127–133 (2016).

47. T. C. O’Connell, *et al.*, Living and dying at the Portus Romae. *Antiquity* **93**, 719–734 (2019).

48. E. Nitsch, *et al.*, A bottom-up view of food surplus: using stable carbon and nitrogen isotope analysis to investigate agricultural strategies and diet at Bronze Age Archontiko and Thessaloniki Toumba, northern Greece. *World Archaeology* **49**, 105–137 (2017).

49. O. E. Craig, *et al.*, Stable isotopic evidence for diet at the Imperial Roman coastal site of Velia (1st and 2nd Centuries AD) in Southern Italy. *Am. J. Phys. Anthropol.* **139**, 572–583 (2009).

50. S. Soncin, *et al.*, High-resolution dietary reconstruction of victims of the 79 CE Vesuvius eruption at Herculaneum by compound-specific isotope analysis. *Science Advances* (2021). https://doi.org/10.1126/sciadv.abg5791.

51. S. Verd, G. Ginovart, J. Calvo, J. Ponce-Taylor, A. Gaya, Variation in the Protein Composition of Human Milk during Extended Lactation: A Narrative Review. *Nutrients* **10**, 1124 (2018).

52. G. E. Leghi, *et al.*, Daily variation of macronutrient concentrations in mature human milk over 3 weeks. *Sci Rep* **11**, 10224 (2021).

53. Y. Zhang, *et al.*, Comparative Proteomic Analysis of Proteins in Breast Milk during Different Lactation Periods. *Nutrients* **14**, 3648 (2022).

54. R. Fernandes, M.-J. Nadeau, P. M. Grootes, Macronutrient-based model for dietary carbon routing in bone collagen and bioapatite. *Archaeol Anthropol Sci* **4**, 291–301 (2012).

55. World Health Organization, *WHO recommendations on postnatal care of the mother and newborn* (World Health Organization, 2014).

56. World Health Organization, United Nations Children’s Fund (UNICEF), “Weaning : from breast milk to family food, a guide for health and community workers” (World Health Organization, 1988).

57. M. S. Kramer, Does breast feeding help protect against atopic disease? Biology, methodology, and a golden jubilee of controversy. *The Journal of Pediatrics* **112**, 181–190 (1988).

58. A. I. Eidelman, *et al.*, Breastfeeding and the Use of Human Milk. *Pediatrics* **129**, e827–e841 (2012).
